# Supplementary material for: The relationship between mental health and risk of active tuberculosis: a systematic review
Source: BMJ Open. 2022 Jan 6;12(1):e048945. doi: 10.1136/bmjopen-2021-048945 (PMC8739435; doi:10.1136/bmjopen-2021-048945)
Supplement: Supplementary data [file bmjopen-2021-048945supp001.pdf]

Supplementary Material

Supplementary Box 1: Search Strategy

MEDLINE Search Strategy:

1. ((Mental or Mentally or Psychological or Psychiatric) adj3 (health or illness* or disorder* or ill or unwell)).mp [mp=title, abstract, original title, name of substance word, subject heading word, keyword heading word, protocol supplementary concept word, rare disease supplementary concept word, unique identifier, synonyms]
2. exp Mental Health/
3. exp Mental Disorders/ep, et, mo, px, sn [Epidemiology, Etiology, Mortality, Psychology, Statistics & Numerical Data]
4. 1 or 2 or 3
5. (Tuberculosis or TB).mp. [mp=title, abstract, original title, name of substance word, subject heading word, keyword heading word, protocol supplementary concept word, rare disease supplementary concept word, unique identifier, synonyms]
6. exp Tuberculosis/ep, et, mo, px, sn, tm [Epidemiology, Etiology, Mortality, Psychology, Statistics & Numerical Data, Transmission]
7. 5 or 6
8. 4 and 7
9. limit 8 to English language

The PsycINFO search was the same as the MEDLINE search presented, except using subject headings for PsycINFO instead of MeSH terms. The PsycEXTRA search used the same free text terms, but this database does not use subject headings.

Hayward SE, *et al. BMJ Open* 2022; 12:e048945. doi: 10.1136/bmjopen-2021-048945

Supplementary Table 1: Critical appraisal

PART 1: Critical appraisal of cohort and case-control studies

**Cohort**	**Oh et al 2017**	**Cheng et al 2017**	**Kuo et al 2013**	**Ohta et al 1988**	**Case-control**	**de Araújo et al 2014**
**Are the results of the study valid?**						
1. Did the study address a clearly focused issue?	✓ To determine the association between depression and risk of TB	✓ To determine the association between depression and risk of pulmonary TB	✓ To determine the association between schizophrenia and risk (and outcome) of TB	✓ To investigate the incidence of TB among schizophrenics compared with the general population in Japan	1. Did the study address a clearly focused issue?	✓ To investigate the association between common mental disorders and risk of TB
2. Was the cohort recruited in an acceptable way?	✓ Nationwide population-based database, so less selection/recall bias and large sample size<br>Controls were more likely to have comorbidities than the general population as they were selected from a medical claims database – but comorbidities were adjusted for	✓ Nationwide population-based database, so less selection/recall bias and large sample size	✓ Nationwide population-based database, so less selection/recall bias and large sample size	✓ Registry of schizophrenics, so less selection/recall bias (but misses undiagnosed/ unregistered schizophrenics)	2. Did the authors use an appropriate method to answer their question?	✓ Cohort study would have provided stronger evidence for the directionality of association
3. Was the exposure accurately measured to minimise bias?	✓ ICD-10 codes for depression (requires psychiatrist's judgement from long-term observation), confirmed by prescription of psychotherapy – all psychiatrists in South Korea prescribe psychotherapy each time they see a patient under the fee-for-service system, so cases diagnosed as mild depression were also included	✓ ICD-9 codes for depression (but insurance claims not definitive measurement)	✓ ICD-9 code for schizophrenia, further validated if it was coded by psychiatrists (not clear whether this is a requirement for inclusion)	Can't tell – diagnostic criteria for schizophrenia not stated<br>Not clear whether authors are including incident or prevalent schizophrenia cases	3. Were the cases recruited in an acceptable way?	✓ Individuals diagnosed with pulmonary TB by attending chest physician at a health care unit in Salvador – excludes those who do not attend these clinics<br>Evidence of power calculation
4. Was the outcome accurately measured to minimise bias?	✓ ICD-10 codes for TB diagnosis, confirmed by prescription of two or more anti-TB drugs for simultaneous use for >30 days	✓ ICD-9 codes for pulmonary TB (but insurance claims not definitive measurement)	✓ ICD-9-CM codes for TB, plus prescription of at least two anti-TB drugs – but some bias may be introduced as any cases not prescribed anti-TB drugs will not be included	Can't tell – diagnostic criteria for TB not stated	4. Were the controls selected in an acceptable way?	× Symptomatic respiratory patients who were excluded from diagnosis of TB, age- and sex-matched – but are these patients representative of the general population?
5. a) Have the authors identified all important	× Age, gender, comorbidities (DM, COPD, alcoholism), income level	× Sex, age, comorbidities (comprehensive, but measured at baseline not during follow up)	× Age, gender, comorbidities (comprehensive)<br>But did not include any measure of SEP	× Only age and sex	5. Was the exposure accurately measured to minimise bias?	✓ Self-Reporting Questionnaire (SRQ-20), a well-recognised instrument, validated in Brazil

Cohort	Oh et al 2017	Cheng et al 2017	Kuo et al 2013	Ohta et al 1988	Case-control	de Araújo et al 2014
confounding factors?	Could have included more comorbidities	But did not include any measure of SEP				Instrument applied before diagnosis of TB
b) Have they taken account of the confounding factors in the design and/or analysis?	✓ Matched or included in multivariable Cox proportional hazards model	✓ Matched or included in multivariable Cox proportional hazards model	✓ Matched or included in multivariable Cox proportional hazards model	× Separate RRs calculated by sex and born before/after 1925, but no other factors considered and RR is not adjusted	6. a) What confounding factors have the authors accounted for?	✓ Marital status, ethnicity, history of contact with TB, diabetes, monthly family income, type of residence, number of household goods, level of education, crowding, recreational drugs, smoking, and alcohol consumption
6. a) Was the follow up of subjects complete enough?	✓ Nationwide database	✓ Nationwide database	✓ Nationwide database	✓ All TB cases must legally have been registered to public health centers in Nagasaki	b) Have they taken account of the potential confounding factors in the design and/or in their analysis?	✓ Matched or included in multivariable conditional logistic regression model
b) Was the follow up of subjects long enough?	✓ Followed up over up to 11 years	✓ Mean follow up 8.21 years depression and 8.30 years controls	✓ Median follow up 2,368 days for schizophrenics and 2,296 for controls	✓ (Up to?) 19 years		
**What are the results?**						
7. What are the results of this study?	Patients with depression are at a higher risk for TB (HR=2.63), with a dose-response relationship	Patients with depression are at a higher risk for TB (HR=1.15)	Schizophrenics are at a higher risk for TB (HR=1.52)	RR for TB incidence observed in those with schizophrenia vs. expected based on general population rates = 3.04	7. What are the results of this study?	Statistically significant association between overall CMDs and TB (adjusted OR=1.34)
8. How precise are the results?	HR=2.63 (95% CI 1.74-3.96)	HR=1.15 (95% CI 1.03-1.28)	HR=1.52 (95% CI 1.29-1.79, P<0.001)	No CIs given, P<0.005	8. How precise are the results? How precise is the estimate of risk?	Adjusted OR=1.34 (95% CI 1.05-1.70)<br>Overall P value not given
9. Do you believe the results?	✓ Big effect (CI does not cross 1), dose-response, study design seems sound	✓ Although effect isn't huge, CI is quite narrow and does not cross 1 (just), study design seems sound	✓ Although effect isn't huge, CI is reasonably narrow and does not cross 1, study design seems sound	✓ I believe that TB incidence is higher in schizophrenics than the general population (big effect), but to be able to say that there is an association would have been preferable to have controls and account for confounders Also would have been better to use rate per person-years	9. Do you believe the results?	✓ Although effect isn't huge, CI is quite narrow and does not cross 1 (just)
**What are the implications?**						
10. Do the results have good external validity?	✓ General population of South Korea likely to be largely representative of other HICs	✓ General population of Taiwan likely to be largely representative of other MICs/HICs	✓ General population of Taiwan likely to be largely representative of other MICs/HICs	✓ Population of Japan likely to be largely representative of other HICs	10. Do the results have good external validity?	× While the presence of an association is informative, the OR will only be relevant to this particular comparison with other respiratory patients
11. Do the results of this study fit with	✓ Depression as a risk factor for TB	✓ Depression as a risk factor for TB	✓ Schizophrenia as a risk factor for TB	✓ Schizophrenia as a risk factor for TB	11. Do the results of this study fit with	✓ Association between CMDs and TB

Cohort	Oh et al 2017	Cheng et al 2017	Kuo et al 2013	Ohta et al 1988	Case-control	de Araújo et al 2014
other available evidence?					other available evidence?	
12. What are the implications of this study for the review?	Supports conclusion that mental health is a risk factor for TB	Supports conclusion that mental health is a risk factor for TB	Supports conclusion that mental health is a risk factor for TB	Supports conclusion that mental health is a risk factor for TB	12. What are the implications of this study for the review?	Supports conclusion that mental health is associated with TB
**Overall judgement**						
Score	10/11 (91%)	10/11 (91%)	10/11 (91%)	7/11 (64%)	Score	8/10 (80%)
Overall study quality	High	High	High	Moderate	Overall study quality	Moderate

PART 2: Critical appraisal of cross-sectional studies

Cross-sectional	Koyanagi et al 2017	Hernández Sarmiento et al 2013	Lasebikan & Ige 2015	Srivastava et al 1983	de Castro-Silva et al 2019
**Introduction**					
1. Were the aims/ objectives of the study clear?	✓ To assess the association between depression and TB (and impact on health status) in LMICs	✓ To assess TB incidence, transmission patterns and association with socio-demographic factors and mental disorders in Colombian homeless people	✓ To determine the prevalence of psychosis in TB patients compared with controls (and its correlation with disease pattern)	✓ To discuss the psychological symptoms encountered in TB	✓ To estimate the prevalence of current major depressive episode (MDE) among patients with presumptive pulmonary TB (PTB) and compare it between patients with and without PTB
**Methods**					
2. Was the study design appropriate for the stated aim(s)?	✓ Cohort study would have provided evidence for the directionality of association	✓ Cohort study would have provided evidence for the directionality of association	✓ Cohort study would have provided evidence for the directionality of association	✓ Cohort study would have provided evidence for the directionality of association	✓ Cohort study would have provided evidence for the directionality of association
3. Was the sample size justified?	✓ Large sample (242,952), sample size determined by data available	× No justification given (relatively small sample size of 426)	× No justification given (small sample size of 227)	× No justification given (very small sample size of 120)	× No justification given (relatively small sample size of 260)
4. Was the target/reference population clearly defined? (Is it clear who the research was about?)	✓ General population of LMICs	✓ Homeless people in Medellín, Columbia	✓ Ibadan, Nigeria	✓ Population of NW districts of Rajasthan and adjoining districts of Punjab and Haryana states	✓ Presumptive pulmonary TB patients at a municipal health center in Rio de Janerio, Brazil
5. Was the sample frame taken from an appropriate population base so that it closely represented the target/reference population under investigation?	× Large sample size, predominantly nationally representative data (except 6 countries)<br>But eligible participants >18 years and have a valid home address – excludes those without a valid home address, e.g. homeless and institutionalised who are at a high risk of TB	× Homeless people who attended a local health entity in Medellín city, so excludes those who do not attend the facility	× Those attending the MDR-TB clinic may not be representative of all TB patients in Ibadan, and their family members/caregivers may not be representative of general population	× Hospital for TB and chest diseases, Bikaner, so excludes those who are undiagnosed or not admitted to this hospital	× Sample is presumptive pulmonary TB patients (presenting with a cough lasting 3 weeks or longer), so controls without TB may not be representative of general population
6. Was the selection process likely to select	✓ Single-stage random sampling (10 countries)	× Non-probabilistic sampling	× Exclusion criteria may introduce bias e.g. excluding	× Extensive exclusion criteria e.g. patients whose general condition	✓ Exclusion criteria are reasonable

Hayward SE, *et al. BMJ Open* 2022; 12:e048945. doi: 10.1136/bmjopen-2021-048945

Cross-sectional	Koyanagi et al 2017	Hernández Sarmiento et al 2013	Lasebikan & Ige 2015	Srivastava et al 1983	de Castro-Silva et al 2019
subjects/participants that were representative of the target/reference population under investigation?	Multi-stage random cluster sampling (60 countries)		those who were not literate in English or Yoruba and those with any affective disorder, affective psychosis or delirium, and excluding family members/caregivers with a past or current history of TB or any psychiatric disorder	was considered to be poor by the treating physician and patients who gave a history of previous psychotic illness or drug abuse	
7. Were measures undertaken to address and categorise non-responders?	✓ Sampling weights generated to adjust for non-response	× No information given on response rate or non-responders	× No information given on response rate or non-responders	× No information given on response rate or non-responders	× No data available on characteristics of non-responders
8. Were the risk factor and outcome variables measured appropriate to the aims of the study?	✓ Risk factor – depressive symptoms<br>Outcome – TB	✓ Risk factor – mental disorders<br>Outcome – pulmonary TB	✓ Risk factor – psychosis<br>Outcome – MDR-TB	✓ Risk factor – psychological state<br>Outcome – pulmonary TB	✓ Risk factor – depressive symptoms, major depressive episode<br>Outcome – pulmonary TB
9. Were the risk factor and outcome variables measured correctly using instruments/measurements that had been trialled, piloted or published previously?	× Risk factor – World Mental Health Survey version of the Composite International Diagnostic Interview (used previously)<br>Outcome – past 12-month symptoms of active TB (used previously, but sensitivity 65-70% and specificity 55-75% in detecting TB)	✓ Risk factor – Mini-International Neuropsychiatric Interview (pilot experiment)<br>Outcome – medical screening examination, sputum sample analysed in those with compatible symptoms	✓ Risk factor – GHQ-12 to screen for psychiatric morbidity (good internal consistency, validated and used in Nigeria), followed by psychosis screening questionnaire for those who screened positive (valid, used in Nigeria) and psychosis module of SCID<br>Outcome – patients attending the MDR-TB treatment center (culture and drug susceptibility test-proven cases)<br>Pilot study carried out on 15 patients and 15 relatives in a different hospital in Ibadan	✓ Risk factor – Present State Examination (PSE), no evidence of having been validated<br>Considering that if a patient has symptoms in all 6 PSE subgroups he/she must have a diagnosable psychiatric illness not justified<br>Symptoms included difficulty in breathing, weight loss – but surely this is more likely due to the TB?<br>Outcome – TB diagnostic criteria not stated	✓ Risk factor – PHQ-9 and MINI-Plus; both are recognised tools and PHQ-9 has been validated in Brazil; administered by trained interviewers<br>Outcome – pulmonary TB diagnosed following the recommendations of the Brazilian national TB program
10. Is it clear what was used to determined statistical significance and/or precision estimates? (e.g. p-values, confidence intervals)	✓ Level of statistical significance set at P<0.05<br>P values and 95% CIs reported	✓ Level of statistical significance set at P<0.05<br>P values and 95% CIs reported	✓ Level of statistical significance set at P<0.05<br>P values reported	× Not clear, but from table it seems that level of statistical significance was set at P<0.05	✓ Level of statistical significance set at P<0.05<br>P values and 95% CIs reported
11. Were the methods (including statistical methods) sufficiently described to enable them to be repeated?	✓ Detailed description of methods, with references to other publications with further details	× Methods of enrolment inadequately described (need for clarification of 'non-probabilistic sample')	× Methods somewhat unclear regarding patient recruitment (consecutive? random?), screening for psychosis (psychosis screening questionnaire vs. SCID), and statistical analysis (how were ORs calculated?)	× Methods of recruitment not clear (1-2 patients out of ~5 admissions per day, no mention of how controls were recruited), no information on TB diagnostic criteria, no explanation of statistical methods used	✓ Detailed description of methods

Hayward SE, *et al. BMJ Open* 2022; 12:e048945. doi: 10.1136/bmjopen-2021-048945

Cross-sectional	Koyanagi et al 2017	Hernández Sarmiento et al 2013	Lasebikan & Ige 2015	Srivastava et al 1983	de Castro-Silva et al 2019
			It is stated that participants were periodically reviewed to detect new cases of psychosis while on admission in the MDR treatment facility, but it is not clear how this is included in the analysis		
**Results**					
12. Were the basic data adequately described?	✓ Detailed table of sample characteristics	✓ Detailed table of socio-demographic variables	✓ Detailed table of socio-demographic characteristics of patients and family members/caregivers	× Only information on age and gender given	✓ Detailed tables of socio-demographic variables
13. Does the response rate raise concerns about non-response bias?	✓(No) Response rate 98.5% (but there is substantial missing data for some variables, >10% for TB, BMI and diabetes)	×(Yes) Response rate not stated	×(Yes) Response rate not stated	×(Yes) No information given on response rate	×(Yes) Of 3,251 presumptive PTB patients, only 260 were included in the sample
14. If appropriate, was information about non-responders described?	× But it is reported that sampling weights were generated using the population distribution to adjust for non-response, so this is not a big concern	× No information given on non-responders	× No information given on non-responders	× No information given on non-responders	× No data available on characteristics of non-responders
15. Were the results internally consistent?	✓ No apparent inconsistencies	✓ No apparent inconsistencies	✓ No apparent inconsistencies	× Number in each age group adds up to 70, when sample size is 60	× It is reported in the text that 98/260 participants were diagnosed with active PTB, but in Table 1 this is reported as 99/260
16. Were the results presented for all the analyses described in the methods?	✓ Yes	× In the bivariate and multivariate analysis only significant associations (between socio-demographics/psychiatric disorders and PTB) were reported	× It seems that only ORs that gave significant results were reported when looking at correlates of psychosis in TB patients	× Statistical methods were not described<br>Other PSE symptoms that did not reach statistical significance were not reported<br>Some findings seem to be alluded to in the discussion that were not reported in the results	× Sociodemographics only presented for factors that are associated with diagnosis of TB or current MDE, so some variables not reported e.g. family income
**Discussion**					
17. Were the authors' discussions and conclusions justified by the results?	✓ TB is associated with the entire depression spectrum in the overall sample, and the association is comparable across regions and country income levels<br>Lots of potential confounders included in analysis (although could have included more comorbidities)	✓ Homeless people are a population with a high TB prevalence which co-exists with a high rate of mental disorders<br>Seems a fair conclusion, and the authors also recognise that dysthymia is the only psychiatric disorder that remains associated with risk of TB when adjusting for confounders	× Prevalence of psychosis among TB patients in this study is 33.0% compared to 2.7% among primary care givers<br>Without any adjustment for confounding it is hard to draw any conclusions from this figure, especially considering substantial baseline imbalances	✓ Significantly more patients as compared to control subjects are worried, anxious, depressed, irritable and have sleep and appetite disturbances<br>While this statement is true in this study, it is not possible to say whether there is an association here without accounting for confounding	✓ The prevalence of MDE (detected by the PHQ-9) among individuals with pre-PTB was high, and there was little difference between individuals with confirmed PTB and those with other respiratory illnesses (60.2 vs. 62.1%, respectively)<br>However, as controls have other respiratory illnesses it is not

**Cross-sectional**	**Koyanagi et al 2017**	**Hernández Sarmiento et al 2013**	**Lasebikan & Ige 2015**	**Srivastava et al 1983**	**de Castro-Silva et al 2019**
		No adjustment for comorbidities is an issue<br>The significant results that arise may be due to multiple testing			possible to say the prevalence of depression is the same or different in TB patients compared with the general population
18. Were the limitations of the study discussed?	✓ Lacking information on HIV, based on TB symptoms not lab diagnosis, exclusion of institutionalised and homeless	✓ Difficulty differentiating primary psychotic and affective symptoms from secondary psychotic symptoms, only patients from one homeless shelter	✓ Small sample size, only those who scored positively on GHQ were assessed for psychosis, exclusion of patients with any affective disorder, affective psychosis or delirium, only one treatment centre included	× Limitations not discussed	✓ Loss of subjects who did not return for sputum culture, small sample size, inability to analyse causal relationships, MINI-Plus only used on subset of individuals
**Other**					
19. Were there any funding sources or conflicts of interest that may affect the authors' interpretation of the results?	✓(No) Funders had no role in study design, data collection, analysis and interpretation, or in writing manuscript	✓(No) None declared	✓(No) No conflict of interest declared (but perhaps there is one, since funder is Pan African Mental Health Initiative, Nigeria?)	×(Possibly) Not stated	✓(No) No conflict of interest declared
20. Was ethical approval or consent of participants attained?	✓ All participants gave informed consent	✓ All participants signed informed consent	✓ Only consenting participants included	× Not stated	✓ All participants signed informed consent
**Overall judgement**					
Score	17/20 (85%)	12/20 (60%)	11/20 (55%)	5/20 (25%)	13/20 (65%)
Overall study quality	Moderate	Low	Low	Low	Moderate

Hayward SE, *et al. BMJ Open* 2022; 12:e048945. doi: 10.1136/bmjopen-2021-048945

Supplementary Table 2: Details of Included Studies

PART 1	Oh et al 2017	Cheng et al 2017	Kuo et al 2013	Ohta et al 1988	de Araújo et al 2014
**General Information**					
Report title	Depression and risk of tuberculosis: a nationwide population-based cohort study	Increased Risk of Pulmonary Tuberculosis in Patients with Depression: A Cohort Study in Taiwan	Incidence and outcome of newly-diagnosed tuberculosis in schizophrenics: a 12-year, nationwide, retrospective longitudinal study	The Epidemiological Study of Physical Morbidity in Schizophrenics—2. Association between Schizophrenia and Incidence of Tuberculosis	Common Mental Disorders Associated with Tuberculosis: A Matched Case-Control Study
Study funding source	Not stated	Taiwan Ministry of Health and Welfare Clinical Trial Center, China Medical University Hospital, Academia Sinica Taiwan Biobank Stroke Biosignature Project, Taiwan Clinical Trial Consortium for Stroke, Tseng-Lien Lin Foundatin, Taichung, Taiwan, Taiwan Brain Disease Foundation, Taipei, Taiwan, and Katsuzo and Kiyo Aoshima Memorial Funds, Japan	National Health Research Institute, Taipei Veterans General Hospital, and the National Science Council	Not stated	National Council for Scientific and Technological Development
Possible conflicts of interest	None declared	Not stated	None declared	Not stated	None declared
**Study Characteristics**					
Type of study	Cohort, retrospective	Cohort, retrospective	Cohort, retrospective	Cohort, retrospective (longitudinal registry)	Matched case-control
Exposure	Depression	Depression	Schizophrenia	Schizophrenia	Common Mental Disorders
Outcome	TB incidence	Pulmonary TB incidence	TB incidence (and outcomes)	TB incidence	Pulmonary TB cases
**Population and Setting**					
Population description	South Korean population who made claims through the National Health Insurance Service	Taiwanese population covered by the National Health Insurance Program	Taiwanese population covered by National Health Insurance program	Those with schizophrenia living in Nagasaki, Japan	Symptomatic respiratory patients
Setting	Korean National Health Insurance Service-National Sample Cohort (NHIS-NSC), which sampled 1 million subjects with national representativeness from among the entire population covered by NHIS in 2002	Database of the Taiwan National Health Insurance Program	NHI Research Database (NHIRD), which is run by the National Health Research Institute	Nagasaki, Japan	Three referral hospitals and six community clinics in Salvador, Brazil
Inclusion criteria	Patients with depression newly diagnosed between 2003 and 2013 were selected from the NHIS-NSC to form the exposure cohort	Subjects aged 20-84 years with newly diagnosed depression from 2000 to 2012 selected into the depression group	Schizophrenic cohort comprised all patients who were admitted with schizophrenia from 1 Jan 1998 to 31 Dec 2009	All schizophrenic patients who resided in Nagasaki and were diagnosed as schizophrenic between 1960 and 1978	Individuals living in Salvador who were older than 14 years and who presented with respiratory symptoms and were investigated

PART 1	Oh et al 2017	Cheng et al 2017	Kuo et al 2013	Ohta et al 1988	de Araújo et al 2014
	The control cohort comprised the same number of individuals without any mood disorders, with each individual age- and sex-matched to a patient in the exposure cohort	Four subjects without depression randomly selected, for each subject with depression, into the non-depression group, matched for sex, age (every 5-year interval), and comorbidities	Controls randomly selected from the Longitudinal Health Insurance Database dataset, which contains complete data of 1,000,000 randomly sampled beneficiaries from the original NHIRD, to match schizophrenia patients on age (within 2 years), gender, index date, Charlson's score, chronic pulmonary disease, diabetes, and rheumatoid disease		for TB at any of several health care units
Exclusion criteria	Patients with a diagnosis of TB who used medical services in 2002 were excluded to rule out chronic conditions of depression or TB<br>Patients diagnosed with TB before the diagnosis of depression were excluded from the exposure cohort	Not stated	Schizophrenia group: patients who were diagnosed with TB before being admitted with schizophrenia were excluded<br>Control group: patients who were diagnosed with schizophrenia and antecedent TB were excluded	None stated	Those reporting a previous history of active TB
Method/s of recruitment	NHIS-NSC database	Database of the Taiwan National Health Insurance Program	Inpatient and outpatient claims data from NHIRD (National Health Insurance Research Database) in Taiwan	Registry of schizophrenic patients (from all psychiatric institutes in Nagasaki city and its suburbs)	All cases asked to participate, and the first eligible control who was an acceptable age and sex match was recruited into the study
**Methods**					
Aim of study	To determine the association between depression and risk of TB	To examine the causative relationship between depression and pulmonary TB in Taiwan	To compare the adjusted incidence and outcome of TB diseases in schizophrenics and the general population	To investigate the incidence of TB among schizophrenics	To investigate the association between common mental disorders and TB
Design	Retrospective cohort study using a nationwide database to identify incidence of TB in those with depression vs. controls	Retrospective cohort study using a nationwide database to identify incidence of TB in those with depression vs. controls	Retrospective cohort study using a nationwide database to identify incidence of TB in those with schizophrenia vs. controls	Registry data to identify TB incidence in those diagnosed with schizophrenia (1960-1978) compared with expected incidence based on annual incidence rate	Matched case-control study
Start date	2003	2000	January 1998	1960	August 2008
End date	2013	2013	December 2009	1978	April 2010
Duration of participation	Up to 11 years	All subjects followed up until they were diagnosed with pulmonary TB or to the end of 2013 – so up to 14 years, but mean follow-up is 8.21 years for depression and 8.30 for controls	From index date until end of December 2009, death, or diagnosis of TB (so up to 12 years) – median follow-up 2368 days (6.48 years)	(Up to?) 19 years	N/A
**Participants**					
Total no.	64,744	172,952	120,818	3251	1434 (717 cases, 717 controls)

PART 1	Oh et al 2017	Cheng et al 2017	Kuo et al 2013	Ohta et al 1988	de Araújo et al 2014
Baseline imbalances	See below	See below	Imbalances in some of the comorbidities that weren't matched e.g. drug or substance abuse 4.5% in schizophrenics vs. 0.02% in controls	Not stated	No differences in the matching variables, not stated for other variables
Withdrawals and exclusions	24 excluded as they were diagnosed with TB before the diagnosis of depression	Not stated	Not stated	Not stated	All individuals agreed to participate
Age	Mean 45.8 (SD 18.4 years)	Mean 47.9 (SD 16.5) for depression and 47.6 (16.6) for controls	Median 35.4 for schizophrenics and 35.3 for controls	More than 60% patients in their 20s to 40s when diagnosed with TB	Mean 38.2 (SD 14.2 years)
Sex	Female predominant (66%)	Male predominant (62.3%) in depression and controls	Male predominant (55.1%)	Male predominant (54.8%)	Male predominant, in cases (61.0%), and controls (60.5%)
Severity of illness	Ranging from mild to severe	Not stated	Not stated	Not stated	Not stated
Co-morbidities	0.6% DM in exposed vs. 22.6% in controls<br>0.1% COPD in exposed vs. 40.4% in controls<br>3.4% alcoholism in exposed vs. 0.6% in controls	Depression group had a higher proportion of asbestosis, CKD, HIV infection, gastrectomy, pneumoconiosis, and splenectomy than controls	Most (93.4% schizophrenics, 93.4% controls) did not have any severe comorbidities (Charlson's score < 3); most prevalent underlying diseases were peptic ulcer diseases (19.1% s, 18.0% c), chronic pulmonary disease (16.7% s, 16.7% c), and liver disease (12.9% s, 14.2% c)	Not stated	Not stated
Treatment received	Psychotherapy and anti-TB drugs (see below)	Not stated	Prescription of at least two anti-TB drugs (e.g., isoniazid, ethambutol, rifampin, pyrazinamide) for two months	Not stated	Not stated
Other relevant sociodemographics	22.4% low-income subjects in exposed vs. 25.1% in controls	Not stated	Not stated	Not stated	Not stated
Subgroups measured/reported	'Mild' and 'severe' depression	Sex, age (20-39, 40-64, 65-84), no comorbidity vs. any comorbidity	Age, gender, comorbidities (Charlson's score, diabetes, chronic pulmonary disease, rheumatoid disease, myocardial infarction, heart failure, peripheral vascular disease, peptic ulcer disease, liver disease, hemiplegia or paraplegia, chronic kidney disease, cancer, hypertension, dyslipidemia, arrhythmia, drug or substance abuse, and AIDS)	Sex, patients with schizophrenia born before and after 1925	Each of the dimensions of the SRQ-20
**Exposure**					
No. in each group	32,372 with depression, 32,372 controls	34,765 with depression, 138,187 controls	60,409 with schizophrenia, 60,409 controls	3251 with schizophrenia	513 with CMD (278 cases, 235 controls), 921 without CMD (439 cases, 482 controls)

PART 1	Oh et al 2017	Cheng et al 2017	Kuo et al 2013	Ohta et al 1988	de Araújo et al 2014
Measurement	ICD-10 codes for depression, confirmed by prescription of psychotherapy	ICD-9 codes for depression	Patients who were admitted with schizophrenia (ICD-9-CM code), validated if it was coded by psychiatrists	All patients who resided in the city and were diagnosed with schizophrenia between 1960 and 1978, using registry data from all psychiatric institutes in Nagasaki city and its suburbs	Self-Reporting Questionnaire (SRQ-20) to identify CMDs, individuals who scored above 7 were considered to have a CMD<br>Measured by a team of nursing technicians under the supervision of senior nurses
**Outcome**					
Time points measured/reported	2003-2013	2000-2013	1998-2009	1960-1978	Before the doctor diagnosed the presence or absence of TB
Outcome definition	ICD-10 codes for TB diagnosis, confirmed by prescription of two or more anti-TB drugs for simultaneous use for >30 days	New diagnosis of pulmonary TB during the follow-up period<br>ICD-9 codes for pulmonary TB	ICD-9-CM codes for TB, plus prescription of at least two anti-TB drugs for two months	All cases of TB (pulmonary and extrapulmonary) in the population have been registered at the public health centres in Nagasaki city according to certain legal procedures	Individuals diagnosed with pulmonary TB by smear microscopy and culture for *M. tb*<br>Controls selected from the symptomatic respiratory patients who were excluded from a diagnosis of TB
Person measuring/ reporting	Not stated	Not stated	Not stated	Not stated	Attending chest physician
**Results**					
Comparison	Depression vs. control cohort	Depression vs. control cohort	Schizophrenia vs. controls	Schizophrenia vs. general population	Those with CMD vs those without CMD
Outcome	TB incidence	Pulmonary TB incidence	New TB cases	TB incidence	Incident TB
Time point	Up to end of 2013	Up to end of 2013	Up to December 2009	1960-1978	Before the doctor diagnosed the presence or absence of TB
Results	Depression: 101 per 167,271 py (n=32,372)<br>No depression: 191 per 359,265 py (n=32,372)	Depression: 435 per 285,537 py (n=34,765)<br>No depression: 1504 per 1146,960 py (n=138,187)	Schizophrenia: 366 per 392,109 py (n=60409)<br>No schizophrenia: 241 per 379,548 py (n=60409)	Schizophrenia: 82 per 3251<br>Expected: 26.94 per 3251	CMDs: 278 per 513<br>No CMDs: 439 per 921
No. missing participants and reasons	Not stated	Not stated	Not stated	Not stated	Not stated
Any other results reported	TB incidence rate 60/100,000 py in depression cohort vs. 53/100,000 py in control cohort (P<0.0001 log-rank test)<br>Incidence rate ratio (IRR) of depression to non-depression in patients with TB = 1.14 (95% CI 0.89-1.45)<br>Risk of TB 2.63-fold (95% CI 1.74-3.96) higher in depression vs. control cohort (Cox)	Overall incidence of pulmonary TB was 1.16-fold greater in the depression group vs controls (1.52 vs. 1.31 per 1000 person years), i.e. IRR depression vs. non-depression = 1.16 (95% CI 1.12-1.21)<br>Kaplan-Meier model revealed that the depression group had a higher cumulative incidence of pulmonary TB than the non-depression group (1.68 vs. 1.50% at the end of follow-up P<0.001)	Incidence rate (per 10^5 py) = 93.3 in schizophrenics, 63.5 in controls, and 78.7 overall<br>Crude HR of TB in schizophrenics = 1.48 (95% CI 1.26-1.74, p<0.001) Adjusted HR = 1.52 (95% CI 1.29-1.79, p<0.001)<br>Kaplan-Meier analysis revealed a higher rate of newly-diagnosed TB in schizophrenics (log-rank test P<0.001)	77 patients had pulmonary TB, 3 had vertebracaries, and 1 had TB peritonitis<br>Relative risk observed vs. expected was 3.04 (P<0.005)<br>48% of the patients were diagnosed as having TB before being diagnosed as schizophrenic	Statistically significant association between overall CMDs and TB (OR: 1.34, 95% CI 1.06-1.68), which remains when adjusting for confounders (OR: 1.34, 95% CI 1.05-1.70)

PART 1	Oh et al 2017	Cheng et al 2017	Kuo et al 2013	Ohta et al 1988	de Araújo et al 2014
		Adjusted HR for pulmonary TB was 1.15 for depression group vs. non-depression group (95% CI 1.03, 1.28) (Cox)	Most of the diseases were pulmonary (87.7% schizophrenics and 85.5% controls)		
Subgroup analysis	IRR of depression to non-depression in patients with TB = 1.40 (95% CI 0.97-2.03) in men vs. 0.98 (95% CI 0.71-1.35) in women<br>IRR of depression to non-depression in patients with TB = 1.49 (95% CI 0.21-10.8) in DM vs. 1.17 (95% CI 0.91-1.51) in no DM<br>HR of mild depression 1.99 (95% CI 1.21-3.28, P=0.007) vs severe depression 3.08 (95% CI 2.00-4.73 P<0.0001) (Cox) – linear	Incidence of pulmonary TB, stratified by sex and age, also higher in the depression group than in controls<br>Adjusted HR of pulmonary TB was 1.37 for subjects with depression and without comorbidities compared to those without depression and without comorbidities (95% CI 1.17-1.62)	Compared with schizophrenia patients without TB, those with TB tended to have advanced age, male gender, higher Charlson's score, DM, myocardial infarction, and hypertension<br>Cox regression showed independent risks for newly-diagnosed TB to be age and male gender, while patients with hypertension were less likely to have new TB diseases	Observed number of male patients with TB was 48 (out of 1780), while expected number was 17.15 (RR=2.08, P<0.005)<br>Observed number of female patients with TB was 34 (out of 1471), while expected number was 9.78 (RR=3.48, P<0.005)<br>In schizophrenia patients born before 1925, the observed number with TB was 32 (out of 679), while expected number was 13.19 (RR=2.43, p<0.005), whereas in schizophrenia patients born after 1925, the observed number with TB was 50 (out of 2572), while expected number was 17.25 (RR=2.90, p<0.005)<br>Patients born after 1925 reached the greatest risk for the onset of schizophrenia around 1950, when antipsychotic drugs became widely available and outpatient treatment became more common, but relative risk of TB remained high despite these changes	9 of the 20 SRQ-20 symptoms were individually associated with TB (feeling sad recently, crying more than usual, frequent headaches, lack of appetite, feeling tired easily, difficulty feeling satisfied with tasks, feels constantly tired, feelings of uselessness, and loss of interest in things)
Statistical methods used	Kaplan-Meier to produce cumulative TB incidence curves<br>Log-rank test to analyse differences between the two cohorts<br>Incidence rate ratio of depression to non-depression and stratified<br>Multivariable Cox proportional hazards model – hazard ratio of depression on the development of TB, also by depression severity	Incidence of pulmonary TB estimated as number of pulmonary TB events identified during follow-up divided by total follow-up person-years for each group<br>Kaplan-Meier model for cumulative incidence of pulmonary TB in depression vs. control groups<br>Initially all variables included in a univariable model, and those found to be statistically significant included in multivariable Cox proportional hazards regression model to	Incidence of TB disease was compared by Poisson distribution, and the cumulative incidence of TB was compared by the Kaplan-Meier method (log-rank test)<br>Risk factors with p values <0.1 in univariate analysis (from chi-squared, independent t-tests, Mann-Whitney U tests as appropriate) entered into the multivariate analysis, and multivariable Cox proportional hazard regression was performed using backward elimination to analyse independent risk factors	Comparison of observed and expected number of schizophrenics with TB using relative risks – expected number calculated according to annual TB incidence of general population	Pearson's chi-square test<br>Association estimated with ORs and 95% CIs using a conditional logistic regression model with backward stepwise procedures, conditional analysis for individual matching, unconditional for frequency matching<br>Variables that were associated with the outcome in the univariable analysis with p<0.25 (diabetes, alcohol abuse, ethnicity, number of household goods, level of education, history of contact and crowding) and those that contributed to the

PART 1	Oh et al 2017	Cheng et al 2017	Kuo et al 2013	Ohta et al 1988	de Araújo et al 2014
		estimate hazard ratio and 95% CI for risk of pulmonary TB associated with depression	for TB diseases (P<0.05 considered significant)		model's goodness-of-fit or were *a priori* confounders (drug use) and the matching variables (age and sex) were included in the model
Reanalysis	Not required	Not required	Not required	Not possible	Not required
**Applicability**					
Have important populations been excluded from the study?	No: Exposure cohort derived from a nationwide population-based sample cohort	No: Exposure cohort derived from a nationwide population-based database	No: Nationwide-population based database (99% of Taiwanese population covered by national health insurance program)	No: Includes all those diagnosed with schizophrenia in Nagasaki	Yes: Excludes those who do not attend referral hospitals or community clinics e.g. those who are too depressed to seek treatment, and controls aren't representative of general population
Does the study directly address the review question?	Yes: Depression as a risk factor for TB	Yes: Depression as a risk factor for TB	Yes: Schizophrenia as a risk factor for TB	Yes: Association between schizophrenia and TB	Yes: Common mental disorders as a risk factor for TB, but hard to establish direction of association as case-control
**Other Information**					
Key conclusions of study authors	Patients with depression are at a higher risk for TB, and a dose-response relationship exists between depression and TB risk	Patients with depression are at a significantly higher risk of pulmonary TB than patients without depression	After adjusting for underlying diseases, schizophrenics had a higher incidence of newly-diagnosed TB	The incidence rate of TB was significantly higher than that of the general population for both male and female patients with schizophrenia	There appears to be appositive and independent association between common mental disorders and TB, but can't establish direction of causation
Correspondence with study authors	Email correspondence with Dr Oh to clarify why there are fewer person-years in the depression group than the controls: because the exposure cohort were followed up from the beginning of depression diagnosis (between 2003 and 2013) whereas the controls were followed up from the start of 2003	None	None	None	None

**PART 2**	**Koyanagi et al 2017**	**Hernández Sarmiento et al 2013**	**Lasebikan & Ige 2015**	**Srivastava et al 1983**	**de Castro-Silva et al 2019**
**General Information**					
Report title	Depression comorbid with tuberculosis and its impact on health status: cross-sectional analysis of community-based data from 48 low- and middle-income countries	Tuberculosis Among Homeless Population from Medellín, Columbia: Associated Mental Disorders and Socio-Demographic Characteristics	Prevalence of psychosis in tuberculosis patients and their nontuberculosis family contacts in a multidrug treatment-resistant treatment center in Nigeria	Psychological Aspects of Pulmonary Tuberculosis<br>Some information about the study taken from Gupta, L.N., Bhatia, B.L., Godara, R.C. et al. (1981). Life events, physical illness and psychiatric morbidity. Ind. J. Psychiat. 23, 338-342.	Prevalence of depression among patients with presumptive pulmonary tuberculosis in Rio de Janeiro, Brazil
Study funding source	ISCIII, ERDF-FEDER, National Institute of Health Research Collaboration for Leadership in Applied Health Research & Care Funding scheme	Not stated	Pan African Mental Health Initiative, Nigeria	Not stated	National Science and Technology Institute for Tuberculosis, the Graduate Program in Clinical Medicine of the Faculty of Medicine, Universidade Federal do Rio de Janerio, and the National Institute of Mental Health (USA)
Possible conflicts of interest	None declared	None declared	None declared	Not stated	None declared
**Study Characteristics**					
Type of study	Cross-sectional (population-based)	Cross-sectional (population-based)	Cross-sectional (comparing point prevalence in cases and controls)	Cross-sectional (comparing point prevalence in cases and controls)	Cross-sectional (comparing point prevalence in cases and controls)
Exposure	Depression	Mental disorders	Psychological distress/psychotic condition	Psychological state	Depression
Outcome	TB	Pulmonary TB	MDR-TB	Pulmonary TB	Pulmonary TB
**Population and Setting**					
Population description	General population (community-based, data nationally representative for all countries except 6)	Homeless people in Medellín, Columbia	Catchment area of clinic in Ibadan, Nigeria	Population of NW districts of Rajasthan and adjoining districts of Punjab and Haryana states	Patients with presumptive pulmonary TB in Rio de Janerio, Brazil
Setting	48 LMICs	Local health facility (Centro Día Uno) in Medellín	MDR-TB treatment center under the chest unit of the Department of Medicine, University College Hospital, Ibadan	Hospital for TB and chest diseases, Bikaner	Municipal Health Center of Duque de Caxias, Rio de Janerio
Inclusion criteria	Eligible participants in the WHS were those with a valid home address and aged ≥ 18 years; one individual was randomly chosen from the household with the use of Kish tables	Homeless adults (age >18 years) who attended the facility	Consenting patients with MDR-TB (pulmonary) served as cases, and the consenting accompanying family members or caregivers served as controls	Patients with pulmonary TB and controls	Patients aged 18 and over with presumptive pulmonary TB (cough lasting 3 weeks or longer)
Exclusion criteria	Countries without full data or those that are not LMICs were deleted, leaving 48 countries	None stated	Participants who were not literate in the languages of instruction (English and Yoruba), nonconsenting patients and	Patients who had a previous admission to a TB hospital<br>Patients who were more than 50 years of age	Individuals who had received anti-TB therapy for over 7 days, or who had taken a fluoroquinolone for more than 7

PART 2	Koyanagi et al 2017	Hernández Sarmiento et al 2013	Lasebikan & Ige 2015	Srivastava et al 1983	de Castro-Silva et al 2019
			nonconsenting family members or caregivers<br>Family members or caregivers with a past or current history of TB or any psychiatric disorder<br>All participants with any affective disorder, affective psychosis or delirium	Patients whose general condition was considered to be poor by the treating physician<br>Patients who were taking anti-TB drugs at the time of assessment or during the preceding one month<br>Patients whose duration of illness was more than two years<br>Patients who were suffering from high grade fever at the time of assessment<br>Patients who were having concomitant other physical illness<br>Patients who gave a history of previous psychotic illness or drug abuse	days in the preceding 30 days, as well as pregnant or lactating women and individuals for whom no final diagnosis of presence or absence of TB was established, were excluded
Method/s of recruitment	World Health Survey (WHS) – cross-sectional survey carried out in 70 countries from 2002 to 2004, involving single-stage random sampling (10 countries) or multi-stage random cluster sampling (60 countries), face-to-face interviews with trained interviewers	Asked to participate – non-probabilistic sampling	Both the cases and controls were consecutively recruited into the study	1-2 patients out of approximately 5 admissions, who met these screening criteria, were included each day in the study	Patients who presented to the Municipal Health Center with a complaint of cough lasting 3 weeks or longer were invited by a staff nurse to participate in the study
**Methods**					
Aim of study	To assess the association between TB and depression (and whether the co-occurrence of TB and depression confers a more pronounced decrement in health status and function compared to TB alone)	To assess TB incidence (actually prevalence?), its transmission patterns and association with socio-demographic factors and mental disorders in Colombian homeless people	To determine the prevalence of psychosis in TB patients in comparison to non-TB controls (and its correlation with disease pattern)	To discuss the psychological symptoms encountered in TB	To estimate the prevalence of current major depressive episode (MDE) among patients with presumptive pulmonary TB and compare it between patients with pulmonary TB and without pulmonary TB
Design	Cross-sectional survey	Cross-sectional survey (although authors claim that it is prospective)	Cross-sectional study (part of an ongoing prospective study)	Cross-sectional survey	Cross-sectional survey
Start date	2002	July 2006	January 2010	Not stated	July 2015
End date	2004	December 2007	November 2014	Not stated (but took place over 3 months)	December 2016
Duration of participation	N/A	N/A	N/A	N/A	N/A
**Participants**					
Total no.	242,952	426	227 (115 patients, 112 controls)	120 (60 patients, 60 controls)	260 (99 TB patients, 161 controls)

PART 2	Koyanagi et al 2017	Hernández Sarmiento et al 2013	Lasebikan & Ige 2015	Srivastava et al 1983	de Castro-Silva et al 2019
Baseline imbalances	Not stated	Not stated	Significant, see below	Not stated	The diagnosis of PTB was associated with a lower mean age, BMI < $18.5 kg/m^2$, cough duration > 8 weeks, and a lower frequency of hypertension
Withdrawals and exclusions	Overall response rate of WHS was 98.5%, and sampling weights generated to adjust for non-response	Not stated	Data were not complete for 3 controls so they were excluded (not counted as part of the 227 above)	Not stated	Of 3,251 presumptive pulmonary TB patients seen at the center during the study period, 341 (10.5%) were recruited<br>Of these, 81 (24%) were later excluded because they did not return with the results of sputum cultures<br>Among the 260 remaining subjects, 259 (99.6%) were screened for depression using the PHQ-9<br>Of those who screened positive, 97 (61%) were subsequently evaluated with the MINI-Plus
Age	Mean 38.4 (SD 16.1 years)	Mean 38.9 (SD 10.35 years)	Median age TB patients was 35 years but 42 years for controls	9 aged 15-24, 30 aged 25-34, 20 aged 35-40, and 11 aged 44+<br>(But this adds up to 70 rather than 60)	TB cases: mean 40.67 (SD 15.7 years)<br>Controls: mean 46.9 (SD 16.0 years)
Sex	Female predominant (50.8%)	Predominantly male (82.6%)	61.7% TB patients female but 82.1% controls female	40 male, 20 female	Male predominant (62.6% cases, 60.9% controls)
Severity of illness	6.9% depressive episode, 2.9% brief depressive episode, 2.6% subsyndromal depression	TB prevalence was 7.9%: of 426 enrolled, 183 had respiratory symptoms for PTB and 34 were diagnosed with PTB	36.5% mild TB, 52.2% moderate, 11.3% severe	Not stated	Cough duration > 8 weeks in 24.1% cases and 10.4% controls
Co-morbidities	3% diabetes	Not stated	Not stated	Not stated	24.2% cases had 1 or more comorbidities, vs. 19.9% controls
Treatment received	Not stated	Not stated	MDR-TB cases admitted to a 6-month intensive phase treatment followed by 12 months of ambulatory phase<br>Respondents who had diagnosable psychotic disorders were commenced on treatment	Not stated	All eligible patients were screened for PTB and treated at the facility as needed<br>All individuals with depression were referred for outpatient mental health treatment
Other relevant sociodemographics	E.g. mean household size 5.7 (SD 3.0), 56.5% rural, 26.5% no formal education	50.5% had been living on the streets 10 years or less, 54.5% had finished primary school	50.4% TB patients and 69.6% controls had no formal education	50 married, 8 unmarried, 2 widowed	Ethnicity – TB cases: 16.3% white, 36.3% black, 5.0% Asian, 40.0% mixed race, 2.5%

PART 2	Koyanagi et al 2017	Hernández Sarmiento et al 2013	Lasebikan & Ige 2015	Srivastava et al 1983	de Castro-Silva et al 2019
		education, 66.0% single, 55% had social relationships with other homeless people, main income sources commercial sale of goods (63%), sale of recycled goods (40%) and begging on the streets (37%)	34.8% TB patients and 53.6% controls married<br>34.8% TB patients and 49.1% controls employed<br>53.0% TB patients and 55.4% controls Islam (vs. Christianity)<br>66.1% TB patients and 66.1% controls Yoruba, 13.0% TB patients and 13.4% Hausa, 12.2% TB patients and 11.6% controls Igbo, 8.7% TB patients and 8.9% controls minority tribes	40 lower income, 16 middle income, 4 upper income<br>51 rural, 9 urban<br>51 Hindus, 9 Muslims	indigenous; controls: 20.0% white, 30.0% black, 9.2% Asian, 40.0% mixed race, 0.8% indigenous<br>Marital status – TB cases: 51.5% married; controls: 57.1% married<br>Education – TB cases: 21.0% education of at least 8 years; controls: 30.5%
Subgroups measured/reported	Region (Africa, Americas, Asia, Europe) and country income level (LICs vs. MICs)	None	Age, gender, education, marital status, employment status, religion, ethnicity, duration of illness, disease classification, disease extent, disease category	None	Sex, age, ethnicity, marital status, educational attainment, BMI, cough duration. family income, comorbidities, diabetes, drug use, hypertension, HIV status, smoking, alcoholism, homeless, contact with resident TB, incarcerated, contact with TB, living in shelter
**Exposure**					
No. in each group	205,752 (87.7%) no depression, 5238 (2.6%) subsyndromal depression, 6674 (2.9%) brief depressive episode, 13,965 (6.9%) depressive episode	77.2% drug dependent, 29.6% previous history of major depression, 62.0% suicide risk, 54.7% antisocial personality disorder, 48.8% alcohol dependence, 47.4% bipolar affective disorder, 15.7% dysthymia (persistent mild depression), 22.1% post-traumatic stress	41 with psychoses (38 in cases, 3 in controls), 186 without psychoses	60 TB patients and 60 controls	99 TB patients and 161 controls
Measurement	The severity of depressive symptoms was established based on the individual questions of the World Mental Health Survey version of the Composite International Diagnostic Interview, which assessed the duration and persistence of depressive symptoms in the past 12 months<br>Algorithms used to establish four mutually exclusive groups:	Mini-International Neuropsychiatric Interview (MINI), a simple, structured diagnostic interview that takes about 18 minutes and diagnoses mental disorders (including depression, suicide risk, PTSD, antisocial personality, dysthymia, bipolar affective disorder, drug and alcohol dependence)	All respondents were assessed for the presence of psychological distress and psychosis in the first 7 days of admission<br>Screened each patient and relative for psychiatric morbidity using General Health Questionnaire (GHQ-12), self-administered<br>All those who screened positive and a further 10% of those who screened negative were further	Interviewed with Present State Examination (PSE) for the assessment of psychiatric/psychological state – consists of a structured interview schedule that focuses on symptoms which have occurred during the preceding one month of interview	The Patient Health Questionnaire (PHQ-9) was used to screen all participants for symptoms of depression over the past 2 weeks, with a cut-off of greater than or equal to 10 points for moderate/severe depression (validated in Brazil)<br>The Mini International Neuropsychiatric Interview (MINI-Plus) major depressive disorder module was used to

**PART 2**	**Koyanagi et al 2017**	**Hernández Sarmiento et al 2013**	**Lasebikan & Ige 2015**	**Srivastava et al 1983**	**de Castro-Silva et al 2019**
	depressive episode, brief depressive episode, subsyndromal depression, and no depressive disorder		assessed using the Psychosis Screening Questionnaire<br>Psychosis module of the Structured Clinical Interview for DSM-IV Axis I Disorder was used to obtain a 12-month diagnosis of any nonaffective psychosis<br>Participants were periodically reviewed to detect new cases of psychosis while on admission in the MDR treatment facility, but this does not seem to be included in this study		confirm diagnosis of current major depressive episode in those diagnosed with depressive symptoms in the PHQ-9<br>These tools were administered by a nurse and 2 medical students
**Outcome**					
Time points measured/reported	2002-2004	July 2006-Dec 2007	Jan 2010-Nov 2014	Not stated	July 2015-Dec 2016
Outcome definition	Past 12-month symptoms of active TB, i.e. those who had both:<br>- a cough that lasted for 3 weeks or longer<br>- blood in phlegm (or coughed up blood)<br>These symptoms are likely to have a sensitivity of 65-70% and specificity of 55-75% in detecting TB	Medical screening examination to identify patients with respiratory symptoms compatible with pulmonary TB (cough and expectoration for 3 weeks or longer); from each subject who fulfilled the criteria, a sputum sample was taken for microscopic smear examination using conventional biochemical tests	Patients attending the MDR-TB treatment center<br>National Tuberculosis Program and WHO guidelines to assess the severity of TB, based on bacillary load, extent of disease and anatomical site that carries a significant acute threat to life and/or a risk of subsequent severe handicap	Pulmonary TB, no further details given	Pulmonary TB was diagnosed following the recommendations of the Brazilian national TB program for evaluation of patients with respiratory symptoms; patients with a positive sputum smear for acid-fast bacilli and/or a positive rapid molecular test for *M.tb* (Xpert MTB/RIF) were considered to be active TB cases; patients with negative smears and who ultimately received another diagnosis constituted the non-TB group
Person measuring/ reporting	Trained interviewers	Trained professionals (physicians, nurses and social workers)	Not stated	Not stated	Not stated
**Results**					
Comparison	Prevalence of depression in those with and without TB	With and without psychiatric disorders	Prevalence of psychoses in those with and without MDR-TB	Psychological symptoms in TB patients compared with controls	Depression and major depressive episode (MDE) in TB patients compared with controls
Outcome	Authors treat depression as outcome	Pulmonary TB diagnosis	Authors treat psychoses as outcome	Authors treat psychological symptoms as outcome	Authors treat depression and MDE as outcomes
Time point	2002-2004	July 2006-Dec 2007	Jan 2010-Nov 2014	Not stated	July 2015-Dec 2016
Results	TB cases: 23.7% of 3347<br>Controls: 6.8% of 196,417	Raw data not reported (see below for ORs)	MRD-TB cases: 38 per 115<br>Controls: 3 per 112	TB cases: 25 per 60<br>Controls: 8 per 60	In the screening test for depression, the proportion of TB and non-TB patients with a PHQ-9 score greater than or equal to 10

Hayward SE, *et al. BMJ Open* 2022; 12:e048945. doi: 10.1136/bmjopen-2021-048945

**PART 2**	**Koyanagi et al 2017**	**Hernández Sarmiento et al 2013**	**Lasebikan & Ige 2015**	**Srivastava et al 1983**	**de Castro-Silva et al 2019**
					was 60.2 vs. 62.1% (OR = 0.92, 95%CI 0.55-1.54, p = 0.79); among the patients with a PHQ-9 score greater than or equal to 10 who were subsequently assessed with the MINI-Plus, current MDE was identified in 59.5% of TB and 50.9% of non-TB patients, with no statistically significant difference (OR = 1.42, 95%CI 0.63-3.19, p = 0.42)
No. missing participants and reasons	17.7% data missing for TB, also over 10% missing data for BMI and diabetes, so missing values imputed for regression analyses	Not stated	Data were not complete for 3 controls so they were excluded	Not stated	Of 341 initially recruited, 81 (24%) were excluded because they did not return with the results of sputum cultures; of 260 remaining, 1 did not complete PHQ-9; of 159 who screened positive on PHQ-9, 62 were not evaluated with MINI-PLUS
Any other results reported	All types of depression were more frequent among those with TB, especially for depressive episode: prevalence of depressive episode was 23.7% (95% CI 20.5-27.1) with TB and 6.8% (95% CI 6.5-7.1) without TB (P<0.001) Multivariable multinomial logistic regression showed that TB is associated with a 1.98 (95% CI 1.47-2.67, P<0.0001), 1.75 (95% CI 1.26-2.42, P=0.0008) and 3.68 (95% CI 3.01-4.50, P<0.0001) times higher odds for subsyndromal depression, brief depressive episode, and depressive episode, respectively	Those with dysthymia had 2.66 times the odds (1.19-5.19, p=0.013) of pulmonary TB diagnosis and for those with previous history of major depression the OR was 2.22 (1.07-4.6, p=0.028)<br>In the multivariable model the adjusted OR for dysthymia was 2.54 (1.10-5.86, p=0.028)<br>Results for other mental disorders not reported: although an association between several mental disorders including major depression and having pulmonary TB was found by bivariate analysis, dysthymia was the only psychiatric disorder found to be associated with risk of having TB in the regression model analysis<br>13.4% reported having been previously diagnosed with TB, so perhaps issue of reverse causality<br>Incidence of TB in homeless population overall was 7.9% (34/426)	81.7% of TB patients scored positively on GHQ vs. 51.7% controls (P<0.001)<br>8.7% TB patients met DSM-IV criteria for schizophrenia vs. 0.9% controls<br>9.6% TB patients met DSM-IV criteria for other nonaffective psychosis vs. 1.8% controls<br>14.8% TB patients had anti-TB medication-induced psychotic disorder<br>33.0% TB patients had any psychosis vs. 2.7% controls	86.6% patients (52/60) had one or other psychiatric symptoms which could be rated as positive, compared with 70% controls (42/60)<br>41.6% patients (25/60) had symptoms in all six PSE subgroups, suggesting a diagnosable psychiatric illness, compared with 13.3% controls (8/60) (P<0.001)<br>Worries, tension headache, free floating anxiety, depressed mood, sleep and appetite disturbances, subjective anergia, retardation and irritability are the PSE symptoms revealed by significantly more TB patients compared with controls<br>None of the patients had psychotic symptoms (due to exclusion criteria?)<br>Other PSE symptoms that did not reach statistical significance were not reported	In both groups, the most frequently reported symptoms were depressed mood, sleep disturbance, fatigue, and appetite change. Individuals with PTB were more likely than those without PTB to endorse reduced appetite (87 vs. 73%, p = 0.009)<br><br>Loss to follow-up was higher among individuals with MDE (8.7%) compared to those without MDE (4.3%); however, this difference was not statistically significant. There were also no statistically significant associations of alcohol, drug abuse, or lower education with current MDE

**PART 2**	**Koyanagi et al 2017**	**Hernández Sarmiento et al 2013**	**Lasebikan & Ige 2015**	**Srivastava et al 1983**	**de Castro-Silva et al 2019**
				~50% patients reported being sad and anxious because of illness	
Subgroup analysis	TB was associated with a depressive episode across regions and country income levels although the estimates for Europe did not reach statistical significance, possibly due to lack of statistical power	None	Proportion of TB patients with positive GHQ and psychosis increased with increasing age, a higher proportion had a duration of illness ≥ 4 years, had extrapulmonary TB, had moderate or severe disease, and had category 2 TB	None	Women were two and a half times more likely to present with current MDE than men were (OR = 2.52, 95%CI, 1.10-5.76; p = 0.04); being female remained independently associated with MDE in logistic regression (OR = 2.72, 95%CI 1.39-5.30; p = 0.003)
Statistical methods used	Multivariable multinomial logistic regression analyses using the overall sample, and multivariable binary logistic regression while stratifying by region or country income level<br>All regression analyses adjusted for age, sex, education, wealth, household size, setting, current smoking, alcohol consumption, BMI, diabetes, and country<br>The sample weighting and complex study design were taken into account in all analyses	Bivariate analysis for the independent variables with their odds ratio and 95% CI for presence or absence of pulmonary TB<br>Multivariate analysis using an exploratory logistic regression with forward modelling was performed to determine the association between independent variables and pulmonary TB, using Hosmer-Lemeshow test to adjust the logistic regression model<br>Variables included in logistic regression model: income from drugs trade, social relationships with other homeless, receive food from other homeless, receive clothes from other homeless, live with homeless friends, stable relationships in couples, previous history of major depression, dysthymia	Chi-square test or Fisher's exact test for categorical variables<br>Mann-Whitney U test for nonparametric data<br>All chi-square p values were Bonferonni corrected where necessary	Chi-squared tests	Absolute and relative frequencies were calculated for categorical variables. Continuous variables are presented as mean and standard deviation. Associations of categorical variables with TB and MDE were evaluated using the chi-square test (or Fisher's exact test, when indicated); ORs were derived and 95% CIs calculated. Student's t-test was used to compare continuous variables. Logistic regression was used to control for variables independently associated with MDE. Statistical significance was accepted when p is greater than or equal to 0.05 (two-tailed)
Reanalysis	Not possible	Not required	Removing the 17 patients with medication-induced psychotic disorders, prevalence of psychosis in MDR-TB patients = 18.3% (21/115)	Not possible	Not required

PART 2	Koyanagi et al 2017	Hernández Sarmiento et al 2013	Lasebikan & Ige 2015	Srivastava et al 1983	de Castro-Silva et al 2019
**Applicability**					
Have important populations been excluded from the study?	No: Community-based survey (although excludes those without a valid home address, e.g. the institutionalised and the homeless who are at a high risk of TB)	Possibly: Only those homeless people attending one local health facility included	Yes: Only MDR-TB, and only one treatment centre<br>Are caregivers really representative controls?<br>Exclusion of participants with any affective disorder, affective psychosis or delirium could have distorted the prevalence<br>Also the exclusion of family members/caregivers with any psychiatric disorder	Yes: Extensive exclusion criteria with no justification	Possibly: Using patients of other respiratory illnesses as controls may not be representative
Does the study directly address the review question?	No: Treating depression as outcome and TB as exposure<br>Direction of causality cannot be established due to cross-sectional design	Yes: But direction of causality cannot be established due to cross-sectional design	No: Treating psychosis as outcome and MDR-TB as exposure<br>Direction of causality cannot be established due to cross-sectional design	No: Treating psychological state as outcome and TB as exposure<br>Direction of causality cannot be established due to cross-sectional design	Yes: But direction of causality cannot be established due to cross-sectional design
**Other Information**					
Key conclusions of study authors	TB is associated with the entire depression spectrum in the overall sample, and the association is comparable across regions and country income levels	Homeless people constitute a particular population with a high TB prevalence which co-exists with a high rate of mental disorders	Prevalence of psychosis among TB patients in this study is 33.0% (18.3% excluding medication-induced psychotic disorders compared to 2.7% among primary care givers	Significantly more patients as compared to control subjects are worried, anxious, depressed, irritable and have sleep and appetite disturbances	The prevalence of MDE (detected by the PHQ-9) among individuals with pre-PTB was high, and there was little difference between individuals with confirmed PTB and those with other respiratory illnesses (60.2 vs. 62.1%, respectively)
Correspondence with study authors	None	None	None	None	None
