# Supplementary material for: The relationship between mental health and risk of active tuberculosis: a systematic review
Source: BMJ Open. 2022 Jan 6;12(1):e048945. doi: 10.1136/bmjopen-2021-048945 (PMC8739435; doi:10.1136/bmjopen-2021-048945)
Supplement: Author's manuscript [file bmjopen-2021-048945.draft_revisions.pdf]

BMJ Open is committed to open peer review. As part of this commitment we make the peer review history of every article we publish publicly available.

When an article is published we post the peer reviewers' comments and the authors' responses online. We also post the versions of the paper that were used during peer review. These are the versions that the peer review comments apply to.

The versions of the paper that follow are the versions that were submitted during the peer review process. They are not the versions of record or the final published versions. They should not be cited or distributed as the published version of this manuscript.

BMJ Open is an open access journal and the full, final, typeset and author-corrected version of record of the manuscript is available on our site with no access controls, subscription charges or pay-per-view fees (http://bmjopen.bmj.com).

If you have any questions on BMJ Open's open peer review process please email info.bmjopen@bmj.com

BMJ Open

The relationship between mental health and risk of active tuberculosis: A systematic review

Journal:	*BMJ Open*
Manuscript ID	bmjopen-2021-048945
Article Type:	Original research
Date Submitted by the Author:	11-Jan-2021
Complete List of Authors:	Hayward, Sally; St George's University of London; London School of Hygiene & Tropical Medicine<br>Deal, Anna; St George's University of London; London School of Hygiene & Tropical Medicine<br>Rustage, Kieran; St George's University of London<br>Nellums, Laura ; University of Nottingham; St George's University of London<br>Sweetland, Annika; New York State Psychiatric Institute, Department of Psychiatry<br>Boccia, Delia; London School of Hygiene & Tropical Medicine<br>Hargreaves, Sally; St George's University of London<br>Friedland, Jon; St George's University of London
Keywords:	Tuberculosis < INFECTIOUS DISEASES, MENTAL HEALTH, Public health < INFECTIOUS DISEASES, Epidemiology < INFECTIOUS DISEASES

SCHOLARONE™
Manuscripts

I, the Submitting Author has the right to grant and does grant on behalf of all authors of the Work (as defined in the below author licence), an exclusive licence and/or a non-exclusive licence for contributions from authors who are: i) UK Crown employees; ii) where BMJ has agreed a CC-BY licence shall apply, and/or iii) in accordance with the terms applicable for US Federal Government officers or employees acting as part of their official duties; on a worldwide, perpetual, irrevocable, royalty-free basis to BMJ Publishing Group Ltd ("BMJ") its licensees and where the relevant Journal is co-owned by BMJ to the co-owners of the Journal, to publish the Work in this journal and any other BMJ products and to exploit all rights, as set out in our licence.

The Submitting Author accepts and understands that any supply made under these terms is made by BMJ to the Submitting Author unless you are acting as an employee on behalf of your employer or a postgraduate student of an affiliated institution which is paying any applicable article publishing charge ("APC") for Open Access articles. Where the Submitting Author wishes to make the Work available on an Open Access basis (and intends to pay the relevant APC), the terms of reuse of such Open Access shall be governed by a Creative Commons licence – details of these licences and which Creative Commons licence will apply to this Work are set out in our licence referred to above.

Other than as permitted in any relevant BMJ Author's Self Archiving Policies, I confirm this Work has not been accepted for publication elsewhere, is not being considered for publication elsewhere and does not duplicate material already published. I confirm all authors consent to publication of this Work and authorise the granting of this licence.

For peer review only

The relationship between mental health and risk of active tuberculosis: A systematic review

Sally E Hayward[1,2], Anna Deal[1,2], Kieran Rustage[1], Laura B Nellums[1,3], Annika C Sweetland[4], Delia Boccia[2*], Sally Hargreaves[1*], Jon S Friedland[1*]

[*]Joint senior authors

[1]Institute for Infection and Immunity, St George's, University of London, London, UK
[2]Faculty of Public Health and Policy, London School of Hygiene and Tropical Medicine, London, UK
[3]Division of Epidemiology and Public Health, School of Medicine, University of Nottingham, Nottingham, UK
[4]Department of Psychiatry, Columbia Vagelos College of Physicians and Surgeons, New York State Psychiatric Institute, New York, USA

Corresponding author:
Sally Hayward
Institute for Infection and Immunity
St George's, University of London
Cranmer Terrace, London
SW17 0RE, United Kingdom
Email: shayward@sgul.ac.uk

For peer review only

Abstract

Objectives. Tuberculosis (TB) and mental illnesses are highly prevalent globally and often co-exist. Whilst poor mental health is known to modulate immune function, whether mental disorders play a causal role in TB incidence is unknown. This systematic review examines the association between mental health and TB disease risk to inform clinical and public health measures.

Design. Systematic review, following PRISMA guidelines.

Search strategy and selection criteria. MEDLINE, PsycINFO and PsycEXTRA databases were searched alongside reference list and citation searching. Inclusion criteria were original research studies published 01/01/1970-11/05/2020 reporting data on the association between mental health and TB risk.

Data extraction, appraisal and synthesis. Data were extracted on study design and setting, sample characteristics, measurement of mental illness and TB, and outcomes including effect size or prevalence. Studies were critically appraised using CASP and AXIS checklists.

Results. 1546 records published over 50 years were screened, resulting in data synthesised from 607,184 individuals. Studies span across Asia, South America, and Africa, and include mood (e.g. depression) and psychotic (e.g. schizophrenia) disorders. There is robust evidence from cohort studies in Asia demonstrating that depression and schizophrenia can increase risk of active TB, with effect estimates ranging from HR=1.15 to 2.63 for depression and HR=1.52 to RR=3.04 for schizophrenia. This data in combination with evidence from cross-sectional studies supports an association between mental health and TB risk.

Conclusions. Individuals with mental illnesses including depression and schizophrenia experience increased TB incidence and represent a high-risk population to target for screening

For peer review only

and treatment. Integrated care for mental health and TB is needed, and interventions tackling mental illnesses and underlying drivers may help reduce TB incidence globally.

PROSPERO registration number. CRD42019158071

Strengths and limitations of this study

- We have undertaken a systematic and comprehensive examination of the evidence base on the relationship between mental health and tuberculosis (TB) incidence, following PRISMA guidelines
- This systematic review examines data from 607,184 individuals and finds evidence that mental health is a risk factor for active TB
- We identify those suffering from depression and schizophrenia as an at-risk population that could be identified and targeted for TB screening and treatment
- Studies addressing the association between mental health and TB are of variable design and quality, thus further cohort studies are needed to confirm the temporality of observed relationships across multiple mental illnesses and geographic locations

For peer review only

Introduction

Both tuberculosis (TB) and mental health are urgent global health priorities, with nearly 1.5 million TB deaths worldwide in 2018 [1], and approximately 14% of the global burden of disease attributable to neuropsychiatric disorders [2]. Mental disorders are defined here to include psychotic disorders (e.g. schizophrenia), mood or affective disorders (e.g. depression), and neurotic, stress-related and somatoform disorders (e.g. anxiety). There is increasing recognition that physical and mental health are interconnected [3, 4]. Mental illnesses are highly prevalent among TB patients and vice-versa [5, 6], and poor mental health is associated with reduced treatment-seeking and adherence, and therefore with greater morbidity, mortality, transmission, and drug resistance [7, 8]. Moreover, TB can infect the central nervous system (CNS) causing neurological symptoms [9], and certain anti-TB medications have psychiatric side effects [5]. The greatest burden of TB is experienced in individuals with risk factors including homelessness, drug and alcohol misuse, and migration [10]. Mental illness is more prevalent in all of these groups [11-13]. The relationships between TB and mental health are highly complex, with TB–depression comorbidity termed a 'syndemic' due to the bidirectional synergies involved [14].

Previous research has examined the impact of TB and its treatment on mental health [5], the relationship between mental disorders and TB treatment outcomes [7, 15] and adherence [8, 16], and the prevalence of mental disorders among TB and multidrug-resistant TB (MDR-TB) patients [17-19]. There is increasing interest in whether mental health may increase TB risk, supported by a growing body of evidence that chronic stressors and poor mental health directly influence the immune system, including susceptibility to infection [14, 20]. How these pathways operate in TB is unknown, although various mechanisms are possible; for

For peer review only - http://bmjopen.bmj.com/site/about/guidelines.xhtml

instance the suppression of cellular immunity due to poor mental health could contribute to reactivation of latent TB infection (LTBI) or progression from sub-clinical to clinical disease [21]. However, while the bi-directional relationship between mental health and TB has been the subject of discussion [6, 14, 21-25], there has been no systematic and comprehensive examination of the evidence base on associations between mental health and TB incidence. This systematic review aims to examine the evidence on the relationship between mental health and TB, to provide insight into whether poor mental health may be a risk factor for TB disease and to inform clinical and global public health measures.

Methods

Search strategy. We carried out a systematic review following PRISMA guidelines [26], registered with PROSPERO (CRD42019158071). MEDLINE, PsycINFO and PsycEXTRA were searched from inception to 11/5/2020, combining terms and subject headings for mental health and TB (Supplementary Box 1), restricting the search to English language papers. Records were imported into EndNote, and duplicates deleted. Two independent reviewers (SEH and KR/AD) carried out title/abstract and full-text screening using Rayyan QCRI [27]. We searched reference lists and carried out citation searching via Web of Science for included papers and previous reviews in this area [5, 7, 8, 15-19].

Selection criteria. This study includes empirical research reporting on the relationship between poor mental health and risk of active TB (Table 1). This includes two study designs: a) prospective studies investigating the causal relationship between mental health and TB incidence, and b) cross-sectional studies investigating the association between mental health

For peer review only - http://bmjopen.bmj.com/site/about/guidelines.xhtml

and TB disease. Cross-sectional studies were included regardless of whether they treated mental health as the exposure and TB as the outcome or vice versa, since both provide evidence for an association (provided that those with a history of mental illness are not excluded). Prevalence studies were excluded, as studies without controls cannot establish the presence of an association.

Only studies for which mental illness was a primary variable were included to ensure that the study was powered to detect an association. Studies solely investigating LTBI, CNS TB, or TB-HIV co-infection were excluded, as different mechanisms are expected to underlie the associations. Studies focused solely on substance use disorders were excluded as alcohol and drug use are already known to be strongly associated with TB risk, possibly due to social mixing patterns, or the effect of these substances on the immune system [28-30]. Studies relating to outcomes of TB infection or treatment (including psychiatric side effects of anti-TB medication), rather than incidence, were excluded, as this has been previously reviewed [7, 15].

Data extraction. Two independent reviewers (SEH and KR/AD) extracted data using a standardised form adapted from the Cochrane EPOC group [31], including study characteristics, population, setting, methods, participants, exposure, outcome, results (summary estimates), and applicability.

Critical appraisal. Two independent reviewers (SEH and KR/AD) assessed the quality of all included studies, using CASP checklists for cohort and case-control studies and the AXIS tool for cross-sectional studies [32, 33]. The tools were amended to remove questions on local applicability and implications for practice, instead asking about external validity and

For peer review only - http://bmjopen.bmj.com/site/about/guidelines.xhtml

implications for the review. A study was defined as high quality at a critical appraisal score of above 90%, moderate above 60%, and low at 60% or below. This score relates to the appropriateness and relevance of the study for this review question rather than in relation to the study's own aims.

Synthesis. Extracted data were tabulated, detailing methods and results of included studies, categorised by type of mental illness. Results are presented as reported in the studies. The primary outcome extracted is the main effect measure for the relationship between mental health and TB, and the secondary outcome is any additional measures reported relating to different definitions or levels of mental illness. For cross-sectional studies, any cases of mental illness arising post-TB diagnosis or treatment were excluded where possible. Given the wide range of populations, study designs, and statistical methods used, meta-analysis was not appropriate. A narrative synthesis was therefore completed, by study design and type of mental illness. Effect size and precision were compared where studies were sufficiently similar. The evidence as a whole was assessed as to whether poor mental health may be associated with, and act as a risk factor for, TB disease.

Patient and public involvement. Patients and/or the public were not involved in the design, conduct, reporting or dissemination plans of this research.

Results

We screened 1546 records published over 50 years and found that 10 published articles met the inclusion criteria (Figure 1), with a combined sample size of 607,184. These studies span

For peer review only - http://bmjopen.bmj.com/site/about/guidelines.xhtml

For peer review only

multiple locations, including five in Asia [34-38], three in South America [39-41], one in Africa [42], and one across low- and middle-income countries (LMICs) [43]. Seven studies assess mood disorders [34-36, 39-41, 43] and three assess psychotic disorders [37, 38, 42]. Seven studies investigate pulmonary TB exclusively [35, 36, 39-43], and three consider both pulmonary and extrapulmonary disease [34, 37, 38]. Study characteristics are summarised in Table 2. Only one paper included here was included in any prior systematic review of mental health and TB [5, 7, 8, 15-19].

Several study designs were used to investigate the association between mental health and TB. Four retrospective cohort studies use insurance or registry data to follow up TB incidence in those with a mental illness compared with matched controls [34, 35, 37] or general population incidence [38]. These are large studies with a combined sample size of 361,765 (Figure 2).

Other studies use questionnaire screening tools or interviews to assess mental health at a single timepoint. One case-control study identifies mental illnesses prior to TB diagnosis in cases and matched controls [40]. Two population-based cross-sectional studies establish prevalence of mental illnesses and TB, and assess the association between them [41, 43]. Three additional cross-sectional studies compare point prevalence of mental illnesses in TB cases and controls [36, 39, 42]. One of the population-based cross-sectional studies is very large with a sample of 242,952, whereas the other five cross-sectional studies are smaller with a combined sample size of 2,467 (Figure 2).

While the retrospective cohort studies use population-based databases or registers [34, 35, 37, 38], other studies are only applicable to certain populations such as the homeless [41] or

For peer review only

For peer review only - http://bmjopen.bmj.com/site/about/guidelines.xhtml

MDR-TB patients [42]. Results and study quality are tabulated in Table 3 and in detail in Supplementary Tables 1-2.

Mood disorders. Four studies specifically investigate depression [34, 35, 39, 43], and three look generally at mood disorders [36, 40, 41]. This includes two large, high-quality cohort studies on depression, both using nationally representative databases. One, in South Korea, reports an adjusted hazard ratio (HR) for TB incidence among those with depression compared with controls of 2.63 (95% confidence interval [CI] 1.74-3.96, p<0.001) [34], and the other, in Taiwan, reports an adjusted HR of 1.15 (95% CI 1.03-1.28) [35]. The former also finds a dose-response relationship, with more severe depression associated with greater TB risk. Both studies report a higher TB incidence rate in those with depression than in controls (Figure 3).

Other studies use a cross-sectional design to assess the association between mood disorders and TB. A large study using community-based World Health Survey data from across LMICs reports an adjusted odds ratio (OR) for a depressive episode in those with TB versus those without of 3.63 (95% CI 3.01-4.50) [43]. By contrast, a cross-sectional study among presumptive pulmonary TB cases in Rio de Janeiro, Brazil that compares the prevalence of depression among patients with and without a confirmatory diagnosis of pulmonary TB finds no evidence of a difference. Of 259 participants screened for depression, the prevalence among TB cases is 60.2% versus 62.1% in controls (crude OR=0.92, 95% CI 0.55-1.54, p=0.79), and of 159 who screened positive for depression, this diagnosis was confirmed in 59.5% of TB cases versus 50.9% of controls (crude OR=1.42, 95% CI 0.63-3.19, p=0.42) [39].

For peer review only - http://bmjopen.bmj.com/site/about/guidelines.xhtml

Beyond depression, a case-control study in Salvador, Brazil considers common mental disorders (CMDs) more broadly, characterised by diverse depressive, anxiety, or somatoform symptoms, and finds elevated odds of having TB among those with a CMD (adjusted OR=1.34, 95% CI 1.05-1.70) [40]. A cross-sectional study among the homeless in Medellín, Columbia investigates the association between TB and various psychiatric illnesses, but finds that only dysthymia (persistent mild depression) and history of major depression are associated with TB, and only for dysthymia is this statistically significant in multivariate analysis (adjusted OR=2.54, 95% CI 1.10-5.86, p=0.028) [41]. Finally, a cross-sectional study into the current psychological state of TB cases and controls in Bikaner, India finds a significant association between TB and symptoms including anxiety, depressed mood, and sleep/appetite disturbances, and reports a prevalence of psychiatric illness in TB patients of 41.6% versus 13.3% in controls [36].

Psychotic disorders. Two studies specifically investigate schizophrenia [37, 38], and one considers psychosis more generally [42]. The schizophrenia studies are retrospective cohort studies, both finding that schizophrenia is significantly associated with TB risk. A large, high quality study in Taiwan, using a nationwide database, reports an adjusted HR for TB incidence in those with schizophrenia versus controls of 1.52 (95% CI 1.29-1.79, p<0.001) [37]. The other, in Nagasaki, Japan uses registry data to identify TB incidence in those diagnosed with schizophrenia, and compares this to general population incidence. The reported relative risk (RR) for observed versus expected TB incidence is 3.04 (p<0.005) [38]. Both studies report a higher TB incidence rate in those with schizophrenia than in controls (Figure 3).

For peer review only

For peer review only - http://bmjopen.bmj.com/site/about/guidelines.xhtml

A cross-sectional study investigates psychosis more generally in MDR-TB patients in Ibadan, Nigeria. This reports a prevalence of psychosis of 33.0% in MDR-TB patients versus 2.7% in controls. When recalculated to exclude anti-TB medication-induced psychotic disorders, this is a prevalence of 18.3% in MDR-TB patients. This includes a prevalence of schizophrenia of 8.7% in TB patients vs. 0.9% in controls (χ^2=5.9, p=0.02) [42].

Discussion

Included studies, synthesising data from 607,184 individuals, show an association between mental health and TB. This includes robust evidence from cohort studies in Asia demonstrating that depression and schizophrenia increase risk of TB disease. While the co-existence of poor mental health and TB is consistent with existing literature [5], this is the first review to show that mental health is a risk factor for active TB.

Four population-based cohort studies are included, using registry or insurance records. Two cohort studies investigate how depression influences TB risk, reporting adjusted HRs of 2.63 in South Korea and 1.15 in Taiwan [34, 35]. The larger effect size in the former may be due to population differences, or perhaps because it adjusts for fewer comorbidities. The two cohort studies investigating the link between schizophrenia and incident TB also found significant associations, reporting an adjusted HR of 1.52 in Taiwan and a RR of 3.04 in Nagasaki, Japan [37, 38]. While derived from different statistical models so not directly comparable, the latter effect size may be larger because this study does not adjust for confounding.

For peer review only - http://bmjopen.bmj.com/site/about/guidelines.xhtml

Similarly, most cross-sectional or case control studies report an association between mental health and TB, although their design means that they cannot confirm the direction of any associations. While the cohort studies use official mental health diagnoses, other studies use screening tools and therefore can detect less severe and undiagnosed conditions. This includes a very large cross-sectional study using community-based survey data from across LMICs, which reports increased odds of a depressive episode in those with versus those without TB (OR=3.63) [43]. In the one study that does not find a significant association, controls are individuals with prolonged respiratory symptoms, who might be expected to have a higher prevalence of depression than the general population [39].

A key strength of this study is that, through undertaking an exhaustive search of the literature, it brings together a large sample of over 600,000 individuals. By synthesising evidence from a range of study designs, we collate all the evidence on this topic and analyse the findings holistically. Nevertheless, this review has certain limitations. Future reviews could focus on LTBI, CNS TB, and TB-HIV co-infection, which were excluded from this study. Moreover, the heterogeneity in study populations and methods made drawing direct comparisons between studies difficult and made meta-analysis inappropriate. For example, it was not possible to directly compare measures of effect generated from rates (HRs) with those generated from risks (RRs/ORs).

The nature of available evidence means that some caution is required when drawing conclusions. Studies addressing the association between mental health and TB are of variable design and quality, with four large studies (three cohort and one cross-sectional) accounting for 99% of included participants [34, 35, 37, 43]. Three included studies were classified as low quality; however, these only account for 0.001% of the total sample size (773

For peer review only

participants) [36, 41, 42]. Only the cohort studies can establish temporality to provide compelling evidence that mental health precedes and acts as a risk factor for TB, and these are the highest quality studies [34, 35, 37, 38]. However, the cohort studies only cover depression and schizophrenia and are all from Asia. Further cohort studies are required to confirm whether these findings hold true for other mental illnesses in other global regions.

The study findings increase our understanding of TB risk factors, yet further research is needed to elucidate the pathways by which mental health may increase TB incidence, causally or via associations between mental health and other risk factors for TB such as alcohol/drug use, homelessness, incarceration, physical co-morbidities, and poverty. The relationships between mental health, TB, and social risk factors are multi-directional, meaning that complex conceptual frameworks will be needed to understand the observed associations. The existence of plausible immune mechanisms supports a causal explanation, with evidence that psychosocial stressors are associated with immune biomarkers relevant to TB [44]. Mental illnesses including depression are associated with various immunological changes which could increase susceptibility to TB [14, 45]; however, the key neuroendocrine and immunological pathways involved are unknown. In addition, mental health could be one factor influencing risk of progression from LTBI to active disease. Understanding the pathways that connect mental health and the immune response to TB may guide the development of host-directed approaches, for example to prevent reactivation of LTBI.

The findings have implications for policy and clinical practice. The *Lancet* Commission on TB recommends that, to achieve a TB-free world, populations at high risk must be reached and brought into care [46]. The evidence presented here suggests that those suffering from mental illnesses, in particular depression and schizophrenia, could constitute such a high-risk

For peer review only - http://bmjopen.bmj.com/site/about/guidelines.xhtml

group for active case-finding and treatment. This group could benefit from a holistic approach, integrating services for mental health and TB to facilitate rapid diagnosis and treatment of TB disease and LTBI, as well as providing better mental health support for individuals with TB. As such, the World Health Organization End TB Strategy 2015-35 recommends that treatment for TB and mental health is brought together [47]. Providing high-quality mental health support and ensuring treatment adherence for TB both require substantial engagement with patients, so leveraging this contact to provide holistic care could prove effective [48].

In addition, the finding that mental illnesses constitute risk factors for TB suggests that tackling poor mental health and its underlying drivers may reduce TB incidence. In LMICs with a high TB incidence, poverty is consistently associated with common mental disorders [49]. Social protection schemes that lift individuals out of poverty are known to improve mental health and reduce TB risk factors [50-52], suggesting that tackling poverty and associated poor mental health through investment in wider social policies could help reduce TB incidence [53].

Conclusion

We find evidence that mental health is a risk factor for active TB. There is robust evidence from cohort studies based in Asia that depression and schizophrenia increase incidence of TB disease. This data, in combination with evidence from cross-sectional studies, identifies individuals with mental illnesses as a high-risk population for clinical TB that could be targeted for screening and treatment. This highlights the need for integrated programmes

For peer review only

For peer review only - http://bmjopen.bmj.com/site/about/guidelines.xhtml

273 providing care for mental health and TB and suggests that interventions that tackle mental

274 illnesses and their underlying drivers may help reduce TB incidence globally.

For peer review only

For peer review only - http://bmjopen.bmj.com/site/about/guidelines.xhtml

Contributors

SEH and DB developed the concept and study design. SEH, AD and KR carried out screening, data extraction, critical appraisal and analysis. All authors (SEH, AD, KR, LBN, ACS, DB, SH, JSF) contributed to critical review of data and manuscript writing.

Funding

SEH and AD are supported by Medical Research Council PhD studentships (MR/N013638/1), and KR receives funding from the Rosetrees Trust (M775). SH is supported by the National Institute for Health Research (NIHR Advanced Fellowship NIHR300072) and the Academy of Medical Sciences (SBF005\1111), and the European Society for Clinical Microbiology and Infectious Diseases (ESCMID) through an ESCMID Study Group for Infections in Travellers and Migrants (ESGITM)/ESCMID Study Group for Mycobacterial Infection (ESGMYC) research grant. LBN receives funding from the Academy of Medical Sciences (SBF005\1047), the Medical Research Council/Economic and Social Research Council/Arts and Humanities Research Council (MR/T046732/1), and the Medical Research Council (MR/V027549/1). ACS is supported by the US National Institute of Mental Health (K01 MH104514).

Competing interests

We declare no competing interests.

Data sharing statement

All data relevant to the study are included in the article or uploaded as supplementary information.

For peer review only

References

1. World Health Organization. Global tuberculosis report 2019. Geneva: WHO; 2019.

2. Mathers CD, Loncar D. Projections of Global Mortality and Burden of Disease from 2002 to 2030. PLOS Medicine. 2006;3(11):e442.

3. Prince M, Patel V, Saxena S, Maj M, Maselko J, Phillips MR, et al. No health without mental health. Lancet. 2007;370(9590):859-77.

4. Wang J, Wu X, Lai W, Long E, Zhang X, Li W, et al. Prevalence of depression and depressive symptoms among outpatients: a systematic review and meta-analysis. BMJ Open. 2017;7(8):e017173.

5. Doherty AM, Kelly J, McDonald C, O'Dywer AM, Keane J, Cooney J. A review of the interplay between tuberculosis and mental health. Gen Hosp Psychiatry. 2013;35(4):398-406.

6. Sweetland A, Oquendo M, Wickramaratne P, Weissman M, Wainberg M. Depression: a silent driver of the global tuberculosis epidemic. World Psychiatry. 2014;13(3):325-6.

7. Ruiz-Grosso P, Cachay R, de la Flor A, Schwalb A, Ugarte-Gil C. Association between tuberculosis and depression on negative outcomes of tuberculosis treatment: A systematic review and meta-analysis. PLoS ONE. 2020;15(1):e0227472.

8. Pachi A, Bratis D, Moussas G, Tselebis A. Psychiatric Morbidity and Other Factors Affecting Treatment Adherence in Pulmonary Tuberculosis Patients. Tuberculosis Research and Treatment. 2013;2013:489865.

9. Rock RB, Olin M, Baker CA, Molitor TW, Peterson PK. Central nervous system tuberculosis: pathogenesis and clinical aspects. Clinical microbiology reviews. 2008;21(2):243-61.

10. Public Health England. Tuberculosis in England: 2019 report. London: PHE; 2019.

For peer review only - http://bmjopen.bmj.com/site/about/guidelines.xhtml

11. Fazel S, Khosla V, Doll H, Geddes J. The Prevalence of Mental Disorders among the Homeless in Western Countries: Systematic Review and Meta-Regression Analysis. PLOS Medicine. 2008;5(12):e225.

12. Jané-Llopis E, Matytsina I. Mental health and alcohol, drugs and tobacco: a review of the comorbidity between mental disorders and the use of alcohol, tobacco and illicit drugs. Drug and Alcohol Review. 2006;25(6):515-36.

13. Priebe S, Giacco D, El-Nagib R. Public health aspects of mental health among migrants and refugees: a review of the evidence on mental health care for refugees, asylum seekers and irregular migrants in the WHO European Region (Health Evidence Network (HEN) Synthesis Report 47). Copenhagen: WHO Regional Office for Europe; 2016.

14. Sweetland AC, Kritski A, Oquendo MA, Sublette ME, Norcini Pala A, Silva LRB, et al. Addressing the tuberculosis-depression syndemic to end the tuberculosis epidemic. Int J Tuberc Lung Dis. 2017;21(8):852-61.

15. Lee GE, Scuffell J, Galea JT, Shin S, Magill E, Jaramillo E, et al. Impact of mental disorders on active TB treatment outcomes: a systematic review and meta-analysis. Int J Tuberc Lung Dis. 2020;24(12):1279-84.

16. Munro SA, Lewin SA, Smith HJ, Engel ME, Fretheim A, Volmink J. Patient Adherence to Tuberculosis Treatment: A Systematic Review of Qualitative Research. PLOS Medicine. 2007;4(7):e238.

17. Alene KA, Clements ACA, McBryde ES, Jaramillo E, Lonnroth K, Shaweno D, et al. Mental health disorders, social stressors, and health-related quality of life in patients with multidrug-resistant tuberculosis: A systematic review and meta-analysis. The Journal of infection. 2018;77(5):357-67.

For peer review only

For peer review only - http://bmjopen.bmj.com/site/about/guidelines.xhtml

18. Duko B, Bedaso A, Ayano G. The prevalence of depression among patients with tuberculosis: a systematic review and meta-analysis. Annals of general psychiatry. 2020;19:30.

19. Duko B, Dana LM, Ayano G. Psychological distress among TB patients in sub-Saharan Africa. Int J Tuberc Lung Dis. 2020;24(11):1200-4.

20. Glaser R, Kiecolt-Glaser JK. Stress-induced immune dysfunction: implications for health. Nat Rev Immunol. 2005;5(3):243-51.

21. Zhang K, Wang X, Tu J, Rong H, Werz O, Chen X. The interplay between depression and tuberculosis. Journal of leukocyte biology. 2019;106(3):749-57.

22. Mason PH, Sweetland AC, Fox GJ, Halovic S, Nguyen TA, Marks GB. Tuberculosis and mental health in the Asia-Pacific. Australasian Psychiatry. 2016;24(6):553-5.

23. Janse Van Rensburg A, Dube A, Curran R, Ambaw F, Murdoch J, Bachmann M, et al. Comorbidities between tuberculosis and common mental disorders: a scoping review of epidemiological patterns and person-centred care interventions from low-to-middle income and BRICS countries. Infect Dis Poverty. 2020;9(1):4.

24. Chandra M, Rana P, Chandra K, Arora VK. Tuberculosis - Depression syndemic: A public health challenge. The Indian journal of tuberculosis. 2019;66(1):197-202.

25. de Araújo GS, Pereira SM, dos Santos DN. Revisão sobre tuberculose e transtornos mentais comuns. Revista Eletrônica Gestão & Saúde. 2014;5(2):716-26.

26. Moher D, Liberati A, Tetzlaff J, Altman DG, The Prisma Group. Preferred Reporting Items for Systematic Reviews and Meta-Analyses: The PRISMA Statement. PLOS Medicine. 2009;6(7):e1000097.

27. Ouzzani M, Hammady H, Fedorowicz Z, Elmagarmid A. Rayyan—a web and mobile app for systematic reviews. Systematic Reviews. 2016;5(1):210.

For peer review only - http://bmjopen.bmj.com/site/about/guidelines.xhtml

28. Lönnroth K, Williams BG, Stadlin S, Jaramillo E, Dye C. Alcohol use as a risk factor for tuberculosis – a systematic review. BMC Public Health. 2008;8(1):289.

29. Imtiaz S, Shield KD, Roerecke M, Samokhvalov AV, Lönnroth K, Rehm J. Alcohol consumption as a risk factor for tuberculosis: meta-analyses and burden of disease. Eur Respir J. 2017;50(1):1700216.

30. Deiss RG, Rodwell TC, Garfein RS. Tuberculosis and illicit drug use: review and update. Clinical infectious diseases : an official publication of the Infectious Diseases Society of America. 2009;48(1):72-82.

31. Cochrane Effective Practice and Organisation of Care (EPOC). Data collection form. EPOC Resources for review authors. Available from: http://epoc.cochrane.org/epoc-specific-resources-review-authors [Accessed 28th March 2018]. 2017.

32. Critical Appraisal Skills Programme. CASP Checklists. Available from: https://casp-uk.net/casp-tools-checklists/ [Accessed 28th March 2018]. 2018.

33. EpiNet. AXIS - Appraisal Tool. Available from: http://www.epinet.net/AXIS-Appraisal-Tool [Accessed 25th March 2018].

34. Oh KH, Choi H, Kim EJ, Kim HJ, Cho SI. Depression and risk of tuberculosis: a nationwide population-based cohort study. Int J Tuberc Lung Dis. 2017;21(7):804-9.

35. Cheng KC, Liao KF, Lin CL, Lai SW. Increased Risk of Pulmonary Tuberculosis in Patients with Depression: A Cohort Study in Taiwan. Front Psychiatry. 2017;8:235.

36. Srivastava D, Gupta LN, Vyas JN. Psychological aspects of pulmonary tuberculosis. Indian Journal of Clinical Psychology. 1983;10(1):165-9.

37. Kuo SC, Chen YT, Li SY, Lee YT, Yang AC, Chen TL, et al. Incidence and outcome of newly-diagnosed tuberculosis in schizophrenics: a 12-year, nationwide, retrospective longitudinal study. BMC Infect Dis. 2013;13:351.

For peer review only - http://bmjopen.bmj.com/site/about/guidelines.xhtml

38. Ohta Y, Nakane Y, Mine M, Nakama I, Michitsuji S, Araki K, et al. The epidemiological study of physical morbidity in schizophrenics--2. Association between schizophrenia and incidence of tuberculosis. Jpn J Psychiatry Neurol. 1988;42(1):41-7.

39. Castro-Silva KM, Carvalho AC, Cavalcanti MT, Martins PDS, Franca JR, Oquendo M, et al. Prevalence of depression among patients with presumptive pulmonary tuberculosis in Rio de Janeiro, Brazil. Revista brasileira de psiquiatria (Sao Paulo, Brazil : 1999). 2019;41(4):316-23.

40. de Araújo GS, Pereira SM, dos Santos DN, Marinho JM, Rodrigues LC, Barreto ML. Common mental disorders associated with tuberculosis: a matched case-control study. PLoS ONE. 2014;9(6):e99551.

41. Hernandez Sarmiento JM, Correa N, Correa M, Franco JG, Alvarez M, Ramirez C, et al. Tuberculosis among homeless population from Medellin, Colombia: Associated mental disorders and socio-demographic characteristics. Journal of Immigrant and Minority Health. 2013;15(4):693-9.

42. Lasebikan VO, Ige OM. Prevalence of psychosis in tuberculosis patients and their nontuberculosis family contacts in a multidrug treatment-resistant treatment center in Nigeria. Gen Hosp Psychiatry. 2015;37(6):542-7.

43. Koyanagi A, Vancampfort D, Carvalho AF, DeVylder JE, Haro JM, Pizzol D, et al. Depression comorbid with tuberculosis and its impact on health status: cross-sectional analysis of community-based data from 48 low- and middle-income countries. BMC Med. 2017;15:209.

44. Hayward SE, Dowd JB, Fletcher H, Nellums LB, Wurie F, Boccia D. A systematic review of the impact of psychosocial factors on immunity: Implications for enhancing BCG response against tuberculosis. SSM - Population Health. 2020;10:100522.

For peer review only

45. Miller AH. Depression and immunity: a role for T cells? Brain Behav Immun. 2010;24(1):1-8.

46. Reid MJA, Arinaminpathy N, Bloom A, Bloom BR, Boehme C, Chaisson R, et al. Building a tuberculosis-free world: The Lancet Commission on tuberculosis. The Lancet. 2019.

47. World Health Organization. Implementing the End TB Strategy: the essentials. Geneva: WHO; 2015.

48. Sweetland AC, Galea J, Shin SS, Driver C, Dlodlo RA, Karpati A, et al. Integrating tuberculosis and mental health services: global receptivity of national tuberculosis program directors. Int J Tuberc Lung Dis. 2019;23(5):600-5.

49. Lund C, Breen A, Flisher AJ, Kakuma R, Corrigall J, Joska JA, et al. Poverty and common mental disorders in low and middle income countries: A systematic review. Soc Sci Med. 2010;71(3):517-28.

50. Ozer EJ, Fernald LCH, Weber A, Flynn EP, VanderWeele TJ. Does alleviating poverty affect mothers' depressive symptoms? A quasi-experimental investigation of Mexico's Oportunidades programme. International Journal of Epidemiology. 2011;40(6):1565-76.

51. Powell-Jackson T, Pereira SK, Dutt V, Tougher S, Haldar K, Kumar P. Cash transfers, maternal depression and emotional well-being: Quasi-experimental evidence from India's Janani Suraksha Yojana programme. Soc Sci Med. 2016;162:210-8.

52. Boccia D, Hargreaves J, Lonnroth K, Jaramillo E, Weiss J, Uplekar M, et al. Cash transfer and microfinance interventions for tuberculosis control: review of the impact evidence and policy implications. Int J Tuberc Lung Dis. 2011;15(Suppl 2):37-49.

For peer review only

For peer review only - http://bmjopen.bmj.com/site/about/guidelines.xhtml

53. Shete PB, Reid M, Goosby E. Message to world leaders: we cannot end tuberculosis without addressing the social and economic burden of the disease. The Lancet Global health. 2018;6(12):e1272-e3.

For peer review only

Figure captions

Figure 1: Study selection, PRISMA flow chart

PRISMA=Preferred Reporting Items for Systematic Reviews and Meta-Analyses

Figure 2: Sample size of included studies

Figure 3: TB incidence rate reported in longitudinal studies

TB=tuberculosis

For peer review only

Tables

Table 1: Inclusion criteria, using PECOS framework

	Inclusion criteria	Exclusion criteria
**Population**	Any population in any geographic location, including vulnerable groups e.g. the homeless	
**Exposure**	Mental illness, including psychotic and affective disorders, diagnosed clinically or by any psychological tool	- Alcohol and drug disorders<br>- Studies where mental illness is not a primary variable
**Control**	No mental illness	
**Outcome**	Incidence of active TB in humans, including reactivation of LTBI and MDR-TB	- LTBI without reactivation<br>- CNS TB (including tuberculosis meningitis)<br>- TB-HIV co-infection<br>- Studies investigating outcomes e.g. mortality (and not incidence)
**Study Design**	Observational epidemiological studies<br>a) Prospective studies (cohort): mental health must be treated as the exposure and TB as the outcome<br>b) Cross-sectional studies (cross-sectional and case-control): mental health may be treated as the exposure and TB as the outcome, or vice versa	- Prevalence studies without controls<br>- Qualitative studies<br>- Case reports<br>- Non-original research e.g. protocols, commentaries, reviews<br>- Prospective studies in which TB is the exposure and mental health is the outcome<br>- Cross-sectional studies that exclude those with a history of mental illness
**Dates**	Papers published 1970-2020	

CNS=central nervous system, HIV=human immunodeficiency virus, LTBI=latent tuberculosis infection, PECOS=population, exposure, control, outcome, study design, MDR-TB=multi-drug resistant tuberculosis, TB=tuberculosis

For peer review only

Table 2: Characteristics of all included studies

	Population		Study design				Exposure assessment		Outcome assessment		Controls	Critical Appraisal*
	Country	Gender & age	Design	Setting	Study period	*N*	Mental disorder	Measurement	Site of TB	Measurement		
**Mood disorders**												
Longitudinal studies												
**Oh et al 2017** [34]	South Korea	34% male; mean 45.8 years (SD 18.4)	Retrospective cohort study	Nationwide database	2003-13	64,744	Depression	ICD-10 codes plus psychotherapy prescription	Any	ICD-10 codes plus anti-TB drug prescription	No mood disorders, matched by age and sex	High (10/11, 91%)
**Cheng et al 2017** [35]	Taiwan	62% male; mean 47.9 years (SD 16.5) in cases and 47.6 (16.6) in controls	Retrospective cohort study	Nationwide database	2000-13	172,952	Depression	ICD-9 codes	Pulmonary	ICD-9 codes	No depression, matched for age, sex and comorbidities	High (10/11, 91%)
Cross-sectional studies												
**Koyanagi et al 2017** [43]	LMICs	49% male; mean 38.4 years (SD 16.1)	Cross-sectional study (population-based)	Community-based in 48 countries	2002-04	242,952	Depression	Interview (World Mental Health Survey version of the CIDI)	Pulmonary	TB symptoms in past 12 months (cough for three weeks or longer, blood in phlegm)	N/A	Moderate (17/20, 85%)
**De Castro-Silva et al 2019** [39]	Brazil	63% male in cases, 61% in controls; mean 40.7 years (SD 15.7) in cases, 46.9 (SD 16.0) in controls	Cross-sectional study (comparing point prevalence in cases and controls)	Municipal health centre in Rio de Janeiro	2015-16	260	Depression	Questionnaire (PHQ-9) and interview (MINI-Plus)	Pulmonary	Smear microscopy and/or Xpert MTB/RIF	Patients without TB, who were suspected to have pulmonary TB before testing	Moderate (13/20, 65%)
**De Araújo et al 2014** [40]	Brazil	61% male; mean 38.2 years (SD 14.2)	Case-control study	3 referral hospitals and 6 community clinics in Salvador	2008-10	1,434	Common Mental Disorders	Questionnaire (SRQ-20)	Pulmonary	Smear microscopy and culture for *M.tb*	Non-TB symptomatic respiratory patients, age- and sex-matched	Moderate (8/10, 80%)
**Hernández**	Columbia	83% male; mean 38.9	Cross-sectional	Homeless population	2006-07	426	Mental disorders	Interview (MINI)	Pulmonary	Smear microscopy	N/A	Low (12/20, 60%)

For peer review only

**Sarmiento et al 2013** [41]		years (SD 10.4)	study (population-based)	at a local health facility in Medellín								
**Srivastava et al 1983** [36]	India	67% male; age not available	Cross-sectional study (comparing point prevalence in cases and controls)	Hospital for TB and chest diseases, Bikaner	Not stated	120	Psychological state	Interview (PSE)	Pulmonary	Not specified	Non-TB patients at the hospital	Low (5/20, 25%)
**Psychotic disorders**												
Longitudinal studies												
**Kuo et al 2013** [37]	Taiwan	55% male; median 35.4 years in cases, 35.3 in controls	Retrospective cohort study	Nationwide database	1998-2009	120,818	Schizophrenia	ICD-9 codes	Any	ICD-9 codes plus anti-TB drug prescription	No schizophrenia, matched for age, sex, index date and comorbidities	High (10/11, 91%)
**Ohta et al 1988** [38]	Japan	55% male; age not available	Retrospective cohort study	Nationwide database	1960-78	3,251	Schizophrenia	Diagnosis (not further specified)	Any	Diagnosis (not further specified)	Expected incidence based on annual incidence rate	Moderate (7/11, 64%)
Cross-sectional study												
**Lasebikan & Ige 2015** [42]	Nigeria	38% male in cases, 18% in controls; median 35 years in cases, 42 in controls	Cross-sectional study (comparing point prevalence in cases and controls)	MDR-TB treatment centre, Ibadan	2010-14	227	Psychosis	Questionnaire (GHQ-12) and interview (psychosis screening questionnaire plus Structured Clinical Interview for DSM-IV Axis I Disorder, psychosis module)	Pulmonary	Attendance at MDR-TB treatment centre (not further specified)	Accompanying family members or caregivers	Low (11/20, 55%)

CIDI=Composite International Diagnostic Interview, CMD=common mental disorder, DSM=diagnostic and statistical manual, GHQ=general health questionnaire, ICD=international classification of diseases, LMIC=low- and middle-income country, *M.tb*/MTB=Mycobacterium tuberculosis, MDR=multi-drug resistant, MINI=Mini-International Neuropsychiatric Interview, N=sample size, PHQ=patient health questionnaire, PSE=present state interview, RIF-rifampicin, SD=standard deviation, SRQ=self-reporting questionnaire, TB=tuberculosis

*Critical appraisal score is defined based on number of positive responses to the questions on Critical Appraisal Skills Programme (CASP) checklists for cohort and case-control studies and the Appraisal Tool for Cross-Sectional Studies (AXIS) for cross-sectional studies

For peer review only - http://bmjopen.bmj.com/site/about/guidelines.xhtml

Table 3: Summary of study findings

	Primary outcome		Secondary outcomes		Statistical method	Adjustment
	**Effect measure**	**Results**	**Effect measure**	**Results**		
**Mood disorders**						
Longitudinal studies						
**Oh et al 2017** [34]	Adjusted HR for TB incidence in depressed vs. controls	2.63 (95% CI 1.74-3.96, p<0.001)	Adjusted HRs stratified by mild and severe depression	For mild depression = 1.99 (95% CI 1.21-3.28, p=0.007), for severe depression = 3.08 (95% CI 2.00-4.73 p<0.0001); linear dose-response	Cox proportional hazards model	Age, sex, income level, DM, COPD, alcoholism
**Cheng et al 2017** [35]	Adjusted HR for pulmonary TB incidence in depressed vs. controls	1.15 (95% CI 1.03-1.28)	-	-	Cox proportional hazards model	Age, sex, alcohol-related disease, CKD, chronic liver disease, COPD, DM, HIV infection, gastrectomy, and pneumoconiosis
Cross-sectional studies						
**Koyanagi et al 2017** [43]	Adjusted OR for depressive episode in those with pulmonary TB vs. those without	3.68 (95% CI 3.01-4.50, p<0.0001)	Adjusted ORs for brief depressive episode and for subsyndromal depression	For brief depressive episode = 1.75 (95% CI 1.26-2.42, p=0.0008), for subsyndromal depression = 1.98 (95% CI 1.47-2.67, p<0.0001)	Logistic regression	Age, sex, education, wealth, household size, setting, current smoking, alcohol consumption, BMI, diabetes, country
**De Castro-Silva et al 2019** [39]	Prevalence of depression in pulmonary TB patients; crude OR	Screening tool: 60.2% vs. 62.1% in controls (OR = 0.92, 95% CI 0.55-1.54, p=0.79) Diagnostic tool: 59.5% vs. 50.9% in controls (OR = 1.42, 95% CI 0.63-3.19, p=0.42)	-	-	Logistic regression	-
**De Araújo et al 2014** [40]	Adjusted OR for pulmonary TB in those with a CMD vs. those without	1.34 (95% CI 1.05-1.70)	-	-	Logistic regression	Diabetes, alcohol abuse, ethnicity, drug use, number of household goods, level of education, history of contact and crowding
**Hernández Sarmiento**	Crude and adjusted ORs for pulmonary	Dysthymia (adjusted) = 2.54 (95% CI 1.10-5.86,	-	-	Logistic regression	Age, gender, time living on the streets, education level,

For peer review only - http://bmjopen.bmj.com/site/about/guidelines.xhtml

**et al 2013** [41]	TB in those with mental disorders	p=0.028), dysthymia (crude) = 2.66 (95% CI 1.19-5.19, p=0.013), previous history of major depression (crude) = 2.22 (95% CI 1.07-4.6, p=0.028)				marital status, interaction with other homeless people, income sources
**Srivastava et al 1983** [36]	Prevalence of psychiatric illness in pulmonary TB patients vs. controls	41.6% vs. 13.3% in controls (p<0.001)	Prevalence of at least one psychiatric symptom in pulmonary TB patients	86.6% vs 70.0% in controls	Chi-square test	-
**Psychotic disorders**						
Longitudinal studies						
**Kuo et al 2013** [37]	Adjusted HR for TB incidence in those with schizophrenia vs. controls	1.52 (95% CI 1.29-1.79, p<0.001)	-	-	Cox proportional hazards model	Age, sex, Charlson's score, COPD, DM, rheumatoid disease, peptic ulcer disease, liver disease, hypertension, arrhythmia, dyslipidemia, drug or substance abuse, CKD, cancer, heart failure, peripheral vascular disease, myocardial infarction, hemiplegia/paraplegia, AIDS
**Ohta et al 1988** [38]	RR for TB incidence observed in those with schizophrenia vs. expected based on general population rates	3.04 (p<0.005)	-	-	Observed vs expected relative risk	Age, sex
Cross-sectional study						
**Lasebikan & Ige 2015** [42]	Prevalence of psychosis and schizophrenia in MDR-TB patients vs. controls	Psychosis: 33.0% (18.3% excluding medication-induced psychotic disorders‡) vs. 2.7% in controls	Prevalence of psychiatric morbidity in MDR-TB patients vs. controls	81.7% vs. 51.7% in controls (χ^2=21.7, p<0.001)	Chi-square test	-

For peer review only - http://bmjopen.bmj.com/site/about/guidelines.xhtml

		Schizophrenia: 8.7% vs. 0.9% in controls (χ^2=5.9, p=0.02)				

‡Recalculated to exclude mental health conditions that arise after TB diagnosis or treatment
AIDS=acquired immunodeficiency syndrome, BMI=body mass index, CI=confidence interval, CKD=chronic kidney disease, CMD=common mental disorder, COPD=chronic obstructive pulmonary disease, DM=diabetes mellitus, HIV=human immunodeficiency virus, HR=hazard ratio, MDR=multi-drug resistant, OR=odds ratio, RR=relative risk, SEP=socio-economic position, TB=tuberculosis

For peer review only

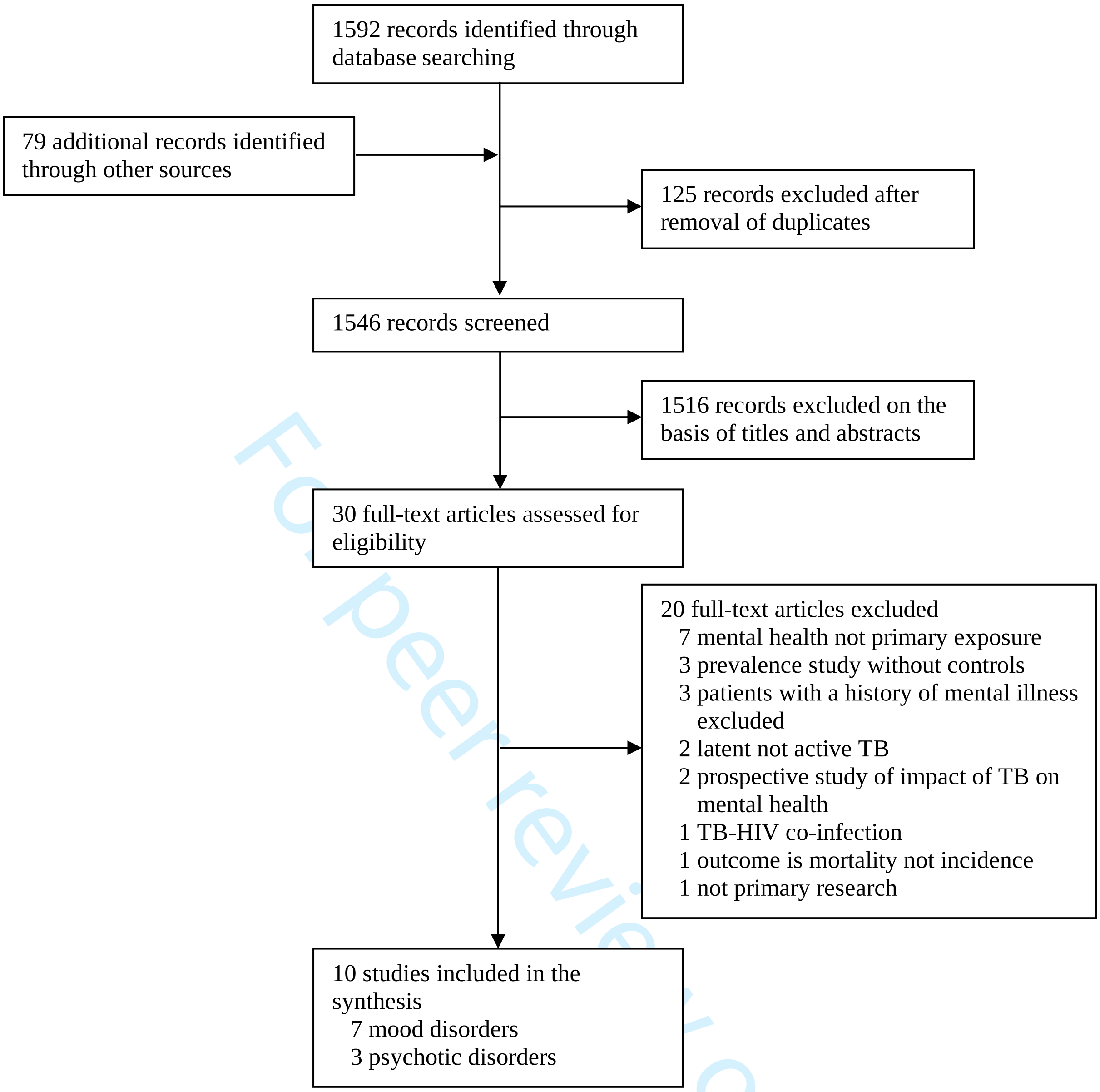

For peer review only

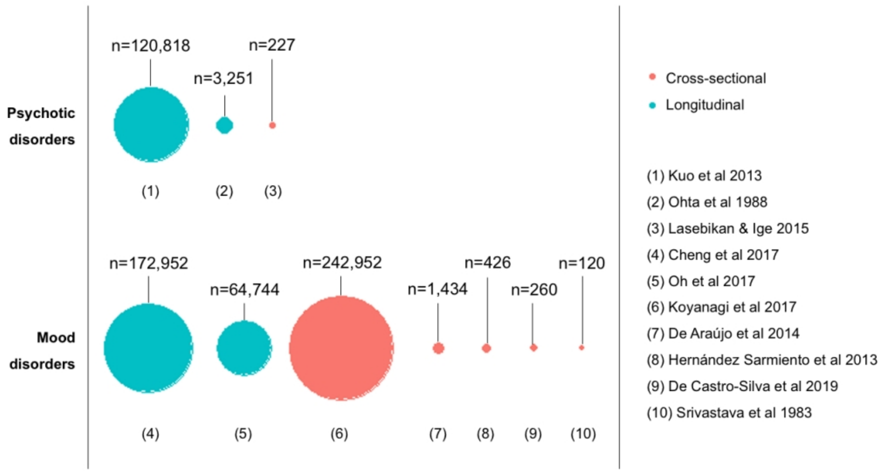

Figure 2: Sample size of included studies

338x190mm (108 x 108 DPI)

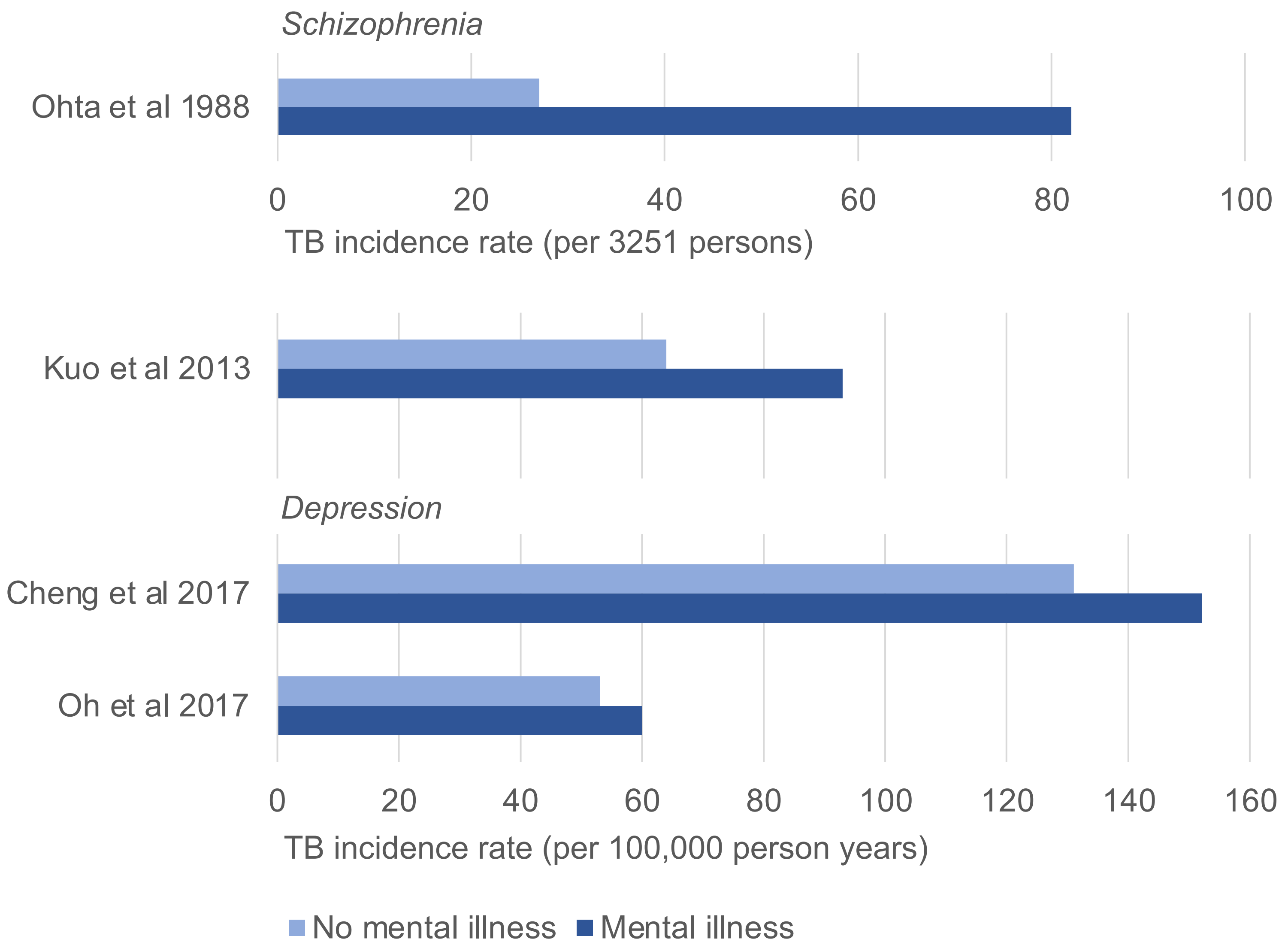
Schizophrenia
Ohta et al 1988
0
20
40
60
80
100
TB incidence rate (per 3251 persons)
Kuo et al 2013
Depression
Cheng et al 2017
Oh et al 2017
0
20
40
60
80
100
120
140
160
TB incidence rate (per 100,000 person years)
No mental illness
Mental illness

Supplementary Material

Supplementary Box 1: Search Strategy

MEDLINE Search Strategy:

1. ((Mental or Mentally or Psychological or Psychiatric) adj3 (health or illness* or disorder* or ill or unwell)).mp [mp=title, abstract, original title, name of substance word, subject heading word, keyword heading word, protocol supplementary concept word, rare disease supplementary concept word, unique identifier, synonyms]
2. exp Mental Health/
3. exp Mental Disorders/ep, et, mo, px, sn [Epidemiology, Etiology, Mortality, Psychology, Statistics & Numerical Data]
4. 1 or 2 or 3
5. (Tuberculosis or TB).mp. [mp=title, abstract, original title, name of substance word, subject heading word, keyword heading word, protocol supplementary concept word, rare disease supplementary concept word, unique identifier, synonyms]
6. exp Tuberculosis/ep, et, mo, px, sn, tm [Epidemiology, Etiology, Mortality, Psychology, Statistics & Numerical Data, Transmission]
7. 5 or 6
8. 4 and 7
9. limit 8 to English language

The PsycINFO search was the same as the MEDLINE search presented, except using subject headings for PsycINFO instead of MeSH terms. The PsycEXTRA search used the same free text terms, but this database does not use subject headings.

For peer review only - http://bmjopen.bmj.com/site/about/guidelines.xhtml

Supplementary Table 1: Critical appraisal

PART 1: Critical appraisal of cohort and case-control studies

**Cohort**	**Oh et al 2017**	**Cheng et al 2017**	**Kuo et al 2013**	**Ohta et al 1988**	**Case-control**	**de Araújo et al 2014**
**Are the results of the study valid?**						
1. Did the study address a clearly focused issue?	✓ To determine the association between depression and risk of TB	✓ To determine the association between depression and risk of pulmonary TB	✓ To determine the association between schizophrenia and risk (and outcome) of TB	✓ To investigate the incidence of TB among schizophrenics compared with the general population in Japan	1. Did the study address a clearly focused issue?	✓ To investigate the association between common mental disorders and risk of TB
2. Was the cohort recruited in an acceptable way?	✓ Nationwide population-based database, so less selection/recall bias and large sample size<br>Controls were more likely to have comorbidities than the general population as they were selected from a medical claims database – but comorbidities were adjusted for	✓ Nationwide population-based database, so less selection/recall bias and large sample size	✓ Nationwide population-based database, so less selection/recall bias and large sample size	✓ Registry of schizophrenics, so less selection/recall bias (but misses undiagnosed/ unregistered schizophrenics)	2. Did the authors use an appropriate method to answer their question?	✓ Cohort study would have provided stronger evidence for the directionality of association
3. Was the exposure accurately measured to minimise bias?	✓ ICD-10 codes for depression (requires psychiatrist's judgement from long-term observation), confirmed by prescription of psychotherapy – all psychiatrists in South Korea prescribe psychotherapy each time they see a patient under the fee-for-service system, so cases diagnosed as mild depression were also included	✓ ICD-9 codes for depression (but insurance claims not definitive measurement)	✓ ICD-9 code for schizophrenia, further validated if it was coded by psychiatrists (not clear whether this is a requirement for inclusion)	Can't tell – diagnostic criteria for schizophrenia not stated<br>Not clear whether authors are including incident or prevalent schizophrenia cases	3. Were the cases recruited in an acceptable way?	✓ Individuals diagnosed with pulmonary TB by attending chest physician at a health care unit in Salvador – excludes those who do not attend these clinics<br>Evidence of power calculation
4. Was the outcome accurately measured to minimise bias?	✓ ICD-10 codes for TB diagnosis, confirmed by prescription of two or more anti-TB drugs for simultaneous use for >30 days	✓ ICD-9 codes for pulmonary TB (but insurance claims not definitive measurement)	✓ ICD-9-CM codes for TB, plus prescription of at least two anti-TB drugs – but some bias may be introduced as any cases not prescribed anti-TB drugs will not be included	Can't tell – diagnostic criteria for TB not stated	4. Were the controls selected in an acceptable way?	× Symptomatic respiratory patients who were excluded from diagnosis of TB, age- and sex-matched – but are these patients representative of the general population?
5. a) Have the authors identified all important	× Age, gender, comorbidities (DM, COPD, alcoholism), income level	× Sex, age, comorbidities (comprehensive, but measured at baseline not during follow up)	× Age, gender, comorbidities (comprehensive)<br>But did not include any measure of SEP	× Only age and sex	5. Was the exposure accurately measured to minimise bias?	✓ Self-Reporting Questionnaire (SRQ-20), a well-recognised instrument, validated in Brazil

Cohort	Oh et al 2017	Cheng et al 2017	Kuo et al 2013	Ohta et al 1988	Case-control	de Araújo et al 2014
confounding factors?	Could have included more comorbidities	But did not include any measure of SEP				Instrument applied before diagnosis of TB
b) Have they taken account of the confounding factors in the design and/or analysis?	✓ Matched or included in multivariable Cox proportional hazards model	✓ Matched or included in multivariable Cox proportional hazards model	✓ Matched or included in multivariable Cox proportional hazards model	× Separate RRs calculated by sex and born before/after 1925, but no other factors considered and RR is not adjusted	6. a) What confounding factors have the authors accounted for?	✓ Marital status, ethnicity, history of contact with TB, diabetes, monthly family income, type of residence, number of household goods, level of education, crowding, recreational drugs, smoking, and alcohol consumption
6. a) Was the follow up of subjects complete enough?	✓ Nationwide database	✓ Nationwide database	✓ Nationwide database	✓ All TB cases must legally have been registered to public health centers in Nagasaki	b) Have they taken account of the potential confounding factors in the design and/or in their analysis?	✓ Matched or included in multivariable conditional logistic regression model
b) Was the follow up of subjects long enough?	✓ Followed up over up to 11 years	✓ Mean follow up 8.21 years depression and 8.30 years controls	✓ Median follow up 2,368 days for schizophrenics and 2,296 for controls	✓ (Up to?) 19 years		
**What are the results?**						
7. What are the results of this study?	Patients with depression are at a higher risk for TB (HR=2.63), with a dose-response relationship	Patients with depression are at a higher risk for TB (HR=1.15)	Schizophrenics are at a higher risk for TB (HR=1.52)	RR for TB incidence observed in those with schizophrenia vs. expected based on general population rates = 3.04	7. What are the results of this study?	Statistically significant association between overall CMDs and TB (adjusted OR=1.34)
8. How precise are the results?	HR=2.63 (95% CI 1.74-3.96)	HR=1.15 (95% CI 1.03-1.28)	HR=1.52 (95% CI 1.29-1.79, P<0.001)	No CIs given, P<0.005	8. How precise are the results? How precise is the estimate of risk?	Adjusted OR=1.34 (95% CI 1.05-1.70)<br>Overall P value not given
9. Do you believe the results?	✓ Big effect (CI does not cross 1), dose-response, study design seems sound	✓ Although effect isn't huge, CI is quite narrow and does not cross 1 (just), study design seems sound	✓ Although effect isn't huge, CI is reasonably narrow and does not cross 1, study design seems sound	✓ I believe that TB incidence is higher in schizophrenics than the general population (big effect), but to be able to say that there is an association would have been preferable to have controls and account for confounders Also would have been better to use rate per person-years	9. Do you believe the results?	✓ Although effect isn't huge, CI is quite narrow and does not cross 1 (just)
**What are the implications?**						
10. Do the results have good external validity?	✓ General population of South Korea likely to be largely representative of other HICs	✓ General population of Taiwan likely to be largely representative of other MICs/HICs	✓ General population of Taiwan likely to be largely representative of other MICs/HICs	✓ Population of Japan likely to be largely representative of other HICs	10. Do the results have good external validity?	× While the presence of an association is informative, the OR will only be relevant to this particular comparison with other respiratory patients
11. Do the results of this study fit with	✓ Depression as a risk factor for TB	✓ Depression as a risk factor for TB	✓ Schizophrenia as a risk factor for TB	✓ Schizophrenia as a risk factor for TB	11. Do the results of this study fit with	✓ Association between CMDs and TB

For peer review only

Cohort	Oh et al 2017	Cheng et al 2017	Kuo et al 2013	Ohta et al 1988	Case-control	de Araújo et al 2014
other available evidence?					other available evidence?	
12. What are the implications of this study for the review?	Supports conclusion that mental health is a risk factor for TB	Supports conclusion that mental health is a risk factor for TB	Supports conclusion that mental health is a risk factor for TB	Supports conclusion that mental health is a risk factor for TB	12. What are the implications of this study for the review?	Supports conclusion that mental health is associated with TB
**Overall judgement**						
Score	10/11 (91%)	10/11 (91%)	10/11 (91%)	7/11 (64%)	Score	8/10 (80%)
Overall study quality	High	High	High	Moderate	Overall study quality	Moderate

PART 2: Critical appraisal of cross-sectional studies

Cross-sectional	Koyanagi et al 2017	Hernández Sarmiento et al 2013	Lasebikan & Ige 2015	Srivastava et al 1983	de Castro-Silva et al 2019
**Introduction**					
1. Were the aims/ objectives of the study clear?	✓ To assess the association between depression and TB (and impact on health status) in LMICs	✓ To assess TB incidence, transmission patterns and association with socio-demographic factors and mental disorders in Colombian homeless people	✓ To determine the prevalence of psychosis in TB patients compared with controls (and its correlation with disease pattern)	✓ To discuss the psychological symptoms encountered in TB	✓ To estimate the prevalence of current major depressive episode (MDE) among patients with presumptive pulmonary TB (PTB) and compare it between patients with and without PTB
**Methods**					
2. Was the study design appropriate for the stated aim(s)?	✓ Cohort study would have provided evidence for the directionality of association	✓ Cohort study would have provided evidence for the directionality of association	✓ Cohort study would have provided evidence for the directionality of association	✓ Cohort study would have provided evidence for the directionality of association	✓ Cohort study would have provided evidence for the directionality of association
3. Was the sample size justified?	✓ Large sample (242,952), sample size determined by data available	× No justification given (relatively small sample size of 426)	× No justification given (small sample size of 227)	× No justification given (very small sample size of 120)	× No justification given (relatively small sample size of 260)
4. Was the target/reference population clearly defined? (Is it clear who the research was about?)	✓ General population of LMICs	✓ Homeless people in Medellín, Columbia	✓ Ibadan, Nigeria	✓ Population of NW districts of Rajasthan and adjoining districts of Punjab and Haryana states	✓ Presumptive pulmonary TB patients at a municipal health center in Rio de Janerio, Brazil
5. Was the sample frame taken from an appropriate population base so that it closely represented the target/reference population under investigation?	× Large sample size, predominantly nationally representative data (except 6 countries)<br>But eligible participants >18 years and have a valid home address – excludes those without a valid home address, e.g. homeless and institutionalised who are at a high risk of TB	× Homeless people who attended a local health entity in Medellín city, so excludes those who do not attend the facility	× Those attending the MDR-TB clinic may not be representative of all TB patients in Ibadan, and their family members/caregivers may not be representative of general population	× Hospital for TB and chest diseases, Bikaner, so excludes those who are undiagnosed or not admitted to this hospital	× Sample is presumptive pulmonary TB patients (presenting with a cough lasting 3 weeks or longer), so controls without TB may not be representative of general population
6. Was the selection process likely to select	✓ Single-stage random sampling (10 countries)	× Non-probabilistic sampling	× Exclusion criteria may introduce bias e.g. excluding	× Extensive exclusion criteria e.g. patients whose general condition	✓ Exclusion criteria are reasonable

For peer review only - http://bmjopen.bmj.com/site/about/guidelines.xhtml

Cross-sectional	Koyanagi et al 2017	Hernández Sarmiento et al 2013	Lasebikan & Ige 2015	Srivastava et al 1983	de Castro-Silva et al 2019
subjects/participants that were representative of the target/reference population under investigation?	Multi-stage random cluster sampling (60 countries)		those who were not literate in English or Yoruba and those with any affective disorder, affective psychosis or delirium, and excluding family members/caregivers with a past or current history of TB or any psychiatric disorder	was considered to be poor by the treating physician and patients who gave a history of previous psychotic illness or drug abuse	
7. Were measures undertaken to address and categorise non-responders?	✓ Sampling weights generated to adjust for non-response	× No information given on response rate or non-responders	× No information given on response rate or non-responders	× No information given on response rate or non-responders	× No data available on characteristics of non-responders
8. Were the risk factor and outcome variables measured appropriate to the aims of the study?	✓ Risk factor – depressive symptoms<br>Outcome – TB	✓ Risk factor – mental disorders<br>Outcome – pulmonary TB	✓ Risk factor – psychosis<br>Outcome – MDR-TB	✓ Risk factor – psychological state<br>Outcome – pulmonary TB	✓ Risk factor – depressive symptoms, major depressive episode<br>Outcome – pulmonary TB
9. Were the risk factor and outcome variables measured correctly using instruments/measurements that had been trialled, piloted or published previously?	× Risk factor – World Mental Health Survey version of the Composite International Diagnostic Interview (used previously)<br>Outcome – past 12-month symptoms of active TB (used previously, but sensitivity 65-70% and specificity 55-75% in detecting TB)	✓ Risk factor – Mini-International Neuropsychiatric Interview (pilot experiment)<br>Outcome – medical screening examination, sputum sample analysed in those with compatible symptoms	✓ Risk factor – GHQ-12 to screen for psychiatric morbidity (good internal consistency, validated and used in Nigeria), followed by psychosis screening questionnaire for those who screened positive (valid, used in Nigeria) and psychosis module of SCID<br>Outcome – patients attending the MDR-TB treatment center (culture and drug susceptibility test-proven cases)<br>Pilot study carried out on 15 patients and 15 relatives in a different hospital in Ibadan	✓ Risk factor – Present State Examination (PSE), no evidence of having been validated<br>Considering that if a patient has symptoms in all 6 PSE subgroups he/she must have a diagnosable psychiatric illness not justified<br>Symptoms included difficulty in breathing, weight loss – but surely this is more likely due to the TB?<br>Outcome – TB diagnostic criteria not stated	✓ Risk factor – PHQ-9 and MINI-Plus; both are recognised tools and PHQ-9 has been validated in Brazil; administered by trained interviewers<br>Outcome – pulmonary TB diagnosed following the recommendations of the Brazilian national TB program
10. Is it clear what was used to determined statistical significance and/or precision estimates? (e.g. p-values, confidence intervals)	✓ Level of statistical significance set at P<0.05<br>P values and 95% CIs reported	✓ Level of statistical significance set at P<0.05<br>P values and 95% CIs reported	✓ Level of statistical significance set at P<0.05<br>P values reported	× Not clear, but from table it seems that level of statistical significance was set at P<0.05	✓ Level of statistical significance set at P<0.05<br>P values and 95% CIs reported
11. Were the methods (including statistical methods) sufficiently described to enable them to be repeated?	✓ Detailed description of methods, with references to other publications with further details	× Methods of enrolment inadequately described (need for clarification of 'non-probabilistic sample')	× Methods somewhat unclear regarding patient recruitment (consecutive? random?), screening for psychosis (psychosis screening questionnaire vs. SCID), and statistical analysis (how were ORs calculated?)	× Methods of recruitment not clear (1-2 patients out of ~5 admissions per day, no mention of how controls were recruited), no information on TB diagnostic criteria, no explanation of statistical methods used	✓ Detailed description of methods

For peer review only - http://bmjopen.bmj.com/site/about/guidelines.xhtml

Cross-sectional	Koyanagi et al 2017	Hernández Sarmiento et al 2013	Lasebikan & Ige 2015	Srivastava et al 1983	de Castro-Silva et al 2019
			It is stated that participants were periodically reviewed to detect new cases of psychosis while on admission in the MDR treatment facility, but it is not clear how this is included in the analysis		
**Results**					
12. Were the basic data adequately described?	✓ Detailed table of sample characteristics	✓ Detailed table of socio-demographic variables	✓ Detailed table of socio-demographic characteristics of patients and family members/caregivers	× Only information on age and gender given	✓ Detailed tables of socio-demographic variables
13. Does the response rate raise concerns about non-response bias?	✓(No) Response rate 98.5% (but there is substantial missing data for some variables, >10% for TB, BMI and diabetes)	×(Yes) Response rate not stated	×(Yes) Response rate not stated	×(Yes) No information given on response rate	×(Yes) Of 3,251 presumptive PTB patients, only 260 were included in the sample
14. If appropriate, was information about non-responders described?	× But it is reported that sampling weights were generated using the population distribution to adjust for non-response, so this is not a big concern	× No information given on non-responders	× No information given on non-responders	× No information given on non-responders	× No data available on characteristics of non-responders
15. Were the results internally consistent?	✓ No apparent inconsistencies	✓ No apparent inconsistencies	✓ No apparent inconsistencies	× Number in each age group adds up to 70, when sample size is 60	× It is reported in the text that 98/260 participants were diagnosed with active PTB, but in Table 1 this is reported as 99/260
16. Were the results presented for all the analyses described in the methods?	✓ Yes	× In the bivariate and multivariate analysis only significant associations (between socio-demographics/psychiatric disorders and PTB) were reported	× It seems that only ORs that gave significant results were reported when looking at correlates of psychosis in TB patients	× Statistical methods were not described<br>Other PSE symptoms that did not reach statistical significance were not reported<br>Some findings seem to be alluded to in the discussion that were not reported in the results	× Sociodemographics only presented for factors that are associated with diagnosis of TB or current MDE, so some variables not reported e.g. family income
**Discussion**					
17. Were the authors' discussions and conclusions justified by the results?	✓ TB is associated with the entire depression spectrum in the overall sample, and the association is comparable across regions and country income levels<br>Lots of potential confounders included in analysis (although could have included more comorbidities)	✓ Homeless people are a population with a high TB prevalence which co-exists with a high rate of mental disorders<br>Seems a fair conclusion, and the authors also recognise that dysthymia is the only psychiatric disorder that remains associated with risk of TB when adjusting for confounders	× Prevalence of psychosis among TB patients in this study is 33.0% compared to 2.7% among primary care givers<br>Without any adjustment for confounding it is hard to draw any conclusions from this figure, especially considering substantial baseline imbalances	✓ Significantly more patients as compared to control subjects are worried, anxious, depressed, irritable and have sleep and appetite disturbances<br>While this statement is true in this study, it is not possible to say whether there is an association here without accounting for confounding	✓ The prevalence of MDE (detected by the PHQ-9) among individuals with pre-PTB was high, and there was little difference between individuals with confirmed PTB and those with other respiratory illnesses (60.2 vs. 62.1%, respectively)<br>However, as controls have other respiratory illnesses it is not

For peer review only - http://bmjopen.bmj.com/site/about/guidelines.xhtml

Cross-sectional	Koyanagi et al 2017	Hernández Sarmiento et al 2013	Lasebikan & Ige 2015	Srivastava et al 1983	de Castro-Silva et al 2019
		No adjustment for comorbidities is an issue<br>The significant results that arise may be due to multiple testing			possible to say the prevalence of depression is the same or different in TB patients compared with the general population
18. Were the limitations of the study discussed?	✓ Lacking information on HIV, based on TB symptoms not lab diagnosis, exclusion of institutionalised and homeless	✓ Difficulty differentiating primary psychotic and affective symptoms from secondary psychotic symptoms, only patients from one homeless shelter	✓ Small sample size, only those who scored positively on GHQ were assessed for psychosis, exclusion of patients with any affective disorder, affective psychosis or delirium, only one treatment centre included	× Limitations not discussed	✓ Loss of subjects who did not return for sputum culture, small sample size, inability to analyse causal relationships, MINI-Plus only used on subset of individuals
**Other**					
19. Were there any funding sources or conflicts of interest that may affect the authors' interpretation of the results?	✓(No) Funders had no role in study design, data collection, analysis and interpretation, or in writing manuscript	✓(No) None declared	✓(No) No conflict of interest declared (but perhaps there is one, since funder is Pan African Mental Health Initiative, Nigeria?)	×(Possibly) Not stated	✓(No) No conflict of interest declared
20. Was ethical approval or consent of participants attained?	✓ All participants gave informed consent	✓ All participants signed informed consent	✓ Only consenting participants included	× Not stated	✓ All participants signed informed consent
**Overall judgement**					
Score	17/20 (85%)	12/20 (60%)	11/20 (55%)	5/20 (25%)	13/20 (65%)
Overall study quality	Moderate	Low	Low	Low	Moderate

For peer review only - http://bmjopen.bmj.com/site/about/guidelines.xhtml

Supplementary Table 2: Details of Included Studies

**PART 1**	**Oh et al 2017**	**Cheng et al 2017**	**Kuo et al 2013**	**Ohta et al 1988**	**de Araújo et al 2014**
**General Information**					
Report title	Depression and risk of tuberculosis: a nationwide population-based cohort study	Increased Risk of Pulmonary Tuberculosis in Patients with Depression: A Cohort Study in Taiwan	Incidence and outcome of newly-diagnosed tuberculosis in schizophrenics: a 12-year, nationwide, retrospective longitudinal study	The Epidemiological Study of Physical Morbidity in Schizophrenics—2. Association between Schizophrenia and Incidence of Tuberculosis	Common Mental Disorders Associated with Tuberculosis: A Matched Case-Control Study
Study funding source	Not stated	Taiwan Ministry of Health and Welfare Clinical Trial Center, China Medical University Hospital, Academia Sinica Taiwan Biobank Stroke Biosignature Project, Taiwan Clinical Trial Consortium for Stroke, Tseng-Lien Lin Foundatin, Taichung, Taiwan, Taiwan Brain Disease Foundation, Taipei, Taiwan, and Katsuzo and Kiyo Aoshima Memorial Funds, Japan	National Health Research Institute, Taipei Veterans General Hospital, and the National Science Council	Not stated	National Council for Scientific and Technological Development
Possible conflicts of interest	None declared	Not stated	None declared	Not stated	None declared
**Study Characteristics**					
Type of study	Cohort, retrospective	Cohort, retrospective	Cohort, retrospective	Cohort, retrospective (longitudinal registry)	Matched case-control
Exposure	Depression	Depression	Schizophrenia	Schizophrenia	Common Mental Disorders
Outcome	TB incidence	Pulmonary TB incidence	TB incidence (and outcomes)	TB incidence	Pulmonary TB cases
**Population and Setting**					
Population description	South Korean population who made claims through the National Health Insurance Service	Taiwanese population covered by the National Health Insurance Program	Taiwanese population covered by National Health Insurance program	Those with schizophrenia living in Nagasaki, Japan	Symptomatic respiratory patients
Setting	Korean National Health Insurance Service-National Sample Cohort (NHIS-NSC), which sampled 1 million subjects with national representativeness from among the entire population covered by NHIS in 2002	Database of the Taiwan National Health Insurance Program	NHI Research Database (NHIRD), which is run by the National Health Research Institute	Nagasaki, Japan	Three referral hospitals and six community clinics in Salvador, Brazil
Inclusion criteria	Patients with depression newly diagnosed between 2003 and 2013 were selected from the NHIS-NSC to form the exposure cohort	Subjects aged 20-84 years with newly diagnosed depression from 2000 to 2012 selected into the depression group	Schizophrenic cohort comprised all patients who were admitted with schizophrenia from 1 Jan 1998 to 31 Dec 2009	All schizophrenic patients who resided in Nagasaki and were diagnosed as schizophrenic between 1960 and 1978	Individuals living in Salvador who were older than 14 years and who presented with respiratory symptoms and were investigated

For peer review only

PART 1	Oh et al 2017	Cheng et al 2017	Kuo et al 2013	Ohta et al 1988	de Araújo et al 2014
	The control cohort comprised the same number of individuals without any mood disorders, with each individual age- and sex-matched to a patient in the exposure cohort	Four subjects without depression randomly selected, for each subject with depression, into the non-depression group, matched for sex, age (every 5-year interval), and comorbidities	Controls randomly selected from the Longitudinal Health Insurance Database dataset, which contains complete data of 1,000,000 randomly sampled beneficiaries from the original NHIRD, to match schizophrenia patients on age (within 2 years), gender, index date, Charlson's score, chronic pulmonary disease, diabetes, and rheumatoid disease		for TB at any of several health care units
Exclusion criteria	Patients with a diagnosis of TB who used medical services in 2002 were excluded to rule out chronic conditions of depression or TB<br>Patients diagnosed with TB before the diagnosis of depression were excluded from the exposure cohort	Not stated	Schizophrenia group: patients who were diagnosed with TB before being admitted with schizophrenia were excluded<br>Control group: patients who were diagnosed with schizophrenia and antecedent TB were excluded	None stated	Those reporting a previous history of active TB
Method/s of recruitment	NHIS-NSC database	Database of the Taiwan National Health Insurance Program	Inpatient and outpatient claims data from NHIRD (National Health Insurance Research Database) in Taiwan	Registry of schizophrenic patients (from all psychiatric institutes in Nagasaki city and its suburbs)	All cases asked to participate, and the first eligible control who was an acceptable age and sex match was recruited into the study
**Methods**					
Aim of study	To determine the association between depression and risk of TB	To examine the causative relationship between depression and pulmonary TB in Taiwan	To compare the adjusted incidence and outcome of TB diseases in schizophrenics and the general population	To investigate the incidence of TB among schizophrenics	To investigate the association between common mental disorders and TB
Design	Retrospective cohort study using a nationwide database to identify incidence of TB in those with depression vs. controls	Retrospective cohort study using a nationwide database to identify incidence of TB in those with depression vs. controls	Retrospective cohort study using a nationwide database to identify incidence of TB in those with schizophrenia vs. controls	Registry data to identify TB incidence in those diagnosed with schizophrenia (1960-1978) compared with expected incidence based on annual incidence rate	Matched case-control study
Start date	2003	2000	January 1998	1960	August 2008
End date	2013	2013	December 2009	1978	April 2010
Duration of participation	Up to 11 years	All subjects followed up until they were diagnosed with pulmonary TB or to the end of 2013 – so up to 14 years, but mean follow-up is 8.21 years for depression and 8.30 for controls	From index date until end of December 2009, death, or diagnosis of TB (so up to 12 years) – median follow-up 2368 days (6.48 years)	(Up to?) 19 years	N/A
**Participants**					
Total no.	64,744	172,952	120,818	3251	1434 (717 cases, 717 controls)

PART 1	Oh et al 2017	Cheng et al 2017	Kuo et al 2013	Ohta et al 1988	de Araújo et al 2014
Baseline imbalances	See below	See below	Imbalances in some of the comorbidities that weren't matched e.g. drug or substance abuse 4.5% in schizophrenics vs. 0.02% in controls	Not stated	No differences in the matching variables, not stated for other variables
Withdrawals and exclusions	24 excluded as they were diagnosed with TB before the diagnosis of depression	Not stated	Not stated	Not stated	All individuals agreed to participate
Age	Mean 45.8 (SD 18.4 years)	Mean 47.9 (SD 16.5) for depression and 47.6 (16.6) for controls	Median 35.4 for schizophrenics and 35.3 for controls	More than 60% patients in their 20s to 40s when diagnosed with TB	Mean 38.2 (SD 14.2 years)
Sex	Female predominant (66%)	Male predominant (62.3%) in depression and controls	Male predominant (55.1%)	Male predominant (54.8%)	Male predominant, in cases (61.0%), and controls (60.5%)
Severity of illness	Ranging from mild to severe	Not stated	Not stated	Not stated	Not stated
Co-morbidities	0.6% DM in exposed vs. 22.6% in controls<br>0.1% COPD in exposed vs. 40.4% in controls<br>3.4% alcoholism in exposed vs. 0.6% in controls	Depression group had a higher proportion of asbestosis, CKD, HIV infection, gastrectomy, pneumoconiosis, and splenectomy than controls	Most (93.4% schizophrenics, 93.4% controls) did not have any severe comorbidities (Charlson's score < 3); most prevalent underlying diseases were peptic ulcer diseases (19.1% s, 18.0% c), chronic pulmonary disease (16.7% s, 16.7% c), and liver disease (12.9% s, 14.2% c)	Not stated	Not stated
Treatment received	Psychotherapy and anti-TB drugs (see below)	Not stated	Prescription of at least two anti-TB drugs (e.g., isoniazid, ethambutol, rifampin, pyrazinamide) for two months	Not stated	Not stated
Other relevant sociodemographics	22.4% low-income subjects in exposed vs. 25.1% in controls	Not stated	Not stated	Not stated	Not stated
Subgroups measured/reported	'Mild' and 'severe' depression	Sex, age (20-39, 40-64, 65-84), no comorbidity vs. any comorbidity	Age, gender, comorbidities (Charlson's score, diabetes, chronic pulmonary disease, rheumatoid disease, myocardial infarction, heart failure, peripheral vascular disease, peptic ulcer disease, liver disease, hemiplegia or paraplegia, chronic kidney disease, cancer, hypertension, dyslipidemia, arrhythmia, drug or substance abuse, and AIDS)	Sex, patients with schizophrenia born before and after 1925	Each of the dimensions of the SRQ-20
**Exposure**					
No. in each group	32,372 with depression, 32,372 controls	34,765 with depression, 138,187 controls	60,409 with schizophrenia, 60,409 controls	3251 with schizophrenia	513 with CMD (278 cases, 235 controls), 921 without CMD (439 cases, 482 controls)

For peer review only - http://bmjopen.bmj.com/site/about/guidelines.xhtml

PART 1	Oh et al 2017	Cheng et al 2017	Kuo et al 2013	Ohta et al 1988	de Araújo et al 2014
Measurement	ICD-10 codes for depression, confirmed by prescription of psychotherapy	ICD-9 codes for depression	Patients who were admitted with schizophrenia (ICD-9-CM code), validated if it was coded by psychiatrists	All patients who resided in the city and were diagnosed with schizophrenia between 1960 and 1978, using registry data from all psychiatric institutes in Nagasaki city and its suburbs	Self-Reporting Questionnaire (SRQ-20) to identify CMDs, individuals who scored above 7 were considered to have a CMD<br>Measured by a team of nursing technicians under the supervision of senior nurses
**Outcome**					
Time points measured/reported	2003-2013	2000-2013	1998-2009	1960-1978	Before the doctor diagnosed the presence or absence of TB
Outcome definition	ICD-10 codes for TB diagnosis, confirmed by prescription of two or more anti-TB drugs for simultaneous use for >30 days	New diagnosis of pulmonary TB during the follow-up period<br>ICD-9 codes for pulmonary TB	ICD-9-CM codes for TB, plus prescription of at least two anti-TB drugs for two months	All cases of TB (pulmonary and extrapulmonary) in the population have been registered at the public health centres in Nagasaki city according to certain legal procedures	Individuals diagnosed with pulmonary TB by smear microscopy and culture for *M. tb*<br>Controls selected from the symptomatic respiratory patients who were excluded from a diagnosis of TB
Person measuring/ reporting	Not stated	Not stated	Not stated	Not stated	Attending chest physician
**Results**					
Comparison	Depression vs. control cohort	Depression vs. control cohort	Schizophrenia vs. controls	Schizophrenia vs. general population	Those with CMD vs those without CMD
Outcome	TB incidence	Pulmonary TB incidence	New TB cases	TB incidence	Incident TB
Time point	Up to end of 2013	Up to end of 2013	Up to December 2009	1960-1978	Before the doctor diagnosed the presence or absence of TB
Results	Depression: 101 per 167,271 py (n=32,372)<br>No depression: 191 per 359,265 py (n=32,372)	Depression: 435 per 285,537 py (n=34,765)<br>No depression: 1504 per 1146,960 py (n=138,187)	Schizophrenia: 366 per 392,109 py (n=60409)<br>No schizophrenia: 241 per 379,548 py (n=60409)	Schizophrenia: 82 per 3251<br>Expected: 26.94 per 3251	CMDs: 278 per 513<br>No CMDs: 439 per 921
No. missing participants and reasons	Not stated	Not stated	Not stated	Not stated	Not stated
Any other results reported	TB incidence rate 60/100,000 py in depression cohort vs. 53/100,000 py in control cohort (P<0.0001 log-rank test)<br>Incidence rate ratio (IRR) of depression to non-depression in patients with TB = 1.14 (95% CI 0.89-1.45)<br>Risk of TB 2.63-fold (95% CI 1.74-3.96) higher in depression vs. control cohort (Cox)	Overall incidence of pulmonary TB was 1.16-fold greater in the depression group vs controls (1.52 vs. 1.31 per 1000 person years), i.e. IRR depression vs. non-depression = 1.16 (95% CI 1.12-1.21)<br>Kaplan-Meier model revealed that the depression group had a higher cumulative incidence of pulmonary TB than the non-depression group (1.68 vs. 1.50% at the end of follow-up P<0.001)	Incidence rate (per 10^5 py) = 93.3 in schizophrenics, 63.5 in controls, and 78.7 overall<br>Crude HR of TB in schizophrenics = 1.48 (95% CI 1.26-1.74, p<0.001) Adjusted HR = 1.52 (95% CI 1.29-1.79, p<0.001)<br>Kaplan-Meier analysis revealed a higher rate of newly-diagnosed TB in schizophrenics (log-rank test P<0.001)	77 patients had pulmonary TB, 3 had vertebracaries, and 1 had TB peritonitis<br>Relative risk observed vs. expected was 3.04 (P<0.005)<br>48% of the patients were diagnosed as having TB before being diagnosed as schizophrenic	Statistically significant association between overall CMDs and TB (OR: 1.34, 95% CI 1.06-1.68), which remains when adjusting for confounders (OR: 1.34, 95% CI 1.05-1.70)

For peer review only - http://bmjopen.bmj.com/site/about/guidelines.xhtml

PART 1	Oh et al 2017	Cheng et al 2017	Kuo et al 2013	Ohta et al 1988	de Araújo et al 2014
		Adjusted HR for pulmonary TB was 1.15 for depression group vs. non-depression group (95% CI 1.03, 1.28) (Cox)	Most of the diseases were pulmonary (87.7% schizophrenics and 85.5% controls)		
Subgroup analysis	IRR of depression to non-depression in patients with TB = 1.40 (95% CI 0.97-2.03) in men vs. 0.98 (95% CI 0.71-1.35) in women<br>IRR of depression to non-depression in patients with TB = 1.49 (95% CI 0.21-10.8) in DM vs. 1.17 (95% CI 0.91-1.51) in no DM<br>HR of mild depression 1.99 (95% CI 1.21-3.28, P=0.007) vs severe depression 3.08 (95% CI 2.00-4.73 P<0.0001) (Cox) – linear	Incidence of pulmonary TB, stratified by sex and age, also higher in the depression group than in controls<br>Adjusted HR of pulmonary TB was 1.37 for subjects with depression and without comorbidities compared to those without depression and without comorbidities (95% CI 1.17-1.62)	Compared with schizophrenia patients without TB, those with TB tended to have advanced age, male gender, higher Charlson's score, DM, myocardial infarction, and hypertension<br>Cox regression showed independent risks for newly-diagnosed TB to be age and male gender, while patients with hypertension were less likely to have new TB diseases	Observed number of male patients with TB was 48 (out of 1780), while expected number was 17.15 (RR=2.08, P<0.005)<br>Observed number of female patients with TB was 34 (out of 1471), while expected number was 9.78 (RR=3.48, P<0.005)<br>In schizophrenia patients born before 1925, the observed number with TB was 32 (out of 679), while expected number was 13.19 (RR=2.43, p<0.005), whereas in schizophrenia patients born after 1925, the observed number with TB was 50 (out of 2572), while expected number was 17.25 (RR=2.90, p<0.005)<br>Patients born after 1925 reached the greatest risk for the onset of schizophrenia around 1950, when antipsychotic drugs became widely available and outpatient treatment became more common, but relative risk of TB remained high despite these changes	9 of the 20 SRQ-20 symptoms were individually associated with TB (feeling sad recently, crying more than usual, frequent headaches, lack of appetite, feeling tired easily, difficulty feeling satisfied with tasks, feels constantly tired, feelings of uselessness, and loss of interest in things)
Statistical methods used	Kaplan-Meier to produce cumulative TB incidence curves<br>Log-rank test to analyse differences between the two cohorts<br>Incidence rate ratio of depression to non-depression and stratified<br>Multivariable Cox proportional hazards model – hazard ratio of depression on the development of TB, also by depression severity	Incidence of pulmonary TB estimated as number of pulmonary TB events identified during follow-up divided by total follow-up person-years for each group<br>Kaplan-Meier model for cumulative incidence of pulmonary TB in depression vs. control groups<br>Initially all variables included in a univariable model, and those found to be statistically significant included in multivariable Cox proportional hazards regression model to	Incidence of TB disease was compared by Poisson distribution, and the cumulative incidence of TB was compared by the Kaplan-Meier method (log-rank test)<br>Risk factors with p values <0.1 in univariate analysis (from chi-squared, independent t-tests, Mann-Whitney U tests as appropriate) entered into the multivariate analysis, and multivariable Cox proportional hazard regression was performed using backward elimination to analyse independent risk factors	Comparison of observed and expected number of schizophrenics with TB using relative risks – expected number calculated according to annual TB incidence of general population	Pearson's chi-square test<br>Association estimated with ORs and 95% CIs using a conditional logistic regression model with backward stepwise procedures, conditional analysis for individual matching, unconditional for frequency matching<br>Variables that were associated with the outcome in the univariable analysis with p<0.25 (diabetes, alcohol abuse, ethnicity, number of household goods, level of education, history of contact and crowding) and those that contributed to the

For peer review only - http://bmjopen.bmj.com/site/about/guidelines.xhtml

PART 1	Oh et al 2017	Cheng et al 2017	Kuo et al 2013	Ohta et al 1988	de Araújo et al 2014
		estimate hazard ratio and 95% CI for risk of pulmonary TB associated with depression	for TB diseases (P<0.05 considered significant)		model's goodness-of-fit or were *a priori* confounders (drug use) and the matching variables (age and sex) were included in the model
Reanalysis	Not required	Not required	Not required	Not possible	Not required
**Applicability**					
Have important populations been excluded from the study?	No: Exposure cohort derived from a nationwide population-based sample cohort	No: Exposure cohort derived from a nationwide population-based database	No: Nationwide-population based database (99% of Taiwanese population covered by national health insurance program)	No: Includes all those diagnosed with schizophrenia in Nagasaki	Yes: Excludes those who do not attend referral hospitals or community clinics e.g. those who are too depressed to seek treatment, and controls aren't representative of general population
Does the study directly address the review question?	Yes: Depression as a risk factor for TB	Yes: Depression as a risk factor for TB	Yes: Schizophrenia as a risk factor for TB	Yes: Association between schizophrenia and TB	Yes: Common mental disorders as a risk factor for TB, but hard to establish direction of association as case-control
**Other Information**					
Key conclusions of study authors	Patients with depression are at a higher risk for TB, and a dose-response relationship exists between depression and TB risk	Patients with depression are at a significantly higher risk of pulmonary TB than patients without depression	After adjusting for underlying diseases, schizophrenics had a higher incidence of newly-diagnosed TB	The incidence rate of TB was significantly higher than that of the general population for both male and female patients with schizophrenia	There appears to be appositive and independent association between common mental disorders and TB, but can't establish direction of causation
Correspondence with study authors	Email correspondence with Dr Oh to clarify why there are fewer person-years in the depression group than the controls: because the exposure cohort were followed up from the beginning of depression diagnosis (between 2003 and 2013) whereas the controls were followed up from the start of 2003	None	None	None	None

For peer review only - http://bmjopen.bmj.com/site/about/guidelines.xhtml

PART 2	Koyanagi et al 2017	Hernández Sarmiento et al 2013	Lasebikan & Ige 2015	Srivastava et al 1983	de Castro-Silva et al 2019
**General Information**					
Report title	Depression comorbid with tuberculosis and its impact on health status: cross-sectional analysis of community-based data from 48 low- and middle-income countries	Tuberculosis Among Homeless Population from Medellín, Columbia: Associated Mental Disorders and Socio-Demographic Characteristics	Prevalence of psychosis in tuberculosis patients and their nontuberculosis family contacts in a multidrug treatment-resistant treatment center in Nigeria	Psychological Aspects of Pulmonary Tuberculosis<br>Some information about the study taken from Gupta, L.N., Bhatia, B.L., Godara, R.C. et al. (1981). Life events, physical illness and psychiatric morbidity. Ind. J. Psychiat. 23, 338-342.	Prevalence of depression among patients with presumptive pulmonary tuberculosis in Rio de Janeiro, Brazil
Study funding source	ISCIII, ERDF-FEDER, National Institute of Health Research Collaboration for Leadership in Applied Health Research & Care Funding scheme	Not stated	Pan African Mental Health Initiative, Nigeria	Not stated	National Science and Technology Institute for Tuberculosis, the Graduate Program in Clinical Medicine of the Faculty of Medicine, Universidade Federal do Rio de Janerio, and the National Institute of Mental Health (USA)
Possible conflicts of interest	None declared	None declared	None declared	Not stated	None declared
**Study Characteristics**					
Type of study	Cross-sectional (population-based)	Cross-sectional (population-based)	Cross-sectional (comparing point prevalence in cases and controls)	Cross-sectional (comparing point prevalence in cases and controls)	Cross-sectional (comparing point prevalence in cases and controls)
Exposure	Depression	Mental disorders	Psychological distress/psychotic condition	Psychological state	Depression
Outcome	TB	Pulmonary TB	MDR-TB	Pulmonary TB	Pulmonary TB
**Population and Setting**					
Population description	General population (community-based, data nationally representative for all countries except 6)	Homeless people in Medellín, Columbia	Catchment area of clinic in Ibadan, Nigeria	Population of NW districts of Rajasthan and adjoining districts of Punjab and Haryana states	Patients with presumptive pulmonary TB in Rio de Janerio, Brazil
Setting	48 LMICs	Local health facility (Centro Día Uno) in Medellín	MDR-TB treatment center under the chest unit of the Department of Medicine, University College Hospital, Ibadan	Hospital for TB and chest diseases, Bikaner	Municipal Health Center of Duque de Caxias, Rio de Janerio
Inclusion criteria	Eligible participants in the WHS were those with a valid home address and aged ≥ 18 years; one individual was randomly chosen from the household with the use of Kish tables	Homeless adults (age >18 years) who attended the facility	Consenting patients with MDR-TB (pulmonary) served as cases, and the consenting accompanying family members or caregivers served as controls	Patients with pulmonary TB and controls	Patients aged 18 and over with presumptive pulmonary TB (cough lasting 3 weeks or longer)
Exclusion criteria	Countries without full data or those that are not LMICs were deleted, leaving 48 countries	None stated	Participants who were not literate in the languages of instruction (English and Yoruba), nonconsenting patients and	Patients who had a previous admission to a TB hospital<br>Patients who were more than 50 years of age	Individuals who had received anti-TB therapy for over 7 days, or who had taken a fluoroquinolone for more than 7

For peer review only - http://bmjopen.bmj.com/site/about/guidelines.xhtml

PART 2	Koyanagi et al 2017	Hernández Sarmiento et al 2013	Lasebikan & Ige 2015	Srivastava et al 1983	de Castro-Silva et al 2019
			nonconsenting family members or caregivers<br>Family members or caregivers with a past or current history of TB or any psychiatric disorder<br>All participants with any affective disorder, affective psychosis or delirium	Patients whose general condition was considered to be poor by the treating physician<br>Patients who were taking anti-TB drugs at the time of assessment or during the preceding one month<br>Patients whose duration of illness was more than two years<br>Patients who were suffering from high grade fever at the time of assessment<br>Patients who were having concomitant other physical illness<br>Patients who gave a history of previous psychotic illness or drug abuse	days in the preceding 30 days, as well as pregnant or lactating women and individuals for whom no final diagnosis of presence or absence of TB was established, were excluded
Method/s of recruitment	World Health Survey (WHS) – cross-sectional survey carried out in 70 countries from 2002 to 2004, involving single-stage random sampling (10 countries) or multi-stage random cluster sampling (60 countries), face-to-face interviews with trained interviewers	Asked to participate – non-probabilistic sampling	Both the cases and controls were consecutively recruited into the study	1-2 patients out of approximately 5 admissions, who met these screening criteria, were included each day in the study	Patients who presented to the Municipal Health Center with a complaint of cough lasting 3 weeks or longer were invited by a staff nurse to participate in the study
**Methods**					
Aim of study	To assess the association between TB and depression (and whether the co-occurrence of TB and depression confers a more pronounced decrement in health status and function compared to TB alone)	To assess TB incidence (actually prevalence?), its transmission patterns and association with socio-demographic factors and mental disorders in Colombian homeless people	To determine the prevalence of psychosis in TB patients in comparison to non-TB controls (and its correlation with disease pattern)	To discuss the psychological symptoms encountered in TB	To estimate the prevalence of current major depressive episode (MDE) among patients with presumptive pulmonary TB and compare it between patients with pulmonary TB and without pulmonary TB
Design	Cross-sectional survey	Cross-sectional survey (although authors claim that it is prospective)	Cross-sectional study (part of an ongoing prospective study)	Cross-sectional survey	Cross-sectional survey
Start date	2002	July 2006	January 2010	Not stated	July 2015
End date	2004	December 2007	November 2014	Not stated (but took place over 3 months)	December 2016
Duration of participation	N/A	N/A	N/A	N/A	N/A
**Participants**					
Total no.	242,952	426	227 (115 patients, 112 controls)	120 (60 patients, 60 controls)	260 (99 TB patients, 161 controls)

For peer review only

PART 2	Koyanagi et al 2017	Hernández Sarmiento et al 2013	Lasebikan & Ige 2015	Srivastava et al 1983	de Castro-Silva et al 2019
Baseline imbalances	Not stated	Not stated	Significant, see below	Not stated	The diagnosis of PTB was associated with a lower mean age, BMI < 18.5kg/m^2, cough duration > 8 weeks, and a lower frequency of hypertension
Withdrawals and exclusions	Overall response rate of WHS was 98.5%, and sampling weights generated to adjust for non-response	Not stated	Data were not complete for 3 controls so they were excluded (not counted as part of the 227 above)	Not stated	Of 3,251 presumptive pulmonary TB patients seen at the center during the study period, 341 (10.5%) were recruited<br>Of these, 81 (24%) were later excluded because they did not return with the results of sputum cultures<br>Among the 260 remaining subjects, 259 (99.6%) were screened for depression using the PHQ-9<br>Of those who screened positive, 97 (61%) were subsequently evaluated with the MINI-Plus
Age	Mean 38.4 (SD 16.1 years)	Mean 38.9 (SD 10.35 years)	Median age TB patients was 35 years but 42 years for controls	9 aged 15-24, 30 aged 25-34, 20 aged 35-40, and 11 aged 44+<br>(But this adds up to 70 rather than 60)	TB cases: mean 40.67 (SD 15.7 years)<br>Controls: mean 46.9 (SD 16.0 years)
Sex	Female predominant (50.8%)	Predominantly male (82.6%)	61.7% TB patients female but 82.1% controls female	40 male, 20 female	Male predominant (62.6% cases, 60.9% controls)
Severity of illness	6.9% depressive episode, 2.9% brief depressive episode, 2.6% subsyndromal depression	TB prevalence was 7.9%: of 426 enrolled, 183 had respiratory symptoms for PTB and 34 were diagnosed with PTB	36.5% mild TB, 52.2% moderate, 11.3% severe	Not stated	Cough duration > 8 weeks in 24.1% cases and 10.4% controls
Co-morbidities	3% diabetes	Not stated	Not stated	Not stated	24.2% cases had 1 or more comorbidities, vs. 19.9% controls
Treatment received	Not stated	Not stated	MDR-TB cases admitted to a 6-month intensive phase treatment followed by 12 months of ambulatory phase<br>Respondents who had diagnosable psychotic disorders were commenced on treatment	Not stated	All eligible patients were screened for PTB and treated at the facility as needed<br>All individuals with depression were referred for outpatient mental health treatment
Other relevant sociodemographics	E.g. mean household size 5.7 (SD 3.0), 56.5% rural, 26.5% no formal education	50.5% had been living on the streets 10 years or less, 54.5% had finished primary school	50.4% TB patients and 69.6% controls had no formal education	50 married, 8 unmarried, 2 widowed	Ethnicity – TB cases: 16.3% white, 36.3% black, 5.0% Asian, 40.0% mixed race, 2.5%

For peer review only

PART 2	Koyanagi et al 2017	Hernández Sarmiento et al 2013	Lasebikan & Ige 2015	Srivastava et al 1983	de Castro-Silva et al 2019
		education, 66.0% single, 55% had social relationships with other homeless people, main income sources commercial sale of goods (63%), sale of recycled goods (40%) and begging on the streets (37%)	34.8% TB patients and 53.6% controls married<br>34.8% TB patients and 49.1% controls employed<br>53.0% TB patients and 55.4% controls Islam (vs. Christianity)<br>66.1% TB patients and 66.1% controls Yoruba, 13.0% TB patients and 13.4% Hausa, 12.2% TB patients and 11.6% controls Igbo, 8.7% TB patients and 8.9% controls minority tribes	40 lower income, 16 middle income, 4 upper income<br>51 rural, 9 urban<br>51 Hindus, 9 Muslims	indigenous; controls: 20.0% white, 30.0% black, 9.2% Asian, 40.0% mixed race, 0.8% indigenous<br>Marital status – TB cases: 51.5% married; controls: 57.1% married<br>Education – TB cases: 21.0% education of at least 8 years; controls: 30.5%
Subgroups measured/reported	Region (Africa, Americas, Asia, Europe) and country income level (LICs vs. MICs)	None	Age, gender, education, marital status, employment status, religion, ethnicity, duration of illness, disease classification, disease extent, disease category	None	Sex, age, ethnicity, marital status, educational attainment, BMI, cough duration. family income, comorbidities, diabetes, drug use, hypertension, HIV status, smoking, alcoholism, homeless, contact with resident TB, incarcerated, contact with TB, living in shelter
**Exposure**					
No. in each group	205,752 (87.7%) no depression, 5238 (2.6%) subsyndromal depression, 6674 (2.9%) brief depressive episode, 13,965 (6.9%) depressive episode	77.2% drug dependent, 29.6% previous history of major depression, 62.0% suicide risk, 54.7% antisocial personality disorder, 48.8% alcohol dependence, 47.4% bipolar affective disorder, 15.7% dysthymia (persistent mild depression), 22.1% post-traumatic stress	41 with psychoses (38 in cases, 3 in controls), 186 without psychoses	60 TB patients and 60 controls	99 TB patients and 161 controls
Measurement	The severity of depressive symptoms was established based on the individual questions of the World Mental Health Survey version of the Composite International Diagnostic Interview, which assessed the duration and persistence of depressive symptoms in the past 12 months<br>Algorithms used to establish four mutually exclusive groups:	Mini-International Neuropsychiatric Interview (MINI), a simple, structured diagnostic interview that takes about 18 minutes and diagnoses mental disorders (including depression, suicide risk, PTSD, antisocial personality, dysthymia, bipolar affective disorder, drug and alcohol dependence)	All respondents were assessed for the presence of psychological distress and psychosis in the first 7 days of admission<br>Screened each patient and relative for psychiatric morbidity using General Health Questionnaire (GHQ-12), self-administered<br>All those who screened positive and a further 10% of those who screened negative were further	Interviewed with Present State Examination (PSE) for the assessment of psychiatric/psychological state – consists of a structured interview schedule that focuses on symptoms which have occurred during the preceding one month of interview	The Patient Health Questionnaire (PHQ-9) was used to screen all participants for symptoms of depression over the past 2 weeks, with a cut-off of greater than or equal to 10 points for moderate/severe depression (validated in Brazil)<br>The Mini International Neuropsychiatric Interview (MINI-Plus) major depressive disorder module was used to

For peer review only

PART 2	Koyanagi et al 2017	Hernández Sarmiento et al 2013	Lasebikan & Ige 2015	Srivastava et al 1983	de Castro-Silva et al 2019
	depressive episode, brief depressive episode, subsyndromal depression, and no depressive disorder		assessed using the Psychosis Screening Questionnaire<br>Psychosis module of the Structured Clinical Interview for DSM-IV Axis I Disorder was used to obtain a 12-month diagnosis of any nonaffective psychosis<br>Participants were periodically reviewed to detect new cases of psychosis while on admission in the MDR treatment facility, but this does not seem to be included in this study		confirm diagnosis of current major depressive episode in those diagnosed with depressive symptoms in the PHQ-9<br>These tools were administered by a nurse and 2 medical students
**Outcome**					
Time points measured/reported	2002-2004	July 2006-Dec 2007	Jan 2010-Nov 2014	Not stated	July 2015-Dec 2016
Outcome definition	Past 12-month symptoms of active TB, i.e. those who had both:<br>- a cough that lasted for 3 weeks or longer<br>- blood in phlegm (or coughed up blood)<br>These symptoms are likely to have a sensitivity of 65-70% and specificity of 55-75% in detecting TB	Medical screening examination to identify patients with respiratory symptoms compatible with pulmonary TB (cough and expectoration for 3 weeks or longer); from each subject who fulfilled the criteria, a sputum sample was taken for microscopic smear examination using conventional biochemical tests	Patients attending the MDR-TB treatment center<br>National Tuberculosis Program and WHO guidelines to assess the severity of TB, based on bacillary load, extent of disease and anatomical site that carries a significant acute threat to life and/or a risk of subsequent severe handicap	Pulmonary TB, no further details given	Pulmonary TB was diagnosed following the recommendations of the Brazilian national TB program for evaluation of patients with respiratory symptoms; patients with a positive sputum smear for acid-fast bacilli and/or a positive rapid molecular test for *M.tb* (Xpert MTB/RIF) were considered to be active TB cases; patients with negative smears and who ultimately received another diagnosis constituted the non-TB group
Person measuring/ reporting	Trained interviewers	Trained professionals (physicians, nurses and social workers)	Not stated	Not stated	Not stated
**Results**					
Comparison	Prevalence of depression in those with and without TB	With and without psychiatric disorders	Prevalence of psychoses in those with and without MDR-TB	Psychological symptoms in TB patients compared with controls	Depression and major depressive episode (MDE) in TB patients compared with controls
Outcome	Authors treat depression as outcome	Pulmonary TB diagnosis	Authors treat psychoses as outcome	Authors treat psychological symptoms as outcome	Authors treat depression and MDE as outcomes
Time point	2002-2004	July 2006-Dec 2007	Jan 2010-Nov 2014	Not stated	July 2015-Dec 2016
Results	TB cases: 23.7% of 3347<br>Controls: 6.8% of 196,417	Raw data not reported (see below for ORs)	MRD-TB cases: 38 per 115<br>Controls: 3 per 112	TB cases: 25 per 60<br>Controls: 8 per 60	In the screening test for depression, the proportion of TB and non-TB patients with a PHQ-9 score greater than or equal to 10

For peer review only - http://bmjopen.bmj.com/site/about/guidelines.xhtml

PART 2	Koyanagi et al 2017	Hernández Sarmiento et al 2013	Lasebikan & Ige 2015	Srivastava et al 1983	de Castro-Silva et al 2019
					was 60.2 vs. 62.1% (OR = 0.92, 95%CI 0.55-1.54, p = 0.79); among the patients with a PHQ-9 score greater than or equal to 10 who were subsequently assessed with the MINI-Plus, current MDE was identified in 59.5% of TB and 50.9% of non-TB patients, with no statistically significant difference (OR = 1.42, 95%CI 0.63-3.19, p = 0.42)
No. missing participants and reasons	17.7% data missing for TB, also over 10% missing data for BMI and diabetes, so missing values imputed for regression analyses	Not stated	Data were not complete for 3 controls so they were excluded	Not stated	Of 341 initially recruited, 81 (24%) were excluded because they did not return with the results of sputum cultures; of 260 remaining, 1 did not complete PHQ-9; of 159 who screened positive on PHQ-9, 62 were not evaluated with MINI-PLUS
Any other results reported	All types of depression were more frequent among those with TB, especially for depressive episode: prevalence of depressive episode was 23.7% (95% CI 20.5-27.1) with TB and 6.8% (95% CI 6.5-7.1) without TB (P<0.001) Multivariable multinomial logistic regression showed that TB is associated with a 1.98 (95% CI 1.47-2.67, P<0.0001), 1.75 (95% CI 1.26-2.42, P=0.0008) and 3.68 (95% CI 3.01-4.50, P<0.0001) times higher odds for subsyndromal depression, brief depressive episode, and depressive episode, respectively	Those with dysthymia had 2.66 times the odds (1.19-5.19, p=0.013) of pulmonary TB diagnosis and for those with previous history of major depression the OR was 2.22 (1.07-4.6, p=0.028)<br>In the multivariable model the adjusted OR for dysthymia was 2.54 (1.10-5.86, p=0.028)<br>Results for other mental disorders not reported: although an association between several mental disorders including major depression and having pulmonary TB was found by bivariate analysis, dysthymia was the only psychiatric disorder found to be associated with risk of having TB in the regression model analysis<br>13.4% reported having been previously diagnosed with TB, so perhaps issue of reverse causality<br>Incidence of TB in homeless population overall was 7.9% (34/426)	81.7% of TB patients scored positively on GHQ vs. 51.7% controls (P<0.001)<br>8.7% TB patients met DSM-IV criteria for schizophrenia vs. 0.9% controls<br>9.6% TB patients met DSM-IV criteria for other nonaffective psychosis vs. 1.8% controls<br>14.8% TB patients had anti-TB medication-induced psychotic disorder<br>33.0% TB patients had any psychosis vs. 2.7% controls	86.6% patients (52/60) had one or other psychiatric symptoms which could be rated as positive, compared with 70% controls (42/60)<br>41.6% patients (25/60) had symptoms in all six PSE subgroups, suggesting a diagnosable psychiatric illness, compared with 13.3% controls (8/60) (P<0.001)<br>Worries, tension headache, free floating anxiety, depressed mood, sleep and appetite disturbances, subjective anergia, retardation and irritability are the PSE symptoms revealed by significantly more TB patients compared with controls<br>None of the patients had psychotic symptoms (due to exclusion criteria?)<br>Other PSE symptoms that did not reach statistical significance were not reported	In both groups, the most frequently reported symptoms were depressed mood, sleep disturbance, fatigue, and appetite change. Individuals with PTB were more likely than those without PTB to endorse reduced appetite (87 vs. 73%, p = 0.009)<br><br>Loss to follow-up was higher among individuals with MDE (8.7%) compared to those without MDE (4.3%); however, this difference was not statistically significant. There were also no statistically significant associations of alcohol, drug abuse, or lower education with current MDE

For peer review only - http://bmjopen.bmj.com/site/about/guidelines.xhtml

**PART 2**	**Koyanagi et al 2017**	**Hernández Sarmiento et al 2013**	**Lasebikan & Ige 2015**	**Srivastava et al 1983**	**de Castro-Silva et al 2019**
				~50% patients reported being sad and anxious because of illness	
Subgroup analysis	TB was associated with a depressive episode across regions and country income levels although the estimates for Europe did not reach statistical significance, possibly due to lack of statistical power	None	Proportion of TB patients with positive GHQ and psychosis increased with increasing age, a higher proportion had a duration of illness ≥ 4 years, had extrapulmonary TB, had moderate or severe disease, and had category 2 TB	None	Women were two and a half times more likely to present with current MDE than men were (OR = 2.52, 95%CI, 1.10-5.76; p = 0.04); being female remained independently associated with MDE in logistic regression (OR = 2.72, 95%CI 1.39-5.30; p = 0.003)
Statistical methods used	Multivariable multinomial logistic regression analyses using the overall sample, and multivariable binary logistic regression while stratifying by region or country income level<br>All regression analyses adjusted for age, sex, education, wealth, household size, setting, current smoking, alcohol consumption, BMI, diabetes, and country<br>The sample weighting and complex study design were taken into account in all analyses	Bivariate analysis for the independent variables with their odds ratio and 95% CI for presence or absence of pulmonary TB<br>Multivariate analysis using an exploratory logistic regression with forward modelling was performed to determine the association between independent variables and pulmonary TB, using Hosmer-Lemeshow test to adjust the logistic regression model<br>Variables included in logistic regression model: income from drugs trade, social relationships with other homeless, receive food from other homeless, receive clothes from other homeless, live with homeless friends, stable relationships in couples, previous history of major depression, dysthymia	Chi-square test or Fisher's exact test for categorical variables<br>Mann-Whitney U test for nonparametric data<br>All chi-square p values were Bonferonni corrected where necessary	Chi-squared tests	Absolute and relative frequencies were calculated for categorical variables. Continuous variables are presented as mean and standard deviation. Associations of categorical variables with TB and MDE were evaluated using the chi-square test (or Fisher's exact test, when indicated); ORs were derived and 95% CIs calculated. Student's t-test was used to compare continuous variables. Logistic regression was used to control for variables independently associated with MDE. Statistical significance was accepted when p is greater than or equal to 0.05 (two-tailed)
Reanalysis	Not possible	Not required	Removing the 17 patients with medication-induced psychotic disorders, prevalence of psychosis in MDR-TB patients = 18.3% (21/115)	Not possible	Not required

For peer review only - http://bmjopen.bmj.com/site/about/guidelines.xhtml

PART 2	Koyanagi et al 2017	Hernández Sarmiento et al 2013	Lasebikan & Ige 2015	Srivastava et al 1983	de Castro-Silva et al 2019
**Applicability**					
Have important populations been excluded from the study?	No: Community-based survey (although excludes those without a valid home address, e.g. the institutionalised and the homeless who are at a high risk of TB)	Possibly: Only those homeless people attending one local health facility included	Yes: Only MDR-TB, and only one treatment centre<br>Are caregivers really representative controls?<br>Exclusion of participants with any affective disorder, affective psychosis or delirium could have distorted the prevalence<br>Also the exclusion of family members/caregivers with any psychiatric disorder	Yes: Extensive exclusion criteria with no justification	Possibly: Using patients of other respiratory illnesses as controls may not be representative
Does the study directly address the review question?	No: Treating depression as outcome and TB as exposure<br>Direction of causality cannot be established due to cross-sectional design	Yes: But direction of causality cannot be established due to cross-sectional design	No: Treating psychosis as outcome and MDR-TB as exposure<br>Direction of causality cannot be established due to cross-sectional design	No: Treating psychological state as outcome and TB as exposure<br>Direction of causality cannot be established due to cross-sectional design	Yes: But direction of causality cannot be established due to cross-sectional design
**Other Information**					
Key conclusions of study authors	TB is associated with the entire depression spectrum in the overall sample, and the association is comparable across regions and country income levels	Homeless people constitute a particular population with a high TB prevalence which co-exists with a high rate of mental disorders	Prevalence of psychosis among TB patients in this study is 33.0% (18.3% excluding medication-induced psychotic disorders compared to 2.7% among primary care givers	Significantly more patients as compared to control subjects are worried, anxious, depressed, irritable and have sleep and appetite disturbances	The prevalence of MDE (detected by the PHQ-9) among individuals with pre-PTB was high, and there was little difference between individuals with confirmed PTB and those with other respiratory illnesses (60.2 vs. 62.1%, respectively)
Correspondence with study authors	None	None	None	None	None

For peer review only

PRISMA 2009 Checklist

Section/topic	#	Checklist item	Reported on page #
**TITLE**			
Title	1	Identify the report as a systematic review, meta-analysis, or both.	1
**ABSTRACT**			
Structured summary	2	Provide a structured summary including, as applicable: background; objectives; data sources; study eligibility criteria, participants, and interventions; study appraisal and synthesis methods; results; limitations; conclusions and implications of key findings; systematic review registration number.	2-3
**INTRODUCTION**			
Rationale	3	Describe the rationale for the review in the context of what is already known.	4-5
Objectives	4	Provide an explicit statement of questions being addressed with reference to participants, interventions, comparisons, outcomes, and study design (PICOS).	5-6 and Table 1
**METHODS**			
Protocol and registration	5	Indicate if a review protocol exists, if and where it can be accessed (e.g., Web address), and, if available, provide registration information including registration number.	5
Eligibility criteria	6	Specify study characteristics (e.g., PICOS, length of follow-up) and report characteristics (e.g., years considered, language, publication status) used as criteria for eligibility, giving rationale.	5-6
Information sources	7	Describe all information sources (e.g., databases with dates of coverage, contact with study authors to identify additional studies) in the search and date last searched.	5
Search	8	Present full electronic search strategy for at least one database, including any limits used, such that it could be repeated.	Supp Box 1
Study selection	9	State the process for selecting studies (i.e., screening, eligibility, included in systematic review, and, if applicable, included in the meta-analysis).	5-6
Data collection process	10	Describe method of data extraction from reports (e.g., piloted forms, independently, in duplicate) and any processes for obtaining and confirming data from investigators.	6
Data items	11	List and define all variables for which data were sought (e.g., PICOS, funding sources) and any assumptions and simplifications made.	6
Risk of bias in individual studies	12	Describe methods used for assessing risk of bias of individual studies (including specification of whether this was done at the study or outcome level), and how this information is to be used in any data synthesis.	6-7
Summary measures	13	State the principal summary measures (e.g., risk ratio, difference in means).	7
Synthesis of results	14	Describe the methods of handling data and combining results of studies, if done, including measures of consistency (e.g., I^2) for each meta-analysis.	N/A

For peer review only - http://bmjopen.bmj.com/site/about/guidelines.xhtml

PRISMA 2009 Checklist

Section/topic	#	Checklist item	Reported on page #
Risk of bias across studies	15	Specify any assessment of risk of bias that may affect the cumulative evidence (e.g., publication bias, selective reporting within studies).	N/A
Additional analyses	16	Describe methods of additional analyses (e.g., sensitivity or subgroup analyses, meta-regression), if done, indicating which were pre-specified.	N/A
**RESULTS**			
Study selection	17	Give numbers of studies screened, assessed for eligibility, and included in the review, with reasons for exclusions at each stage, ideally with a flow diagram.	7 and Figure 1
Study characteristics	18	For each study, present characteristics for which data were extracted (e.g., study size, PICOS, follow-up period) and provide the citations.	Tables 2 and 3
Risk of bias within studies	19	Present data on risk of bias of each study and, if available, any outcome level assessment (see item 12).	Table 2
Results of individual studies	20	For all outcomes considered (benefits or harms), present, for each study: (a) simple summary data for each intervention group (b) effect estimates and confidence intervals, ideally with a forest plot.	Table 3
Synthesis of results	21	Present results of each meta-analysis done, including confidence intervals and measures of consistency.	N/A
Risk of bias across studies	22	Present results of any assessment of risk of bias across studies (see Item 15).	N/A
Additional analysis	23	Give results of additional analyses, if done (e.g., sensitivity or subgroup analyses, meta-regression [see Item 16]).	N/A
**DISCUSSION**			
Summary of evidence	24	Summarize the main findings including the strength of evidence for each main outcome; consider their relevance to key groups (e.g., healthcare providers, users, and policy makers).	11-15
Limitations	25	Discuss limitations at study and outcome level (e.g., risk of bias), and at review-level (e.g., incomplete retrieval of identified research, reporting bias).	12-13
Conclusions	26	Provide a general interpretation of the results in the context of other evidence, and implications for future research.	13-15
**FUNDING**			
Funding	27	Describe sources of funding for the systematic review and other support (e.g., supply of data); role of funders for the systematic review.	16

From: Moher D, Liberati A, Tetzlaff J, Altman DG, The PRISMA Group (2009). Preferred Reporting Items for Systematic Reviews and Meta-Analyses: The PRISMA Statement. PLoS Med 6(7): e1000097. doi:10.1371/journal.pmed1000097

For more information, visit: **www.prisma-statement.org**.

For peer review only - http://bmjopen.bmj.com/site/about/guidelines.xhtml

BMJ Open

The relationship between mental health and risk of active tuberculosis: A systematic review

Journal:	*BMJ Open*
Manuscript ID	bmjopen-2021-048945.R1
Article Type:	Original research
Date Submitted by the Author:	20-Jul-2021

Complete List of Authors:	Hayward, Sally; St George's University of London; London School of Hygiene & Tropical Medicine<br>Deal, Anna; St George's University of London; London School of Hygiene & Tropical Medicine<br>Rustage, Kieran; St George's University of London<br>Nellums, Laura ; University of Nottingham; St George's University of London<br>Sweetland, Annika; New York State Psychiatric Institute, Department of Psychiatry<br>Boccia, Delia; London School of Hygiene & Tropical Medicine<br>Hargreaves, Sally; St George's University of London<br>Friedland, Jon; St George's University of London

**Primary Subject Heading**:	Infectious diseases
Secondary Subject Heading:	Mental health, Global health, Epidemiology, Respiratory medicine
Keywords:	Tuberculosis < INFECTIOUS DISEASES, MENTAL HEALTH, Public health < INFECTIOUS DISEASES, Epidemiology < INFECTIOUS DISEASES

SCHOLARONE™
Manuscripts

I, the Submitting Author has the right to grant and does grant on behalf of all authors of the Work (as defined in the below author licence), an exclusive licence and/or a non-exclusive licence for contributions from authors who are: i) UK Crown employees; ii) where BMJ has agreed a CC-BY licence shall apply, and/or iii) in accordance with the terms applicable for US Federal Government officers or employees acting as part of their official duties; on a worldwide, perpetual, irrevocable, royalty-free basis to BMJ Publishing Group Ltd ("BMJ") its licensees and where the relevant Journal is co-owned by BMJ to the co-owners of the Journal, to publish the Work in this journal and any other BMJ products and to exploit all rights, as set out in our licence.

The Submitting Author accepts and understands that any supply made under these terms is made by BMJ to the Submitting Author unless you are acting as an employee on behalf of your employer or a postgraduate student of an affiliated institution which is paying any applicable article publishing charge ("APC") for Open Access articles. Where the Submitting Author wishes to make the Work available on an Open Access basis (and intends to pay the relevant APC), the terms of reuse of such Open Access shall be governed by a Creative Commons licence – details of these licences and which Creative Commons licence will apply to this Work are set out in our licence referred to above.

Other than as permitted in any relevant BMJ Author's Self Archiving Policies, I confirm this Work has not been accepted for publication elsewhere, is not being considered for publication elsewhere and does not duplicate material already published. I confirm all authors consent to publication of this Work and authorise the granting of this licence.

For peer review only

The relationship between mental health and risk of active tuberculosis: A systematic review

Sally E Hayward[1,2], Anna Deal[1,2], Kieran Rustage[1], Laura B Nellums[1,3], Annika C Sweetland[4], Delia Boccia[2*], Sally Hargreaves[1*], Jon S Friedland[1*]

[*]Joint senior authors

[1]Institute for Infection and Immunity, St George's, University of London, London, UK
[2]Faculty of Public Health and Policy, London School of Hygiene and Tropical Medicine, London, UK
[3]Division of Epidemiology and Public Health, School of Medicine, University of Nottingham, Nottingham, UK
[4]Department of Psychiatry, Columbia Vagelos College of Physicians and Surgeons, New York State Psychiatric Institute, New York, USA

Corresponding author:
Sally Hayward
Institute for Infection and Immunity
St George's, University of London
Cranmer Terrace, London
SW17 0RE, United Kingdom
Email: shayward@sgul.ac.uk

For peer review only

Abstract

Objectives. Tuberculosis (TB) and mental illnesses are highly prevalent globally and often co-exist. Whilst poor mental health is known to modulate immune function, whether mental disorders play a causal role in TB incidence is unknown. This systematic review examines the association between mental health and TB disease risk to inform clinical and public health measures.

Design. Systematic review, following PRISMA guidelines.

Search strategy and selection criteria. MEDLINE, PsycINFO and PsycEXTRA databases were searched alongside reference list and citation searching. Inclusion criteria were original research studies published 01/01/1970-11/05/2020 reporting data on the association between mental health and TB risk.

Data extraction, appraisal and synthesis. Data were extracted on study design and setting, sample characteristics, measurement of mental illness and TB, and outcomes including effect size or prevalence. Studies were critically appraised using CASP and AXIS checklists.

Results. 1546 records published over 50 years were screened, resulting in 10 studies included reporting data from 607,184 individuals. Studies span across Asia, South America, and Africa, and include mood and psychotic disorders. There is robust evidence from cohort studies in Asia demonstrating that depression and schizophrenia can increase risk of active TB, with effect estimates ranging from HR=1.15 (95% CI 1.03-1.28) to 2.63 (95% CI 1.74-3.96) for depression and HR=1.52 (95% CI 1.29-1.79) to RR=3.04 for schizophrenia. This data aligns with evidence from cross-sectional studies, for example a large survey across low- and middle-income countries (n=242,952) reports OR=3.36 (95% CI 3.01-4.50) for a depressive episode in those with TB symptoms versus those without.

For peer review only

Conclusions. Individuals with mental illnesses including depression and schizophrenia experience increased TB incidence and represent a high-risk population to target for screening and treatment. Integrated care for mental health and TB is needed, and interventions tackling mental illnesses and underlying drivers may help reduce TB incidence globally.

PROSPERO registration number. CRD42019158071

Strengths and limitations of this study

- We have undertaken a comprehensive examination of the evidence base on the relationship between mental health and tuberculosis (TB) incidence
- We carried out a systematic review following Preferred Reporting Items for Systematic Reviews and Meta-Analyses (PRISMA) guidelines
- We included data from a range of study designs, which created challenges in synthesis but enabled a holistic examination of all the available evidence

For peer review only

Introduction

Both tuberculosis (TB) and mental health are urgent global health priorities, with 1.4 million TB deaths worldwide in 2019 [1], and approximately 14% of the global burden of disease attributable to neuropsychiatric disorders [2]. There is increasing recognition that physical and mental health are interconnected [3, 4]. Mental illnesses are highly prevalent among TB patients and vice-versa [5, 6], and poor mental health is associated with reduced treatment-seeking and adherence, and therefore with greater morbidity, mortality, transmission, and drug resistance [7, 8]. Moreover, TB can infect the central nervous system (CNS) causing neurological symptoms [9], and certain anti-TB medications have psychiatric side effects [5]. The greatest burden of TB is experienced in individuals with risk factors including homelessness, drug and alcohol misuse, and migration [10]. Mental illness is more prevalent in all of these groups [11-13]. The relationships between TB and mental health are highly complex, with TB–depression comorbidity termed a 'syndemic' due to the bidirectional synergies involved [14].

Previous research has examined the impact of TB and its treatment on mental health [5], the relationship between mental disorders and TB treatment outcomes [7, 15] and adherence [8, 16], and the prevalence of mental disorders among TB and multidrug-resistant TB (MDR-TB) patients [17-19]. There is increasing interest in whether mental health may increase TB risk, supported by a growing body of evidence that chronic stressors and poor mental health directly influence the immune system, including susceptibility to infection [14, 20]. How these pathways operate in TB is unknown, although various mechanisms are possible; for instance the suppression of cellular immunity due to poor mental health could contribute to reactivation of latent TB infection (LTBI) or progression from sub-clinical to clinical disease

For peer review only - http://bmjopen.bmj.com/site/about/guidelines.xhtml

[21]. However, while the bi-directional relationship between mental health and TB has been the subject of discussion [6, 14, 21-25], there has been no systematic and comprehensive examination of the evidence base on associations between mental health and TB incidence. This systematic review aims to examine the evidence on the relationship between mental health and TB, to provide insight into whether poor mental health may be a risk factor for TB disease and to inform clinical and global public health measures.

Methods

Search strategy. We carried out a systematic review following PRISMA guidelines [26], registered with PROSPERO (CRD42019158071). MEDLINE, PsycINFO and PsycEXTRA were searched from inception to 11/5/2020, combining terms and subject headings for mental health and TB (Supplementary Box 1), restricting the search to English language papers. Records were imported into EndNote, and duplicates deleted. Two independent reviewers (SEH and KR/AD) carried out title/abstract and full-text screening using Rayyan QCRI [27]. We searched reference lists and carried out citation searching via Web of Science for included papers and previous reviews in this area [5, 7, 8, 15-19].

Selection criteria. This study includes empirical research reporting on the relationship between poor mental health and risk of active TB (Table 1). This includes two study designs: a) longitudinal studies investigating the causal relationship between mental health and TB incidence, and b) cross-sectional studies investigating the association between mental health and TB disease. Cross-sectional studies were included regardless of whether they treated mental health as the exposure and TB as the outcome or vice versa, since both provide

For peer review only - http://bmjopen.bmj.com/site/about/guidelines.xhtml

evidence for an association (provided that those with a history of mental illness are not excluded). Prevalence studies were excluded, as studies without controls cannot establish the presence of an association. No exclusions were made based on population, age, or geographic location.

The exposure measure was mental ill health, assessed either as an overall measure of mental illness or as a specific mental disorder. Mental disorders are defined here to include psychotic disorders (e.g. schizophrenia), mood or affective disorders (e.g. depression), and neurotic, stress-related and somatoform disorders (e.g. anxiety) [28]. Mental illness may be diagnosed clinically (i.e. by a medical professional and/or prescription of medication) or by any psychological tool (i.e. a structured questionnaire or interview). Only studies for which mental illness was a primary variable were included to ensure that the study was powered to detect an association. Studies focused solely on substance use disorders were excluded as alcohol and drug use are already known to be strongly associated with TB risk, possibly due to social mixing patterns, or the effect of these substances on the immune system [29-31].

The outcome measure was active TB disease, diagnosed according to bacteriological, clinical, and/or radiological criteria [32]. This excludes LTBI but includes reactivated disease. TB of any site, pulmonary or extrapulmonary, was included, with the exception of CNS TB. TB-HIV co-infection was also excluded. In these cases, different mechanisms are expected to underlie the associations. Studies relating to outcomes of TB infection or treatment (including psychiatric side effects of anti-TB medication), rather than incidence, were excluded, as this has been previously reviewed [7, 15].

For peer review only - http://bmjopen.bmj.com/site/about/guidelines.xhtml

Data extraction. Two independent reviewers (SEH and KR/AD) extracted data using a standardised form adapted from the Cochrane EPOC group [33], including study characteristics, population, setting, methods, participants, exposure, outcome, results (summary estimates), and applicability.

Critical appraisal. Two independent reviewers (SEH and KR/AD) assessed the quality of all included studies, using CASP checklists for cohort and case-control studies and the AXIS tool for cross-sectional studies [34, 35]. The tools were amended to remove questions on local applicability and implications for practice, instead asking about external validity and implications for the review. A study was defined as high quality at a critical appraisal score of above 90%, moderate above 60%, and low at 60% or below. This score relates to the appropriateness and relevance of the study for this review question rather than in relation to the study's own aims.

Synthesis. Extracted data were tabulated, detailing methods and results of included studies, categorised by type of mental illness. Results are presented as reported in the studies. The primary outcome extracted is the main effect measure for the relationship between mental health and TB, and the secondary outcome is any additional measures reported relating to different definitions or levels of mental illness. For cross-sectional studies, any cases of mental illness arising post-TB diagnosis or treatment were excluded where possible. Given the wide range of populations, study designs, and statistical methods used, meta-analysis was not appropriate. A narrative synthesis was therefore completed, by study design and type of mental illness. Effect size and precision were compared where studies were sufficiently similar. The evidence as a whole was assessed as to whether poor mental health may be associated with, and act as a risk factor for, TB disease.

For peer review only - http://bmjopen.bmj.com/site/about/guidelines.xhtml

Patient and public involvement. Patients and/or the public were not involved in the design, conduct, reporting or dissemination plans of this research.

Results

We screened 1546 records published over 50 years and found that 10 published articles met the inclusion criteria (Figure 1), with a combined sample size of 607,184. Reasons for exclusion are presented in Figure 1, with the most common being that mental health is not a primary outcome or that the study design does not meet the inclusion criteria outlined above.

Included studies span multiple locations, including five in Asia [36-40], three in South America [41-43], one in Africa [44], and one across low- and middle-income countries (LMICs) [45]. Two studies were published in the 1980s [38, 40] and the remainder within the last ten years [36, 37, 39, 41-45]. Seven studies assess mood disorders [36-38, 41-43, 45] and three assess psychotic disorders [39, 40, 44]. Seven studies investigate pulmonary TB exclusively [37, 38, 41-45], and three consider both pulmonary and extrapulmonary disease [36, 39, 40]. Study characteristics are summarised in Table 2. Only one paper included here was included in any prior systematic review of mental health and TB [5, 7, 8, 15-19].

Several study designs were used to investigate the association between mental health and TB. Four retrospective cohort studies use insurance or registry data to follow up TB incidence in those with a mental illness compared with matched controls [36, 37, 39] or general population incidence [40]. Where specified, the studies use International Classification of Diseases (ICD-

For peer review only

9 or ICD-10) codes on patient medical records, in addition to prescription of medication, to identify patients with a mental disorder and with TB disease. These are large studies, making up 59.6% of the total sample size (n=361,765) (Figure 2).

Other studies use questionnaire screening tools or interviews to assess mental health at a single timepoint. These may be short disorder-specific questionnaires, such as the Patient Health Questionnaire-9 (PHQ-9) for depression [46], or longer interviews covering a range of psychiatric disorders, such as the Composite International Diagnostic Interview (CIDI) [47]. One case-control study identifies mental illnesses prior to TB diagnosis in cases and matched controls [42]. Two population-based cross-sectional studies establish prevalence of mental illnesses and TB, and assess the association between them [43, 45]. Three additional cross-sectional studies compare point prevalence of mental illnesses in TB cases and controls [38, 41, 44]. One of the population-based cross-sectional studies is very large, making up 40.0% of the total sample size (n=242,952), whereas the other five cross-sectional studies are smaller, making up 0.4% of the total sample size (n=2,467) (Figure 2).

While the retrospective cohort studies use population-based databases or registers [36, 37, 39, 40], other studies are only applicable to certain populations such as the homeless [43] or MDR-TB patients [44]. Included studies vary in quality, with three high, four moderate and three low quality studies overall, and an average critical appraisal score of 71% for mood and 70% for psychotic disorder papers. The longitudinal studies are all moderate to high quality (average score 84%), whereas the cross-sectional studies are low to moderate quality (average score 62%). Common problems identified are issues with sampling and inadequate consideration of confounding. Results and study quality are tabulated in Table 3 and in detail in Supplementary Tables 1-2.

For peer review only - http://bmjopen.bmj.com/site/about/guidelines.xhtml

Mood disorders. Four studies specifically investigate depression [36, 37, 41, 45], and three look generally at mood disorders [38, 42, 43]. This includes two large, high-quality cohort studies on depression, both using nationally representative databases. One, in South Korea, reports an adjusted hazard ratio (HR) for TB incidence among those with depression compared with controls of 2.63 (95% confidence interval [CI] 1.74-3.96, p<0.001) [36], and the other, in Taiwan, reports an adjusted HR of 1.15 (95% CI 1.03-1.28) [37]. The former also finds a dose-response relationship, with more severe depression associated with greater TB risk. Both studies report a higher TB incidence rate in those with depression than in controls (Figure 3).

Other studies use a cross-sectional design to assess the association between mood disorders and TB. A large study using community-based World Health Survey data from across LMICs reports an adjusted odds ratio (OR) for a depressive episode in those with TB symptoms versus those without of 3.63 (95% CI 3.01-4.50) [45]. By contrast, a cross-sectional study among presumptive pulmonary TB cases in Rio de Janeiro, Brazil that compares the prevalence of depression among patients with and without a bacteriologically confirmed diagnosis of pulmonary TB finds no evidence of a difference. Of 259 participants screened for depression, the prevalence among TB cases is 60.2% versus 62.1% in controls (crude OR=0.92, 95% CI 0.55-1.54, p=0.79), and of 159 who screened positive for depression, this diagnosis was confirmed in 59.5% of TB cases versus 50.9% of controls (crude OR=1.42, 95% CI 0.63-3.19, p=0.42) [41].

Beyond depression, a case-control study in Salvador, Brazil considers common mental disorders (CMDs) more broadly, characterised by diverse depressive, anxiety, or somatoform

For peer review only - http://bmjopen.bmj.com/site/about/guidelines.xhtml

symptoms, and finds elevated odds of having TB among those with a CMD (adjusted OR=1.34, 95% CI 1.05-1.70) [42]. A cross-sectional study among the homeless in Medellín, Columbia investigates the association between TB and various psychiatric illnesses, but finds that only dysthymia (persistent mild depression) and history of major depression are associated with TB, and only for dysthymia is this statistically significant in multivariate analysis (adjusted OR=2.54, 95% CI 1.10-5.86, p=0.028) [43]. Finally, a cross-sectional study into the current psychological state of TB cases and controls in Bikaner, India finds a significant association between TB and symptoms including anxiety, depressed mood, and sleep/appetite disturbances, and reports a prevalence of psychiatric illness in TB patients of 41.6% versus 13.3% in controls [38].

Psychotic disorders. Two studies specifically investigate schizophrenia [39, 40], and one considers psychosis more generally [44]. The schizophrenia studies are retrospective cohort studies, both finding that schizophrenia is significantly associated with TB risk. A large, high quality study in Taiwan, using a nationwide database, reports an adjusted HR for TB incidence in those with schizophrenia versus controls of 1.52 (95% CI 1.29-1.79, p<0.001) [39]. The other, in Nagasaki, Japan uses registry data to identify TB incidence in those diagnosed with schizophrenia, and compares this to general population incidence. The reported relative risk (RR) for observed versus expected TB incidence is 3.04 (p<0.005) [40]. Both studies report a higher TB incidence rate in those with schizophrenia than in controls (Figure 3).

A cross-sectional study investigates psychosis more generally among patients attending an MDR-TB clinic in Ibadan, Nigeria. This reports a prevalence of psychosis of 33.0% in MDR-TB patients versus 2.7% in controls. When recalculated to exclude anti-TB medication-

For peer review only - http://bmjopen.bmj.com/site/about/guidelines.xhtml

induced psychotic disorders, this is a prevalence of 18.3% in MDR-TB patients. This includes a prevalence of schizophrenia of 8.7% in TB patients vs. 0.9% in controls (χ^2=5.9, p=0.02) [44].

Discussion

Included studies, synthesising data from 607,184 individuals, show an association between mental health and TB. This includes robust evidence from cohort studies, all of which were based in Asia, demonstrating that depression and schizophrenia increase risk of TB disease. While the co-existence of poor mental health and TB is consistent with existing literature [5], this is the first review to show that mental health is a risk factor for active TB.

Four population-based cohort studies are included, which are large studies using registry or insurance records to assess the relationship between medically-coded depression or schizophrenia and TB disease in nationwide samples in Asia. Other studies, which are generally much smaller and cross-sectional in design, use questionnaire or interview mental health screening tools carried out in community or healthcare settings across Asia, South America and Africa. They therefore can detect less severe and undiagnosed conditions and can focus on specific, vulnerable populations such as the homeless. Whereas the cohort studies consider specific conditions such as depression or schizophrenia, most of the cross-sectional studies use screening tools that capture a wider range of mental health conditions.

Despite significant heterogeneity in study design and population, nine out of ten included studies find a significant association between mental health and TB. In the one study that does

For peer review only

For peer review only - http://bmjopen.bmj.com/site/about/guidelines.xhtml

not find a significant association, controls are individuals with prolonged respiratory symptoms, who might be expected to have a higher prevalence of depression than the general population [41]. While not directly comparable, some of the observed variations may be attributed to different levels of adjustment for confounding; the cohort studies with more extensive adjustment for confounders [37, 39] show smaller effect sizes than their counterparts with less or no adjustment for confounders [36, 40].

A key strength of this study is that, through undertaking an exhaustive search of the literature, it brings together a large sample of over 600,000 individuals. By synthesising evidence from a range of study designs, we collate all the evidence on this topic and analyse the findings holistically. Nevertheless, this review has certain limitations. Future reviews could focus on LTBI, CNS TB, and TB-HIV co-infection, which were excluded from this study. Moreover, the heterogeneity in study populations and methods made drawing direct comparisons between studies difficult and made meta-analysis inappropriate. For example, it was not possible to directly compare measures of effect generated from rates (HRs) with those generated from risks (RRs/ORs).

The nature of available evidence means that some caution is required when drawing conclusions. Studies addressing the association between mental health and TB are of variable design and quality, with four large studies (three cohort and one cross-sectional) accounting for 99% of included participants [36, 37, 39, 45]. Three included studies were classified as low quality; however, these only account for 0.001% of the total sample size (773 participants) [38, 43, 44]. Only the cohort studies can establish temporality to provide compelling evidence that mental health precedes and acts as a risk factor for TB, and these are the highest quality studies [36, 37, 39, 40]. However, the cohort studies only cover depression

For peer review only

For peer review only - http://bmjopen.bmj.com/site/about/guidelines.xhtml

and schizophrenia and are all from Asia. Further cohort studies are required to confirm whether these findings hold true for other mental illnesses in other global regions.

The study findings increase our understanding of TB risk factors, yet further research is needed to elucidate the pathways by which mental health may increase TB incidence, causally or via associations between mental health and other risk factors for TB such as alcohol/drug use, homelessness, incarceration, physical co-morbidities, and poverty. The relationships between mental health, TB, and social risk factors are multi-directional, meaning that complex conceptual frameworks will be needed to understand the observed associations. The existence of plausible immune mechanisms supports a causal explanation, with evidence that psychosocial stressors are associated with immune biomarkers relevant to TB [48]. Mental illnesses including depression are associated with various immunological changes which could increase susceptibility to TB [14, 49]; however, the key neuroendocrine and immunological pathways involved are unknown. In addition, mental health could be one factor influencing risk of progression from LTBI to active disease. Understanding the pathways that connect mental health and the immune response to TB may guide the development of host-directed approaches, for example to prevent reactivation of LTBI.

The findings have implications for policy and clinical practice. The *Lancet* Commission on TB recommends that, to achieve a TB-free world, populations at high risk must be reached and brought into care [50]. The evidence presented here suggests that those suffering from mental illnesses, in particular depression and schizophrenia, could constitute such a high-risk group for active case-finding and treatment. This group could benefit from a holistic approach, integrating services for mental health and TB to facilitate rapid diagnosis and treatment of TB disease and LTBI, as well as providing better mental health support for

For peer review only - http://bmjopen.bmj.com/site/about/guidelines.xhtml

individuals with TB. As such, the World Health Organization End TB Strategy 2015-35 recommends that treatment for TB and mental health is brought together [51]. Providing high-quality mental health support and ensuring treatment adherence for TB both require substantial engagement with patients, so leveraging this contact to provide holistic care could prove effective [52].

In addition, the finding that mental illnesses constitute risk factors for TB suggests that tackling poor mental health and its underlying drivers may reduce TB incidence. In LMICs with a high TB incidence, poverty is consistently associated with common mental disorders [53]. Social protection schemes that lift individuals out of poverty are known to improve mental health and reduce TB risk factors [54-56], suggesting that tackling poverty and associated poor mental health through investment in wider social policies could help reduce TB incidence [57].

Conclusion

We find evidence that mental health is a risk factor for active TB. There is robust evidence from cohort studies based in Asia that depression and schizophrenia increase incidence of TB disease. This data, in combination with evidence from cross-sectional studies, identifies individuals with mental illnesses as a high-risk population for clinical TB that could be targeted for screening and treatment. This highlights the need for integrated programmes providing care for mental health and TB and suggests that interventions that tackle mental illnesses and their underlying drivers may help reduce TB incidence globally.

For peer review only - http://bmjopen.bmj.com/site/about/guidelines.xhtml

Contributors

SEH and DB developed the concept and study design. SEH, AD and KR carried out screening, data extraction, critical appraisal and analysis. All authors (SEH, AD, KR, LBN, ACS, DB, SH, JSF) contributed to critical review of data and manuscript writing.

Funding

SEH and AD are supported by Medical Research Council PhD studentships (MR/N013638/1), and KR receives funding from the Rosetrees Trust (M775). SH is supported by the National Institute for Health Research (NIHR Advanced Fellowship NIHR300072) and the Academy of Medical Sciences (SBF005\1111), and the European Society for Clinical Microbiology and Infectious Diseases (ESCMID) through an ESCMID Study Group for Infections in Travellers and Migrants (ESGITM)/ESCMID Study Group for Mycobacterial Infection (ESGMYC) research grant. LBN receives funding from the Academy of Medical Sciences (SBF005\1047), the Medical Research Council/Economic and Social Research Council/Arts and Humanities Research Council (MR/T046732/1), and the Medical Research Council (MR/V027549/1). ACS is supported by the US National Institute of Mental Health (K01 MH104514).

Competing interests

We declare no competing interests.

Data sharing statement

All data relevant to the study are included in the article or uploaded as supplementary information.

For peer review only

For peer review only - http://bmjopen.bmj.com/site/about/guidelines.xhtml

References

1. World Health Organization. Global tuberculosis report 2020. Geneva: WHO; 2020.

2. Mathers CD, Loncar D. Projections of Global Mortality and Burden of Disease from 2002 to 2030. PLOS Medicine. 2006;3(11):e442.

3. Prince M, Patel V, Saxena S, Maj M, Maselko J, Phillips MR, et al. No health without mental health. Lancet. 2007;370(9590):859-77.

4. Wang J, Wu X, Lai W, Long E, Zhang X, Li W, et al. Prevalence of depression and depressive symptoms among outpatients: a systematic review and meta-analysis. BMJ Open. 2017;7(8):e017173.

5. Doherty AM, Kelly J, McDonald C, O'Dywer AM, Keane J, Cooney J. A review of the interplay between tuberculosis and mental health. Gen Hosp Psychiatry. 2013;35(4):398-406.

6. Sweetland A, Oquendo M, Wickramaratne P, Weissman M, Wainberg M. Depression: a silent driver of the global tuberculosis epidemic. World Psychiatry. 2014;13(3):325-6.

7. Ruiz-Grosso P, Cachay R, de la Flor A, Schwalb A, Ugarte-Gil C. Association between tuberculosis and depression on negative outcomes of tuberculosis treatment: A systematic review and meta-analysis. PLoS ONE. 2020;15(1):e0227472.

8. Pachi A, Bratis D, Moussas G, Tselebis A. Psychiatric Morbidity and Other Factors Affecting Treatment Adherence in Pulmonary Tuberculosis Patients. Tuberculosis Research and Treatment. 2013;2013:489865.

9. Rock RB, Olin M, Baker CA, Molitor TW, Peterson PK. Central nervous system tuberculosis: pathogenesis and clinical aspects. Clinical microbiology reviews. 2008;21(2):243-61.

10. Public Health England. Tuberculosis in England: 2019 report. London: PHE; 2019.

For peer review only

11. Fazel S, Khosla V, Doll H, Geddes J. The Prevalence of Mental Disorders among the Homeless in Western Countries: Systematic Review and Meta-Regression Analysis. PLOS Medicine. 2008;5(12):e225.

12. Jané-Llopis E, Matytsina I. Mental health and alcohol, drugs and tobacco: a review of the comorbidity between mental disorders and the use of alcohol, tobacco and illicit drugs. Drug and Alcohol Review. 2006;25(6):515-36.

13. Priebe S, Giacco D, El-Nagib R. Public health aspects of mental health among migrants and refugees: a review of the evidence on mental health care for refugees, asylum seekers and irregular migrants in the WHO European Region (Health Evidence Network (HEN) Synthesis Report 47). Copenhagen: WHO Regional Office for Europe; 2016.

14. Sweetland AC, Kritski A, Oquendo MA, Sublette ME, Norcini Pala A, Silva LRB, et al. Addressing the tuberculosis-depression syndemic to end the tuberculosis epidemic. Int J Tuberc Lung Dis. 2017;21(8):852-61.

15. Lee GE, Scuffell J, Galea JT, Shin S, Magill E, Jaramillo E, et al. Impact of mental disorders on active TB treatment outcomes: a systematic review and meta-analysis. Int J Tuberc Lung Dis. 2020;24(12):1279-84.

16. Munro SA, Lewin SA, Smith HJ, Engel ME, Fretheim A, Volmink J. Patient Adherence to Tuberculosis Treatment: A Systematic Review of Qualitative Research. PLOS Medicine. 2007;4(7):e238.

17. Alene KA, Clements ACA, McBryde ES, Jaramillo E, Lonnroth K, Shaweno D, et al. Mental health disorders, social stressors, and health-related quality of life in patients with multidrug-resistant tuberculosis: A systematic review and meta-analysis. The Journal of infection. 2018;77(5):357-67.

For peer review only

18. Duko B, Bedaso A, Ayano G. The prevalence of depression among patients with tuberculosis: a systematic review and meta-analysis. Annals of general psychiatry. 2020;19:30.

19. Duko B, Dana LM, Ayano G. Psychological distress among TB patients in sub-Saharan Africa. Int J Tuberc Lung Dis. 2020;24(11):1200-4.

20. Glaser R, Kiecolt-Glaser JK. Stress-induced immune dysfunction: implications for health. Nat Rev Immunol. 2005;5(3):243-51.

21. Zhang K, Wang X, Tu J, Rong H, Werz O, Chen X. The interplay between depression and tuberculosis. Journal of leukocyte biology. 2019;106(3):749-57.

22. Mason PH, Sweetland AC, Fox GJ, Halovic S, Nguyen TA, Marks GB. Tuberculosis and mental health in the Asia-Pacific. Australasian Psychiatry. 2016;24(6):553-5.

23. Janse Van Rensburg A, Dube A, Curran R, Ambaw F, Murdoch J, Bachmann M, et al. Comorbidities between tuberculosis and common mental disorders: a scoping review of epidemiological patterns and person-centred care interventions from low-to-middle income and BRICS countries. Infect Dis Poverty. 2020;9(1):4.

24. Chandra M, Rana P, Chandra K, Arora VK. Tuberculosis - Depression syndemic: A public health challenge. The Indian journal of tuberculosis. 2019;66(1):197-202.

25. de Araújo GS, Pereira SM, dos Santos DN. Revisão sobre tuberculose e transtornos mentais comuns. Revista Eletrônica Gestão & Saúde. 2014;5(2):716-26.

26. Moher D, Liberati A, Tetzlaff J, Altman DG, The Prisma Group. Preferred Reporting Items for Systematic Reviews and Meta-Analyses: The PRISMA Statement. PLOS Medicine. 2009;6(7):e1000097.

27. Ouzzani M, Hammady H, Fedorowicz Z, Elmagarmid A. Rayyan—a web and mobile app for systematic reviews. Systematic Reviews. 2016;5(1):210.

For peer review only - http://bmjopen.bmj.com/site/about/guidelines.xhtml

28. World Health Organization. The ICD-10 classification of mental and behavioural disorders: diagnostic criteria for research. Geneva: WHO; 1993.

29. Lönnroth K, Williams BG, Stadlin S, Jaramillo E, Dye C. Alcohol use as a risk factor for tuberculosis – a systematic review. BMC Public Health. 2008;8(1):289.

30. Imtiaz S, Shield KD, Roerecke M, Samokhvalov AV, Lönnroth K, Rehm J. Alcohol consumption as a risk factor for tuberculosis: meta-analyses and burden of disease. Eur Respir J. 2017;50(1):1700216.

31. Deiss RG, Rodwell TC, Garfein RS. Tuberculosis and illicit drug use: review and update. Clinical infectious diseases : an official publication of the Infectious Diseases Society of America. 2009;48(1):72-82.

32. World Health Organization. Definitions and reporting framework for tuberculosis – 2013 revision (updated December 2014 and January 2020). Geneva: WHO; 2020.

33. Cochrane Effective Practice and Organisation of Care (EPOC). Data collection form. EPOC Resources for review authors. Available from: http://epoc.cochrane.org/epoc-specific-resources-review-authors [Accessed 28th March 2018]. 2017.

34. Critical Appraisal Skills Programme. CASP Checklists. Available from: https://casp-uk.net/casp-tools-checklists/ [Accessed 28th March 2018]. 2018.

35. EpiNet. AXIS - Appraisal Tool. Available from: http://www.epinet.net/AXIS-Appraisal-Tool [Accessed 25th March 2018].

36. Oh KH, Choi H, Kim EJ, Kim HJ, Cho SI. Depression and risk of tuberculosis: a nationwide population-based cohort study. Int J Tuberc Lung Dis. 2017;21(7):804-9.

37. Cheng KC, Liao KF, Lin CL, Lai SW. Increased Risk of Pulmonary Tuberculosis in Patients with Depression: A Cohort Study in Taiwan. Front Psychiatry. 2017;8:235.

38. Srivastava D, Gupta LN, Vyas JN. Psychological aspects of pulmonary tuberculosis. Indian Journal of Clinical Psychology. 1983;10(1):165-9.

For peer review only

For peer review only - http://bmjopen.bmj.com/site/about/guidelines.xhtml

39. Kuo SC, Chen YT, Li SY, Lee YT, Yang AC, Chen TL, et al. Incidence and outcome of newly-diagnosed tuberculosis in schizophrenics: a 12-year, nationwide, retrospective longitudinal study. BMC Infect Dis. 2013;13:351.

40. Ohta Y, Nakane Y, Mine M, Nakama I, Michitsuji S, Araki K, et al. The epidemiological study of physical morbidity in schizophrenics--2. Association between schizophrenia and incidence of tuberculosis. Jpn J Psychiatry Neurol. 1988;42(1):41-7.

41. Castro-Silva KM, Carvalho AC, Cavalcanti MT, Martins PDS, Franca JR, Oquendo M, et al. Prevalence of depression among patients with presumptive pulmonary tuberculosis in Rio de Janeiro, Brazil. Revista brasileira de psiquiatria (Sao Paulo, Brazil : 1999). 2019;41(4):316-23.

42. de Araújo GS, Pereira SM, dos Santos DN, Marinho JM, Rodrigues LC, Barreto ML. Common mental disorders associated with tuberculosis: a matched case-control study. PLoS ONE. 2014;9(6):e99551.

43. Hernandez Sarmiento JM, Correa N, Correa M, Franco JG, Alvarez M, Ramirez C, et al. Tuberculosis among homeless population from Medellin, Colombia: Associated mental disorders and socio-demographic characteristics. Journal of Immigrant and Minority Health. 2013;15(4):693-9.

44. Lasebikan VO, Ige OM. Prevalence of psychosis in tuberculosis patients and their nontuberculosis family contacts in a multidrug treatment-resistant treatment center in Nigeria. Gen Hosp Psychiatry. 2015;37(6):542-7.

45. Koyanagi A, Vancampfort D, Carvalho AF, DeVylder JE, Haro JM, Pizzol D, et al. Depression comorbid with tuberculosis and its impact on health status: cross-sectional analysis of community-based data from 48 low- and middle-income countries. BMC Med. 2017;15:209.

For peer review only

46. Kroenke K, Spitzer RL, Williams JB. The PHQ-9: validity of a brief depression severity measure. J Gen Intern Med. 2001;16(9):606-13.

47. World Health Organization. Composite International Diagnostic Interview (CIDI): Core Version 1.1. Geneva: WHO; 1993.

48. Hayward SE, Dowd JB, Fletcher H, Nellums LB, Wurie F, Boccia D. A systematic review of the impact of psychosocial factors on immunity: Implications for enhancing BCG response against tuberculosis. SSM - Population Health. 2020;10:100522.

49. Miller AH. Depression and immunity: a role for T cells? Brain Behav Immun. 2010;24(1):1-8.

50. Reid MJA, Arinaminpathy N, Bloom A, Bloom BR, Boehme C, Chaisson R, et al. Building a tuberculosis-free world: The Lancet Commission on tuberculosis. The Lancet. 2019.

51. World Health Organization. Implementing the End TB Strategy: the essentials. Geneva: WHO; 2015.

52. Sweetland AC, Galea J, Shin SS, Driver C, Dlodlo RA, Karpati A, et al. Integrating tuberculosis and mental health services: global receptivity of national tuberculosis program directors. Int J Tuberc Lung Dis. 2019;23(5):600-5.

53. Lund C, Breen A, Flisher AJ, Kakuma R, Corrigall J, Joska JA, et al. Poverty and common mental disorders in low and middle income countries: A systematic review. Soc Sci Med. 2010;71(3):517-28.

54. Ozer EJ, Fernald LCH, Weber A, Flynn EP, VanderWeele TJ. Does alleviating poverty affect mothers' depressive symptoms? A quasi-experimental investigation of Mexico's Oportunidades programme. International Journal of Epidemiology. 2011;40(6):1565-76.

For peer review only

55. Powell-Jackson T, Pereira SK, Dutt V, Tougher S, Haldar K, Kumar P. Cash transfers, maternal depression and emotional well-being: Quasi-experimental evidence from India's Janani Suraksha Yojana programme. Soc Sci Med. 2016;162:210-8.

56. Boccia D, Hargreaves J, Lonnroth K, Jaramillo E, Weiss J, Uplekar M, et al. Cash transfer and microfinance interventions for tuberculosis control: review of the impact evidence and policy implications. Int J Tuberc Lung Dis. 2011;15(Suppl 2):37-49.

57. Shete PB, Reid M, Goosby E. Message to world leaders: we cannot end tuberculosis without addressing the social and economic burden of the disease. The Lancet Global health. 2018;6(12):e1272-e3.

For peer review only

Figure captions

Figure 1: Study selection, PRISMA flow chart

PRISMA=Preferred Reporting Items for Systematic Reviews and Meta-Analyses

Figure 2: Sample size of included studies

Figure 3: TB incidence rate reported in longitudinal studies

TB=tuberculosis

For peer review only

For peer review only - http://bmjopen.bmj.com/site/about/guidelines.xhtml

Tables

Table 1: Inclusion criteria, using PECOS framework

	Inclusion criteria	Exclusion criteria
**Population**	Any population in any geographic location, including vulnerable groups e.g. the homeless	
**Exposure**	Mental illness, including psychotic and affective disorders, diagnosed clinically or by any psychological tool	- Alcohol and drug disorders<br>- Studies where mental illness is not a primary variable
**Control**	No mental illness	
**Outcome**	Incidence of active TB in humans, including reactivation of LTBI and MDR-TB	- LTBI without reactivation<br>- CNS TB (including tuberculosis meningitis)<br>- TB-HIV co-infection<br>- Studies investigating outcomes e.g. mortality (and not incidence)
**Study Design**	Observational epidemiological studies<br>a) Longitudinal studies (cohort): mental health must be treated as the exposure and TB as the outcome<br>b) Cross-sectional studies (cross-sectional and case-control): mental health may be treated as the exposure and TB as the outcome, or vice versa	- Prevalence studies without controls<br>- Qualitative studies<br>- Case reports<br>- Non-original research e.g. protocols, commentaries, reviews<br>- Longitudinal studies in which TB is the exposure and mental health is the outcome<br>- Cross-sectional studies that exclude those with a history of mental illness
**Dates**	Papers published 1970-2020	

CNS=central nervous system, HIV=human immunodeficiency virus, LTBI=latent tuberculosis infection, PECOS=population, exposure, control, outcome, study design, MDR-TB=multi-drug resistant tuberculosis, TB=tuberculosis

For peer review only - http://bmjopen.bmj.com/site/about/guidelines.xhtml

Table 2: Characteristics of all included studies

	Population		Study design				Exposure assessment		Outcome assessment		Controls	Critical Appraisal*
	Country	Gender & age	Design	Setting	Study period	*N*	Mental disorder	Measurement	Site of TB	Measurement		
**Mood disorders**												
Longitudinal studies												
**Oh et al 2017** [36]	South Korea	34% male; mean 45.8 years (SD 18.4)	Retrospective cohort study	Nationwide database	2003-13	64,744	Depression	ICD-10 codes plus psychotherapy prescription	Any	ICD-10 codes plus anti-TB drug prescription	No mood disorders, matched by age and sex	High (10/11, 91%)
**Cheng et al 2017** [37]	Taiwan	62% male; mean 47.9 years (SD 16.5) in cases and 47.6 (16.6) in controls	Retrospective cohort study	Nationwide database	2000-13	172,952	Depression	ICD-9 codes	Pulmonary	ICD-9 codes	No depression, matched for age, sex and comorbidities	High (10/11, 91%)
Cross-sectional studies												
**Koyanagi et al 2017** [45]	LMICs	49% male; mean 38.4 years (SD 16.1)	Cross-sectional study (population-based)	Community-based in 48 countries	2002-04	242,952	Depression	Interview (World Mental Health Survey version of the CIDI)	Pulmonary	TB symptoms in past 12 months (cough for three weeks or longer, blood in phlegm)	N/A	Moderate (17/20, 85%)
**De Castro-Silva et al 2019** [41]	Brazil	63% male in cases, 61% in controls; mean 40.7 years (SD 15.7) in cases, 46.9 (SD 16.0) in controls	Cross-sectional study (comparing point prevalence in cases and controls)	Municipal health centre in Rio de Janeiro	2015-16	260	Depression	Questionnaire (PHQ-9) and interview (MINI-Plus)	Pulmonary	Smear microscopy and/or Xpert MTB/RIF	Patients without TB, who were suspected to have pulmonary TB before testing	Moderate (13/20, 65%)
**De Araújo et al 2014** [42]	Brazil	61% male; mean 38.2 years (SD 14.2)	Case-control study	3 referral hospitals and 6 community clinics in Salvador	2008-10	1,434	Common Mental Disorders	Questionnaire (SRQ-20)	Pulmonary	Smear microscopy and culture for *M.tb*	Non-TB symptomatic respiratory patients, age- and sex-matched	Moderate (8/10, 80%)
**Hernández**	Columbia	83% male; mean 38.9	Cross-sectional	Homeless population	2006-07	426	Mental disorders	Interview (MINI)	Pulmonary	Smear microscopy	N/A	Low (12/20, 60%)

For peer review only

**Sarmiento et al 2013** [43]		years (SD 10.4)	study (population-based)	at a local health facility in Medellín								
**Srivastava et al 1983** [38]	India	67% male; age not available	Cross-sectional study (comparing point prevalence in cases and controls)	Hospital for TB and chest diseases, Bikaner	Not stated	120	Psychological state	Interview (PSE)	Pulmonary	Not specified	Non-TB patients at the hospital	Low (5/20, 25%)
**Psychotic disorders**												
Longitudinal studies												
**Kuo et al 2013** [39]	Taiwan	55% male; median 35.4 years in cases, 35.3 in controls	Retrospective cohort study	Nationwide database	1998-2009	120,818	Schizophrenia	ICD-9 codes	Any	ICD-9 codes plus anti-TB drug prescription	No schizophrenia, matched for age, sex, index date and comorbidities	High (10/11, 91%)
**Ohta et al 1988** [40]	Japan	55% male; age not available	Retrospective cohort study	Nationwide database	1960-78	3,251	Schizophrenia	Diagnosis (not further specified)	Any	Diagnosis (not further specified)	Expected incidence based on annual incidence rate	Moderate (7/11, 64%)
Cross-sectional study												
**Lasebikan & Ige 2015** [44]	Nigeria	38% male in cases, 18% in controls; median 35 years in cases, 42 in controls	Cross-sectional study (comparing point prevalence in cases and controls)	MDR-TB treatment centre, Ibadan	2010-14	227	Psychosis	Questionnaire (GHQ-12) and interview (psychosis screening questionnaire plus Structured Clinical Interview for DSM-IV Axis I Disorder, psychosis module)	Pulmonary	Attendance at MDR-TB treatment centre (not further specified)	Accompanying family members or caregivers	Low (11/20, 55%)

CIDI=Composite International Diagnostic Interview, CMD=common mental disorder, DSM=diagnostic and statistical manual, GHQ=general health questionnaire, ICD=international classification of diseases, LMIC=low- and middle-income country, *M.tb*/MTB=Mycobacterium tuberculosis, MDR=multi-drug resistant, MINI=Mini-International Neuropsychiatric Interview, N=sample size, PHQ=patient health questionnaire, PSE=present state interview, RIF-rifampicin, SD=standard deviation, SRQ=self-reporting questionnaire, TB=tuberculosis

*Critical appraisal score is defined based on number of positive responses to the questions on Critical Appraisal Skills Programme (CASP) checklists for cohort and case-control studies and the Appraisal Tool for Cross-Sectional Studies (AXIS) for cross-sectional studies

For peer review only - http://bmjopen.bmj.com/site/about/guidelines.xhtml

Table 3: Summary of study findings

	Primary outcome		Secondary outcomes		Statistical method	Adjustment
	Effect measure	Results	Effect measure	Results		
**Mood disorders**						
Longitudinal studies						
**Oh et al 2017** [36]	Adjusted HR for TB incidence in depressed vs. controls	2.63 (95% CI 1.74-3.96, p<0.001)	Adjusted HRs stratified by mild and severe depression	For mild depression = 1.99 (95% CI 1.21-3.28, p=0.007), for severe depression = 3.08 (95% CI 2.00-4.73 p<0.0001); linear dose-response	Cox proportional hazards model	Age, sex, income level, DM, COPD, alcoholism
**Cheng et al 2017** [37]	Adjusted HR for pulmonary TB incidence in depressed vs. controls	1.15 (95% CI 1.03-1.28)	-	-	Cox proportional hazards model	Age, sex, alcohol-related disease, CKD, chronic liver disease, COPD, DM, HIV infection, gastrectomy, and pneumoconiosis
Cross-sectional studies						
**Koyanagi et al 2017** [45]	Adjusted OR for depressive episode in those with pulmonary TB vs. those without	3.68 (95% CI 3.01-4.50, p<0.0001)	Adjusted ORs for brief depressive episode and for subsyndromal depression	For brief depressive episode = 1.75 (95% CI 1.26-2.42, p=0.0008), for subsyndromal depression = 1.98 (95% CI 1.47-2.67, p<0.0001)	Logistic regression	Age, sex, education, wealth, household size, setting, current smoking, alcohol consumption, BMI, diabetes, country
**De Castro-Silva et al 2019** [41]	Prevalence of depression in pulmonary TB patients; crude OR	Screening tool: 60.2% vs. 62.1% in controls (OR = 0.92, 95% CI 0.55-1.54, p=0.79) Diagnostic tool: 59.5% vs. 50.9% in controls (OR = 1.42, 95% CI 0.63-3.19, p=0.42)	-	-	Logistic regression	-
**De Araújo et al 2014** [42]	Adjusted OR for pulmonary TB in those with a CMD vs. those without	1.34 (95% CI 1.05-1.70)	-	-	Logistic regression	Diabetes, alcohol abuse, ethnicity, drug use, number of household goods, level of education, history of contact and crowding
**Hernández Sarmiento**	Crude and adjusted ORs for pulmonary	Dysthymia (adjusted) = 2.54 (95% CI 1.10-5.86,	-	-	Logistic regression	Age, gender, time living on the streets, education level,

For peer review only - http://bmjopen.bmj.com/site/about/guidelines.xhtml

**et al 2013** [43]	TB in those with mental disorders	p=0.028), dysthymia (crude) = 2.66 (95% CI 1.19-5.19, p=0.013), previous history of major depression (crude) = 2.22 (95% CI 1.07-4.6, p=0.028)				marital status, interaction with other homeless people, income sources
**Srivastava et al 1983** [38]	Prevalence of psychiatric illness in pulmonary TB patients vs. controls	41.6% vs. 13.3% in controls (p<0.001)	Prevalence of at least one psychiatric symptom in pulmonary TB patients	86.6% vs 70.0% in controls	Chi-square test	-
**Psychotic disorders**						
Longitudinal studies						
**Kuo et al 2013** [39]	Adjusted HR for TB incidence in those with schizophrenia vs. controls	1.52 (95% CI 1.29-1.79, p<0.001)	-	-	Cox proportional hazards model	Age, sex, Charlson's score, COPD, DM, rheumatoid disease, peptic ulcer disease, liver disease, hypertension, arrhythmia, dyslipidemia, drug or substance abuse, CKD, cancer, heart failure, peripheral vascular disease, myocardial infarction, hemiplegia/paraplegia, AIDS
**Ohta et al 1988** [40]	RR for TB incidence observed in those with schizophrenia vs. expected based on general population rates	3.04 (p<0.005)	-	-	Observed vs expected relative risk	Age, sex
Cross-sectional study						
**Lasebikan & Ige 2015** [44]	Prevalence of psychosis and schizophrenia in MDR-TB patients vs. controls	Psychosis: 33.0% (18.3% excluding medication-induced psychotic disorders‡) vs. 2.7% in controls	Prevalence of psychiatric morbidity in MDR-TB patients vs. controls	81.7% vs. 51.7% in controls (χ^2=21.7, p<0.001)	Chi-square test	-

For peer review only - http://bmjopen.bmj.com/site/about/guidelines.xhtml

		Schizophrenia: 8.7% vs. 0.9% in controls (χ^2=5.9, p=0.02)				

‡Recalculated to exclude mental health conditions that arise after TB diagnosis or treatment

AIDS=acquired immunodeficiency syndrome, BMI=body mass index, CI=confidence interval, CKD=chronic kidney disease, CMD=common mental disorder, COPD=chronic obstructive pulmonary disease, DM=diabetes mellitus, HIV=human immunodeficiency virus, HR=hazard ratio, MDR=multi-drug resistant, OR=odds ratio, RR=relative risk, SEP=socio-economic position, TB=tuberculosis

For peer review only

For peer review only - http://bmjopen.bmj.com/site/about/guidelines.xhtml

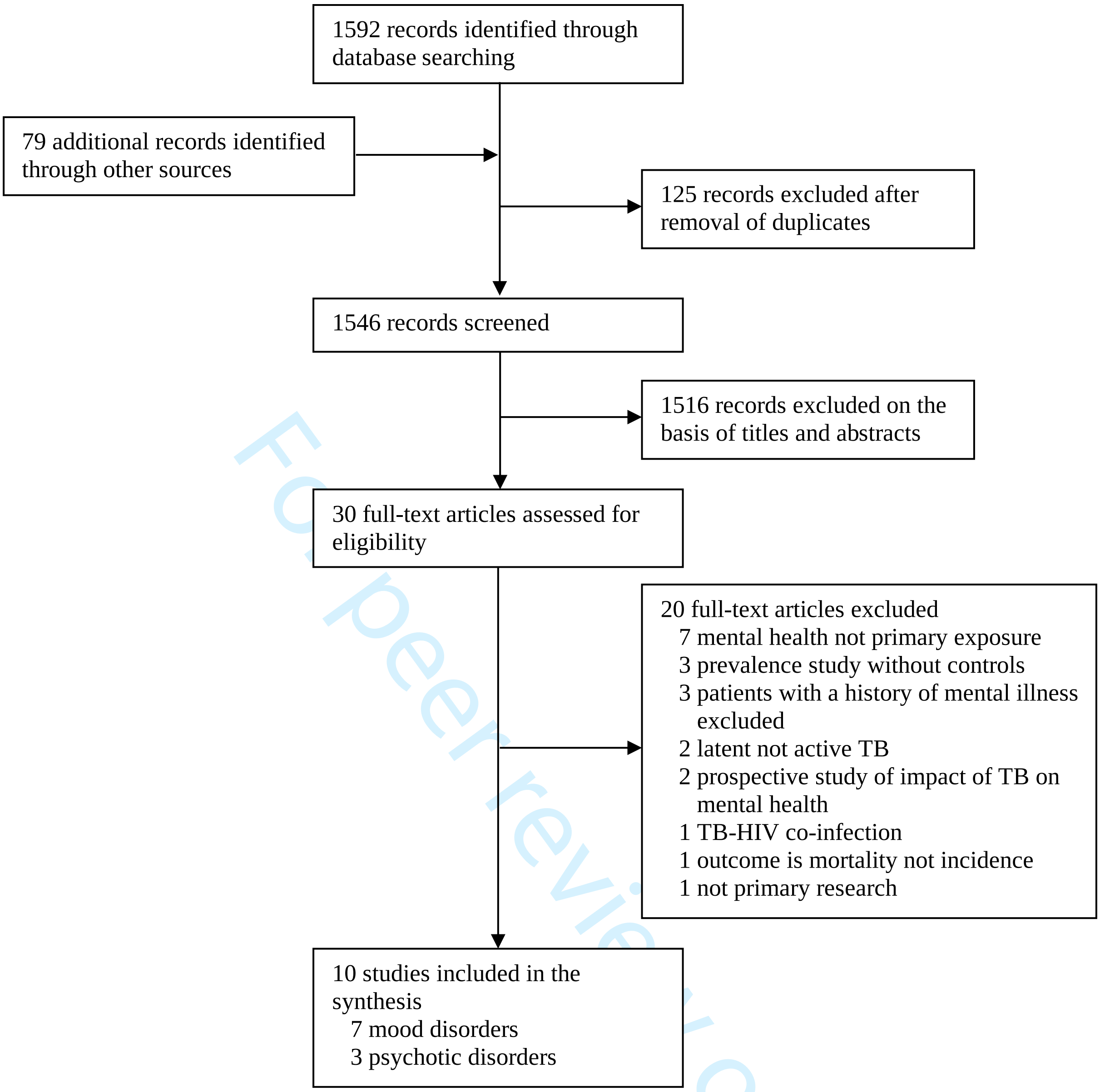

For peer review only

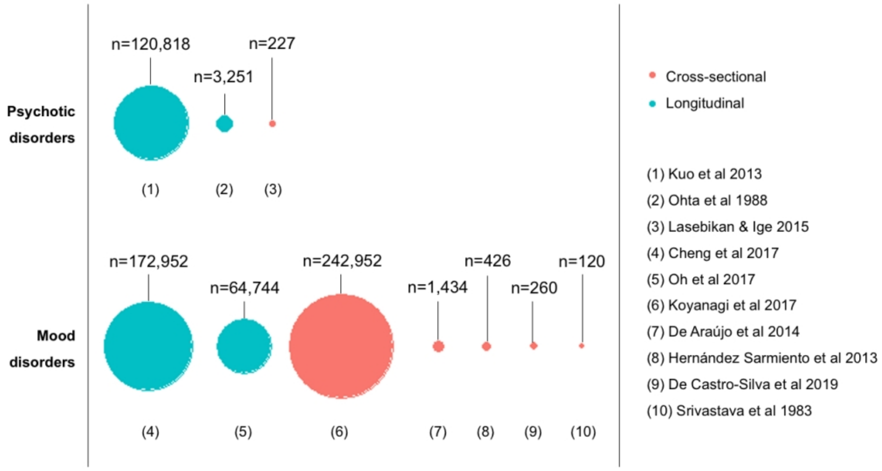

Figure 2: Sample size of included studies

338x190mm (108 x 108 DPI)

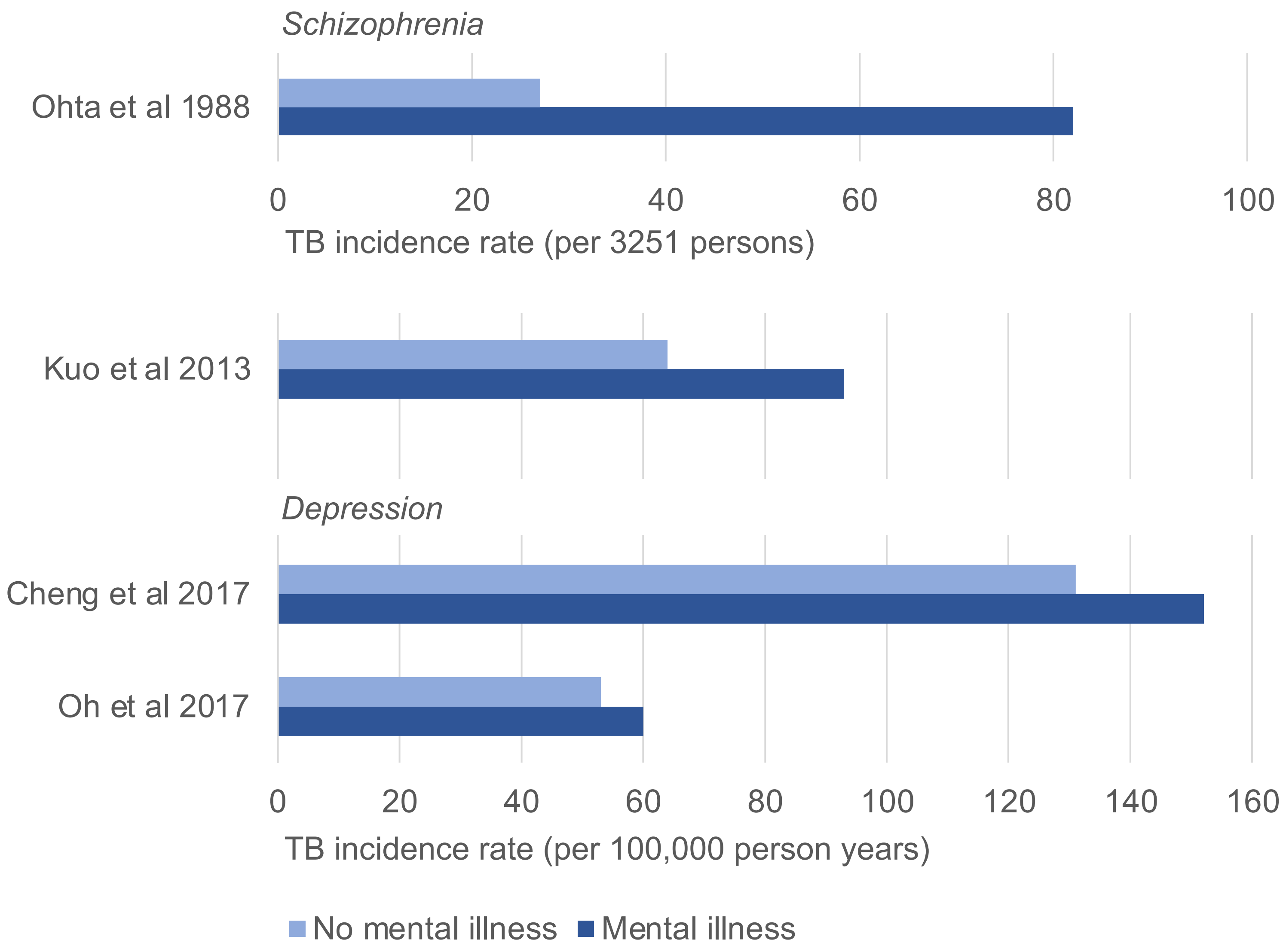
Schizophrenia
Ohta et al 1988
0
20
40
60
80
100
TB incidence rate (per 3251 persons)
Kuo et al 2013
Depression
Cheng et al 2017
Oh et al 2017
0
20
40
60
80
100
120
140
160
TB incidence rate (per 100,000 person years)
No mental illness
Mental illness

Supplementary Material

Supplementary Box 1: Search Strategy

MEDLINE Search Strategy:

1. ((Mental or Mentally or Psychological or Psychiatric) adj3 (health or illness* or disorder* or ill or unwell)).mp [mp=title, abstract, original title, name of substance word, subject heading word, keyword heading word, protocol supplementary concept word, rare disease supplementary concept word, unique identifier, synonyms]
2. exp Mental Health/
3. exp Mental Disorders/ep, et, mo, px, sn [Epidemiology, Etiology, Mortality, Psychology, Statistics & Numerical Data]
4. 1 or 2 or 3
5. (Tuberculosis or TB).mp. [mp=title, abstract, original title, name of substance word, subject heading word, keyword heading word, protocol supplementary concept word, rare disease supplementary concept word, unique identifier, synonyms]
6. exp Tuberculosis/ep, et, mo, px, sn, tm [Epidemiology, Etiology, Mortality, Psychology, Statistics & Numerical Data, Transmission]
7. 5 or 6
8. 4 and 7
9. limit 8 to English language

The PsycINFO search was the same as the MEDLINE search presented, except using subject headings for PsycINFO instead of MeSH terms. The PsycEXTRA search used the same free text terms, but this database does not use subject headings.

For peer review only

Supplementary Table 1: Critical appraisal

PART 1: Critical appraisal of cohort and case-control studies

**Cohort**	**Oh et al 2017**	**Cheng et al 2017**	**Kuo et al 2013**	**Ohta et al 1988**	**Case-control**	**de Araújo et al 2014**
**Are the results of the study valid?**						
1. Did the study address a clearly focused issue?	✓ To determine the association between depression and risk of TB	✓ To determine the association between depression and risk of pulmonary TB	✓ To determine the association between schizophrenia and risk (and outcome) of TB	✓ To investigate the incidence of TB among schizophrenics compared with the general population in Japan	1. Did the study address a clearly focused issue?	✓ To investigate the association between common mental disorders and risk of TB
2. Was the cohort recruited in an acceptable way?	✓ Nationwide population-based database, so less selection/recall bias and large sample size<br>Controls were more likely to have comorbidities than the general population as they were selected from a medical claims database – but comorbidities were adjusted for	✓ Nationwide population-based database, so less selection/recall bias and large sample size	✓ Nationwide population-based database, so less selection/recall bias and large sample size	✓ Registry of schizophrenics, so less selection/recall bias (but misses undiagnosed/ unregistered schizophrenics)	2. Did the authors use an appropriate method to answer their question?	✓ Cohort study would have provided stronger evidence for the directionality of association
3. Was the exposure accurately measured to minimise bias?	✓ ICD-10 codes for depression (requires psychiatrist's judgement from long-term observation), confirmed by prescription of psychotherapy – all psychiatrists in South Korea prescribe psychotherapy each time they see a patient under the fee-for-service system, so cases diagnosed as mild depression were also included	✓ ICD-9 codes for depression (but insurance claims not definitive measurement)	✓ ICD-9 code for schizophrenia, further validated if it was coded by psychiatrists (not clear whether this is a requirement for inclusion)	Can't tell – diagnostic criteria for schizophrenia not stated<br>Not clear whether authors are including incident or prevalent schizophrenia cases	3. Were the cases recruited in an acceptable way?	✓ Individuals diagnosed with pulmonary TB by attending chest physician at a health care unit in Salvador – excludes those who do not attend these clinics<br>Evidence of power calculation
4. Was the outcome accurately measured to minimise bias?	✓ ICD-10 codes for TB diagnosis, confirmed by prescription of two or more anti-TB drugs for simultaneous use for >30 days	✓ ICD-9 codes for pulmonary TB (but insurance claims not definitive measurement)	✓ ICD-9-CM codes for TB, plus prescription of at least two anti-TB drugs – but some bias may be introduced as any cases not prescribed anti-TB drugs will not be included	Can't tell – diagnostic criteria for TB not stated	4. Were the controls selected in an acceptable way?	× Symptomatic respiratory patients who were excluded from diagnosis of TB, age- and sex-matched – but are these patients representative of the general population?
5. a) Have the authors identified all important	× Age, gender, comorbidities (DM, COPD, alcoholism), income level	× Sex, age, comorbidities (comprehensive, but measured at baseline not during follow up)	× Age, gender, comorbidities (comprehensive)<br>But did not include any measure of SEP	× Only age and sex	5. Was the exposure accurately measured to minimise bias?	✓ Self-Reporting Questionnaire (SRQ-20), a well-recognised instrument, validated in Brazil

For peer review only

Cohort	Oh et al 2017	Cheng et al 2017	Kuo et al 2013	Ohta et al 1988	Case-control	de Araújo et al 2014
confounding factors?	Could have included more comorbidities	But did not include any measure of SEP				Instrument applied before diagnosis of TB
b) Have they taken account of the confounding factors in the design and/or analysis?	✓ Matched or included in multivariable Cox proportional hazards model	✓ Matched or included in multivariable Cox proportional hazards model	✓ Matched or included in multivariable Cox proportional hazards model	× Separate RRs calculated by sex and born before/after 1925, but no other factors considered and RR is not adjusted	6. a) What confounding factors have the authors accounted for?	✓ Marital status, ethnicity, history of contact with TB, diabetes, monthly family income, type of residence, number of household goods, level of education, crowding, recreational drugs, smoking, and alcohol consumption
6. a) Was the follow up of subjects complete enough?	✓ Nationwide database	✓ Nationwide database	✓ Nationwide database	✓ All TB cases must legally have been registered to public health centers in Nagasaki	b) Have they taken account of the potential confounding factors in the design and/or in their analysis?	✓ Matched or included in multivariable conditional logistic regression model
b) Was the follow up of subjects long enough?	✓ Followed up over up to 11 years	✓ Mean follow up 8.21 years depression and 8.30 years controls	✓ Median follow up 2,368 days for schizophrenics and 2,296 for controls	✓ (Up to?) 19 years		
**What are the results?**						
7. What are the results of this study?	Patients with depression are at a higher risk for TB (HR=2.63), with a dose-response relationship	Patients with depression are at a higher risk for TB (HR=1.15)	Schizophrenics are at a higher risk for TB (HR=1.52)	RR for TB incidence observed in those with schizophrenia vs. expected based on general population rates = 3.04	7. What are the results of this study?	Statistically significant association between overall CMDs and TB (adjusted OR=1.34)
8. How precise are the results?	HR=2.63 (95% CI 1.74-3.96)	HR=1.15 (95% CI 1.03-1.28)	HR=1.52 (95% CI 1.29-1.79, P<0.001)	No CIs given, P<0.005	8. How precise are the results? How precise is the estimate of risk?	Adjusted OR=1.34 (95% CI 1.05-1.70)<br>Overall P value not given
9. Do you believe the results?	✓ Big effect (CI does not cross 1), dose-response, study design seems sound	✓ Although effect isn't huge, CI is quite narrow and does not cross 1 (just), study design seems sound	✓ Although effect isn't huge, CI is reasonably narrow and does not cross 1, study design seems sound	✓ I believe that TB incidence is higher in schizophrenics than the general population (big effect), but to be able to say that there is an association would have been preferable to have controls and account for confounders Also would have been better to use rate per person-years	9. Do you believe the results?	✓ Although effect isn't huge, CI is quite narrow and does not cross 1 (just)
**What are the implications?**						
10. Do the results have good external validity?	✓ General population of South Korea likely to be largely representative of other HICs	✓ General population of Taiwan likely to be largely representative of other MICs/HICs	✓ General population of Taiwan likely to be largely representative of other MICs/HICs	✓ Population of Japan likely to be largely representative of other HICs	10. Do the results have good external validity?	× While the presence of an association is informative, the OR will only be relevant to this particular comparison with other respiratory patients
11. Do the results of this study fit with	✓ Depression as a risk factor for TB	✓ Depression as a risk factor for TB	✓ Schizophrenia as a risk factor for TB	✓ Schizophrenia as a risk factor for TB	11. Do the results of this study fit with	✓ Association between CMDs and TB

For peer review only

Cohort	Oh et al 2017	Cheng et al 2017	Kuo et al 2013	Ohta et al 1988	Case-control	de Araújo et al 2014
other available evidence?					other available evidence?	
12. What are the implications of this study for the review?	Supports conclusion that mental health is a risk factor for TB	Supports conclusion that mental health is a risk factor for TB	Supports conclusion that mental health is a risk factor for TB	Supports conclusion that mental health is a risk factor for TB	12. What are the implications of this study for the review?	Supports conclusion that mental health is associated with TB
**Overall judgement**						
Score	10/11 (91%)	10/11 (91%)	10/11 (91%)	7/11 (64%)	Score	8/10 (80%)
Overall study quality	High	High	High	Moderate	Overall study quality	Moderate

PART 2: Critical appraisal of cross-sectional studies

Cross-sectional	Koyanagi et al 2017	Hernández Sarmiento et al 2013	Lasebikan & Ige 2015	Srivastava et al 1983	de Castro-Silva et al 2019
**Introduction**					
1. Were the aims/ objectives of the study clear?	✓ To assess the association between depression and TB (and impact on health status) in LMICs	✓ To assess TB incidence, transmission patterns and association with socio-demographic factors and mental disorders in Colombian homeless people	✓ To determine the prevalence of psychosis in TB patients compared with controls (and its correlation with disease pattern)	✓ To discuss the psychological symptoms encountered in TB	✓ To estimate the prevalence of current major depressive episode (MDE) among patients with presumptive pulmonary TB (PTB) and compare it between patients with and without PTB
**Methods**					
2. Was the study design appropriate for the stated aim(s)?	✓ Cohort study would have provided evidence for the directionality of association	✓ Cohort study would have provided evidence for the directionality of association	✓ Cohort study would have provided evidence for the directionality of association	✓ Cohort study would have provided evidence for the directionality of association	✓ Cohort study would have provided evidence for the directionality of association
3. Was the sample size justified?	✓ Large sample (242,952), sample size determined by data available	× No justification given (relatively small sample size of 426)	× No justification given (small sample size of 227)	× No justification given (very small sample size of 120)	× No justification given (relatively small sample size of 260)
4. Was the target/reference population clearly defined? (Is it clear who the research was about?)	✓ General population of LMICs	✓ Homeless people in Medellín, Columbia	✓ Ibadan, Nigeria	✓ Population of NW districts of Rajasthan and adjoining districts of Punjab and Haryana states	✓ Presumptive pulmonary TB patients at a municipal health center in Rio de Janerio, Brazil
5. Was the sample frame taken from an appropriate population base so that it closely represented the target/reference population under investigation?	× Large sample size, predominantly nationally representative data (except 6 countries) But eligible participants >18 years and have a valid home address – excludes those without a valid home address, e.g. homeless and institutionalised who are at a high risk of TB	× Homeless people who attended a local health entity in Medellín city, so excludes those who do not attend the facility	× Those attending the MDR-TB clinic may not be representative of all TB patients in Ibadan, and their family members/caregivers may not be representative of general population	× Hospital for TB and chest diseases, Bikaner, so excludes those who are undiagnosed or not admitted to this hospital	× Sample is presumptive pulmonary TB patients (presenting with a cough lasting 3 weeks or longer), so controls without TB may not be representative of general population
6. Was the selection process likely to select	✓ Single-stage random sampling (10 countries)	× Non-probabilistic sampling	× Exclusion criteria may introduce bias e.g. excluding	× Extensive exclusion criteria e.g. patients whose general condition	✓ Exclusion criteria are reasonable

For peer review only - http://bmjopen.bmj.com/site/about/guidelines.xhtml

Cross-sectional	Koyanagi et al 2017	Hernández Sarmiento et al 2013	Lasebikan & Ige 2015	Srivastava et al 1983	de Castro-Silva et al 2019
subjects/participants that were representative of the target/reference population under investigation?	Multi-stage random cluster sampling (60 countries)		those who were not literate in English or Yoruba and those with any affective disorder, affective psychosis or delirium, and excluding family members/caregivers with a past or current history of TB or any psychiatric disorder	was considered to be poor by the treating physician and patients who gave a history of previous psychotic illness or drug abuse	
7. Were measures undertaken to address and categorise non-responders?	✓ Sampling weights generated to adjust for non-response	× No information given on response rate or non-responders	× No information given on response rate or non-responders	× No information given on response rate or non-responders	× No data available on characteristics of non-responders
8. Were the risk factor and outcome variables measured appropriate to the aims of the study?	✓ Risk factor – depressive symptoms<br>Outcome – TB	✓ Risk factor – mental disorders<br>Outcome – pulmonary TB	✓ Risk factor – psychosis<br>Outcome – MDR-TB	✓ Risk factor – psychological state<br>Outcome – pulmonary TB	✓ Risk factor – depressive symptoms, major depressive episode<br>Outcome – pulmonary TB
9. Were the risk factor and outcome variables measured correctly using instruments/measurements that had been trialled, piloted or published previously?	× Risk factor – World Mental Health Survey version of the Composite International Diagnostic Interview (used previously)<br>Outcome – past 12-month symptoms of active TB (used previously, but sensitivity 65-70% and specificity 55-75% in detecting TB)	✓ Risk factor – Mini-International Neuropsychiatric Interview (pilot experiment)<br>Outcome – medical screening examination, sputum sample analysed in those with compatible symptoms	✓ Risk factor – GHQ-12 to screen for psychiatric morbidity (good internal consistency, validated and used in Nigeria), followed by psychosis screening questionnaire for those who screened positive (valid, used in Nigeria) and psychosis module of SCID<br>Outcome – patients attending the MDR-TB treatment center (culture and drug susceptibility test-proven cases)<br>Pilot study carried out on 15 patients and 15 relatives in a different hospital in Ibadan	✓ Risk factor – Present State Examination (PSE), no evidence of having been validated<br>Considering that if a patient has symptoms in all 6 PSE subgroups he/she must have a diagnosable psychiatric illness not justified<br>Symptoms included difficulty in breathing, weight loss – but surely this is more likely due to the TB?<br>Outcome – TB diagnostic criteria not stated	✓ Risk factor – PHQ-9 and MINI-Plus; both are recognised tools and PHQ-9 has been validated in Brazil; administered by trained interviewers<br>Outcome – pulmonary TB diagnosed following the recommendations of the Brazilian national TB program
10. Is it clear what was used to determined statistical significance and/or precision estimates? (e.g. p-values, confidence intervals)	✓ Level of statistical significance set at P<0.05<br>P values and 95% CIs reported	✓ Level of statistical significance set at P<0.05<br>P values and 95% CIs reported	✓ Level of statistical significance set at P<0.05<br>P values reported	× Not clear, but from table it seems that level of statistical significance was set at P<0.05	✓ Level of statistical significance set at P<0.05<br>P values and 95% CIs reported
11. Were the methods (including statistical methods) sufficiently described to enable them to be repeated?	✓ Detailed description of methods, with references to other publications with further details	× Methods of enrolment inadequately described (need for clarification of 'non-probabilistic sample')	× Methods somewhat unclear regarding patient recruitment (consecutive? random?), screening for psychosis (psychosis screening questionnaire vs. SCID), and statistical analysis (how were ORs calculated?)	× Methods of recruitment not clear (1-2 patients out of ~5 admissions per day, no mention of how controls were recruited), no information on TB diagnostic criteria, no explanation of statistical methods used	✓ Detailed description of methods

For peer review only - http://bmjopen.bmj.com/site/about/guidelines.xhtml

Cross-sectional	Koyanagi et al 2017	Hernández Sarmiento et al 2013	Lasebikan & Ige 2015	Srivastava et al 1983	de Castro-Silva et al 2019
			It is stated that participants were periodically reviewed to detect new cases of psychosis while on admission in the MDR treatment facility, but it is not clear how this is included in the analysis		
**Results**					
12. Were the basic data adequately described?	✓ Detailed table of sample characteristics	✓ Detailed table of socio-demographic variables	✓ Detailed table of socio-demographic characteristics of patients and family members/caregivers	× Only information on age and gender given	✓ Detailed tables of socio-demographic variables
13. Does the response rate raise concerns about non-response bias?	✓(No) Response rate 98.5% (but there is substantial missing data for some variables, >10% for TB, BMI and diabetes)	×(Yes) Response rate not stated	×(Yes) Response rate not stated	×(Yes) No information given on response rate	×(Yes) Of 3,251 presumptive PTB patients, only 260 were included in the sample
14. If appropriate, was information about non-responders described?	× But it is reported that sampling weights were generated using the population distribution to adjust for non-response, so this is not a big concern	× No information given on non-responders	× No information given on non-responders	× No information given on non-responders	× No data available on characteristics of non-responders
15. Were the results internally consistent?	✓ No apparent inconsistencies	✓ No apparent inconsistencies	✓ No apparent inconsistencies	× Number in each age group adds up to 70, when sample size is 60	× It is reported in the text that 98/260 participants were diagnosed with active PTB, but in Table 1 this is reported as 99/260
16. Were the results presented for all the analyses described in the methods?	✓ Yes	× In the bivariate and multivariate analysis only significant associations (between socio-demographics/psychiatric disorders and PTB) were reported	× It seems that only ORs that gave significant results were reported when looking at correlates of psychosis in TB patients	× Statistical methods were not described<br>Other PSE symptoms that did not reach statistical significance were not reported<br>Some findings seem to be alluded to in the discussion that were not reported in the results	× Sociodemographics only presented for factors that are associated with diagnosis of TB or current MDE, so some variables not reported e.g. family income
**Discussion**					
17. Were the authors' discussions and conclusions justified by the results?	✓ TB is associated with the entire depression spectrum in the overall sample, and the association is comparable across regions and country income levels<br>Lots of potential confounders included in analysis (although could have included more comorbidities)	✓ Homeless people are a population with a high TB prevalence which co-exists with a high rate of mental disorders<br>Seems a fair conclusion, and the authors also recognise that dysthymia is the only psychiatric disorder that remains associated with risk of TB when adjusting for confounders	× Prevalence of psychosis among TB patients in this study is 33.0% compared to 2.7% among primary care givers<br>Without any adjustment for confounding it is hard to draw any conclusions from this figure, especially considering substantial baseline imbalances	✓ Significantly more patients as compared to control subjects are worried, anxious, depressed, irritable and have sleep and appetite disturbances<br>While this statement is true in this study, it is not possible to say whether there is an association here without accounting for confounding	✓ The prevalence of MDE (detected by the PHQ-9) among individuals with pre-PTB was high, and there was little difference between individuals with confirmed PTB and those with other respiratory illnesses (60.2 vs. 62.1%, respectively)<br>However, as controls have other respiratory illnesses it is not

For peer review only - http://bmjopen.bmj.com/site/about/guidelines.xhtml

Cross-sectional	Koyanagi et al 2017	Hernández Sarmiento et al 2013	Lasebikan & Ige 2015	Srivastava et al 1983	de Castro-Silva et al 2019
		No adjustment for comorbidities is an issue<br>The significant results that arise may be due to multiple testing			possible to say the prevalence of depression is the same or different in TB patients compared with the general population
18. Were the limitations of the study discussed?	✓ Lacking information on HIV, based on TB symptoms not lab diagnosis, exclusion of institutionalised and homeless	✓ Difficulty differentiating primary psychotic and affective symptoms from secondary psychotic symptoms, only patients from one homeless shelter	✓ Small sample size, only those who scored positively on GHQ were assessed for psychosis, exclusion of patients with any affective disorder, affective psychosis or delirium, only one treatment centre included	× Limitations not discussed	✓ Loss of subjects who did not return for sputum culture, small sample size, inability to analyse causal relationships, MINI-Plus only used on subset of individuals
**Other**					
19. Were there any funding sources or conflicts of interest that may affect the authors' interpretation of the results?	✓(No) Funders had no role in study design, data collection, analysis and interpretation, or in writing manuscript	✓(No) None declared	✓(No) No conflict of interest declared (but perhaps there is one, since funder is Pan African Mental Health Initiative, Nigeria?)	×(Possibly) Not stated	✓(No) No conflict of interest declared
20. Was ethical approval or consent of participants attained?	✓ All participants gave informed consent	✓ All participants signed informed consent	✓ Only consenting participants included	× Not stated	✓ All participants signed informed consent
**Overall judgement**					
Score	17/20 (85%)	12/20 (60%)	11/20 (55%)	5/20 (25%)	13/20 (65%)
Overall study quality	Moderate	Low	Low	Low	Moderate

For peer review only

Supplementary Table 2: Details of Included Studies

PART 1	Oh et al 2017	Cheng et al 2017	Kuo et al 2013	Ohta et al 1988	de Araújo et al 2014
**General Information**					
Report title	Depression and risk of tuberculosis: a nationwide population-based cohort study	Increased Risk of Pulmonary Tuberculosis in Patients with Depression: A Cohort Study in Taiwan	Incidence and outcome of newly-diagnosed tuberculosis in schizophrenics: a 12-year, nationwide, retrospective longitudinal study	The Epidemiological Study of Physical Morbidity in Schizophrenics—2. Association between Schizophrenia and Incidence of Tuberculosis	Common Mental Disorders Associated with Tuberculosis: A Matched Case-Control Study
Study funding source	Not stated	Taiwan Ministry of Health and Welfare Clinical Trial Center, China Medical University Hospital, Academia Sinica Taiwan Biobank Stroke Biosignature Project, Taiwan Clinical Trial Consortium for Stroke, Tseng-Lien Lin Foundatin, Taichung, Taiwan, Taiwan Brain Disease Foundation, Taipei, Taiwan, and Katsuzo and Kiyo Aoshima Memorial Funds, Japan	National Health Research Institute, Taipei Veterans General Hospital, and the National Science Council	Not stated	National Council for Scientific and Technological Development
Possible conflicts of interest	None declared	Not stated	None declared	Not stated	None declared
**Study Characteristics**					
Type of study	Cohort, retrospective	Cohort, retrospective	Cohort, retrospective	Cohort, retrospective (longitudinal registry)	Matched case-control
Exposure	Depression	Depression	Schizophrenia	Schizophrenia	Common Mental Disorders
Outcome	TB incidence	Pulmonary TB incidence	TB incidence (and outcomes)	TB incidence	Pulmonary TB cases
**Population and Setting**					
Population description	South Korean population who made claims through the National Health Insurance Service	Taiwanese population covered by the National Health Insurance Program	Taiwanese population covered by National Health Insurance program	Those with schizophrenia living in Nagasaki, Japan	Symptomatic respiratory patients
Setting	Korean National Health Insurance Service-National Sample Cohort (NHIS-NSC), which sampled 1 million subjects with national representativeness from among the entire population covered by NHIS in 2002	Database of the Taiwan National Health Insurance Program	NHI Research Database (NHIRD), which is run by the National Health Research Institute	Nagasaki, Japan	Three referral hospitals and six community clinics in Salvador, Brazil
Inclusion criteria	Patients with depression newly diagnosed between 2003 and 2013 were selected from the NHIS-NSC to form the exposure cohort	Subjects aged 20-84 years with newly diagnosed depression from 2000 to 2012 selected into the depression group	Schizophrenic cohort comprised all patients who were admitted with schizophrenia from 1 Jan 1998 to 31 Dec 2009	All schizophrenic patients who resided in Nagasaki and were diagnosed as schizophrenic between 1960 and 1978	Individuals living in Salvador who were older than 14 years and who presented with respiratory symptoms and were investigated

For peer review only - http://bmjopen.bmj.com/site/about/guidelines.xhtml

PART 1	Oh et al 2017	Cheng et al 2017	Kuo et al 2013	Ohta et al 1988	de Araújo et al 2014
	The control cohort comprised the same number of individuals without any mood disorders, with each individual age- and sex-matched to a patient in the exposure cohort	Four subjects without depression randomly selected, for each subject with depression, into the non-depression group, matched for sex, age (every 5-year interval), and comorbidities	Controls randomly selected from the Longitudinal Health Insurance Database dataset, which contains complete data of 1,000,000 randomly sampled beneficiaries from the original NHIRD, to match schizophrenia patients on age (within 2 years), gender, index date, Charlson's score, chronic pulmonary disease, diabetes, and rheumatoid disease		for TB at any of several health care units
Exclusion criteria	Patients with a diagnosis of TB who used medical services in 2002 were excluded to rule out chronic conditions of depression or TB<br>Patients diagnosed with TB before the diagnosis of depression were excluded from the exposure cohort	Not stated	Schizophrenia group: patients who were diagnosed with TB before being admitted with schizophrenia were excluded<br>Control group: patients who were diagnosed with schizophrenia and antecedent TB were excluded	None stated	Those reporting a previous history of active TB
Method/s of recruitment	NHIS-NSC database	Database of the Taiwan National Health Insurance Program	Inpatient and outpatient claims data from NHIRD (National Health Insurance Research Database) in Taiwan	Registry of schizophrenic patients (from all psychiatric institutes in Nagasaki city and its suburbs)	All cases asked to participate, and the first eligible control who was an acceptable age and sex match was recruited into the study
**Methods**					
Aim of study	To determine the association between depression and risk of TB	To examine the causative relationship between depression and pulmonary TB in Taiwan	To compare the adjusted incidence and outcome of TB diseases in schizophrenics and the general population	To investigate the incidence of TB among schizophrenics	To investigate the association between common mental disorders and TB
Design	Retrospective cohort study using a nationwide database to identify incidence of TB in those with depression vs. controls	Retrospective cohort study using a nationwide database to identify incidence of TB in those with depression vs. controls	Retrospective cohort study using a nationwide database to identify incidence of TB in those with schizophrenia vs. controls	Registry data to identify TB incidence in those diagnosed with schizophrenia (1960-1978) compared with expected incidence based on annual incidence rate	Matched case-control study
Start date	2003	2000	January 1998	1960	August 2008
End date	2013	2013	December 2009	1978	April 2010
Duration of participation	Up to 11 years	All subjects followed up until they were diagnosed with pulmonary TB or to the end of 2013 – so up to 14 years, but mean follow-up is 8.21 years for depression and 8.30 for controls	From index date until end of December 2009, death, or diagnosis of TB (so up to 12 years) – median follow-up 2368 days (6.48 years)	(Up to?) 19 years	N/A
**Participants**					
Total no.	64,744	172,952	120,818	3251	1434 (717 cases, 717 controls)

For peer review only - http://bmjopen.bmj.com/site/about/guidelines.xhtml

PART 1	Oh et al 2017	Cheng et al 2017	Kuo et al 2013	Ohta et al 1988	de Araújo et al 2014
Baseline imbalances	See below	See below	Imbalances in some of the comorbidities that weren't matched e.g. drug or substance abuse 4.5% in schizophrenics vs. 0.02% in controls	Not stated	No differences in the matching variables, not stated for other variables
Withdrawals and exclusions	24 excluded as they were diagnosed with TB before the diagnosis of depression	Not stated	Not stated	Not stated	All individuals agreed to participate
Age	Mean 45.8 (SD 18.4 years)	Mean 47.9 (SD 16.5) for depression and 47.6 (16.6) for controls	Median 35.4 for schizophrenics and 35.3 for controls	More than 60% patients in their 20s to 40s when diagnosed with TB	Mean 38.2 (SD 14.2 years)
Sex	Female predominant (66%)	Male predominant (62.3%) in depression and controls	Male predominant (55.1%)	Male predominant (54.8%)	Male predominant, in cases (61.0%), and controls (60.5%)
Severity of illness	Ranging from mild to severe	Not stated	Not stated	Not stated	Not stated
Co-morbidities	0.6% DM in exposed vs. 22.6% in controls<br>0.1% COPD in exposed vs. 40.4% in controls<br>3.4% alcoholism in exposed vs. 0.6% in controls	Depression group had a higher proportion of asbestosis, CKD, HIV infection, gastrectomy, pneumoconiosis, and splenectomy than controls	Most (93.4% schizophrenics, 93.4% controls) did not have any severe comorbidities (Charlson's score < 3); most prevalent underlying diseases were peptic ulcer diseases (19.1% s, 18.0% c), chronic pulmonary disease (16.7% s, 16.7% c), and liver disease (12.9% s, 14.2% c)	Not stated	Not stated
Treatment received	Psychotherapy and anti-TB drugs (see below)	Not stated	Prescription of at least two anti-TB drugs (e.g., isoniazid, ethambutol, rifampin, pyrazinamide) for two months	Not stated	Not stated
Other relevant sociodemographics	22.4% low-income subjects in exposed vs. 25.1% in controls	Not stated	Not stated	Not stated	Not stated
Subgroups measured/reported	'Mild' and 'severe' depression	Sex, age (20-39, 40-64, 65-84), no comorbidity vs. any comorbidity	Age, gender, comorbidities (Charlson's score, diabetes, chronic pulmonary disease, rheumatoid disease, myocardial infarction, heart failure, peripheral vascular disease, peptic ulcer disease, liver disease, hemiplegia or paraplegia, chronic kidney disease, cancer, hypertension, dyslipidemia, arrhythmia, drug or substance abuse, and AIDS)	Sex, patients with schizophrenia born before and after 1925	Each of the dimensions of the SRQ-20
**Exposure**					
No. in each group	32,372 with depression, 32,372 controls	34,765 with depression, 138,187 controls	60,409 with schizophrenia, 60,409 controls	3251 with schizophrenia	513 with CMD (278 cases, 235 controls), 921 without CMD (439 cases, 482 controls)

For peer review only

PART 1	Oh et al 2017	Cheng et al 2017	Kuo et al 2013	Ohta et al 1988	de Araújo et al 2014
Measurement	ICD-10 codes for depression, confirmed by prescription of psychotherapy	ICD-9 codes for depression	Patients who were admitted with schizophrenia (ICD-9-CM code), validated if it was coded by psychiatrists	All patients who resided in the city and were diagnosed with schizophrenia between 1960 and 1978, using registry data from all psychiatric institutes in Nagasaki city and its suburbs	Self-Reporting Questionnaire (SRQ-20) to identify CMDs, individuals who scored above 7 were considered to have a CMD<br>Measured by a team of nursing technicians under the supervision of senior nurses
**Outcome**					
Time points measured/reported	2003-2013	2000-2013	1998-2009	1960-1978	Before the doctor diagnosed the presence or absence of TB
Outcome definition	ICD-10 codes for TB diagnosis, confirmed by prescription of two or more anti-TB drugs for simultaneous use for >30 days	New diagnosis of pulmonary TB during the follow-up period<br>ICD-9 codes for pulmonary TB	ICD-9-CM codes for TB, plus prescription of at least two anti-TB drugs for two months	All cases of TB (pulmonary and extrapulmonary) in the population have been registered at the public health centres in Nagasaki city according to certain legal procedures	Individuals diagnosed with pulmonary TB by smear microscopy and culture for *M. tb*<br>Controls selected from the symptomatic respiratory patients who were excluded from a diagnosis of TB
Person measuring/ reporting	Not stated	Not stated	Not stated	Not stated	Attending chest physician
**Results**					
Comparison	Depression vs. control cohort	Depression vs. control cohort	Schizophrenia vs. controls	Schizophrenia vs. general population	Those with CMD vs those without CMD
Outcome	TB incidence	Pulmonary TB incidence	New TB cases	TB incidence	Incident TB
Time point	Up to end of 2013	Up to end of 2013	Up to December 2009	1960-1978	Before the doctor diagnosed the presence or absence of TB
Results	Depression: 101 per 167,271 py (n=32,372)<br>No depression: 191 per 359,265 py (n=32,372)	Depression: 435 per 285,537 py (n=34,765)<br>No depression: 1504 per 1146,960 py (n=138,187)	Schizophrenia: 366 per 392,109 py (n=60409)<br>No schizophrenia: 241 per 379,548 py (n=60409)	Schizophrenia: 82 per 3251<br>Expected: 26.94 per 3251	CMDs: 278 per 513<br>No CMDs: 439 per 921
No. missing participants and reasons	Not stated	Not stated	Not stated	Not stated	Not stated
Any other results reported	TB incidence rate 60/100,000 py in depression cohort vs. 53/100,000 py in control cohort (P<0.0001 log-rank test)<br>Incidence rate ratio (IRR) of depression to non-depression in patients with TB = 1.14 (95% CI 0.89-1.45)<br>Risk of TB 2.63-fold (95% CI 1.74-3.96) higher in depression vs. control cohort (Cox)	Overall incidence of pulmonary TB was 1.16-fold greater in the depression group vs controls (1.52 vs. 1.31 per 1000 person years), i.e. IRR depression vs. non-depression = 1.16 (95% CI 1.12-1.21)<br>Kaplan-Meier model revealed that the depression group had a higher cumulative incidence of pulmonary TB than the non-depression group (1.68 vs. 1.50% at the end of follow-up P<0.001)	Incidence rate (per 10^5 py) = 93.3 in schizophrenics, 63.5 in controls, and 78.7 overall<br>Crude HR of TB in schizophrenics = 1.48 (95% CI 1.26-1.74, p<0.001) Adjusted HR = 1.52 (95% CI 1.29-1.79, p<0.001)<br>Kaplan-Meier analysis revealed a higher rate of newly-diagnosed TB in schizophrenics (log-rank test P<0.001)	77 patients had pulmonary TB, 3 had vertebracaries, and 1 had TB peritonitis<br>Relative risk observed vs. expected was 3.04 (P<0.005)<br>48% of the patients were diagnosed as having TB before being diagnosed as schizophrenic	Statistically significant association between overall CMDs and TB (OR: 1.34, 95% CI 1.06-1.68), which remains when adjusting for confounders (OR: 1.34, 95% CI 1.05-1.70)

For peer review only

PART 1	Oh et al 2017	Cheng et al 2017	Kuo et al 2013	Ohta et al 1988	de Araújo et al 2014
		Adjusted HR for pulmonary TB was 1.15 for depression group vs. non-depression group (95% CI 1.03, 1.28) (Cox)	Most of the diseases were pulmonary (87.7% schizophrenics and 85.5% controls)		
Subgroup analysis	IRR of depression to non-depression in patients with TB = 1.40 (95% CI 0.97-2.03) in men vs. 0.98 (95% CI 0.71-1.35) in women<br>IRR of depression to non-depression in patients with TB = 1.49 (95% CI 0.21-10.8) in DM vs. 1.17 (95% CI 0.91-1.51) in no DM<br>HR of mild depression 1.99 (95% CI 1.21-3.28, P=0.007) vs severe depression 3.08 (95% CI 2.00-4.73 P<0.0001) (Cox) – linear	Incidence of pulmonary TB, stratified by sex and age, also higher in the depression group than in controls<br>Adjusted HR of pulmonary TB was 1.37 for subjects with depression and without comorbidities compared to those without depression and without comorbidities (95% CI 1.17-1.62)	Compared with schizophrenia patients without TB, those with TB tended to have advanced age, male gender, higher Charlson's score, DM, myocardial infarction, and hypertension<br>Cox regression showed independent risks for newly-diagnosed TB to be age and male gender, while patients with hypertension were less likely to have new TB diseases	Observed number of male patients with TB was 48 (out of 1780), while expected number was 17.15 (RR=2.08, P<0.005)<br>Observed number of female patients with TB was 34 (out of 1471), while expected number was 9.78 (RR=3.48, P<0.005)<br>In schizophrenia patients born before 1925, the observed number with TB was 32 (out of 679), while expected number was 13.19 (RR=2.43, p<0.005), whereas in schizophrenia patients born after 1925, the observed number with TB was 50 (out of 2572), while expected number was 17.25 (RR=2.90, p<0.005)<br>Patients born after 1925 reached the greatest risk for the onset of schizophrenia around 1950, when antipsychotic drugs became widely available and outpatient treatment became more common, but relative risk of TB remained high despite these changes	9 of the 20 SRQ-20 symptoms were individually associated with TB (feeling sad recently, crying more than usual, frequent headaches, lack of appetite, feeling tired easily, difficulty feeling satisfied with tasks, feels constantly tired, feelings of uselessness, and loss of interest in things)
Statistical methods used	Kaplan-Meier to produce cumulative TB incidence curves<br>Log-rank test to analyse differences between the two cohorts<br>Incidence rate ratio of depression to non-depression and stratified<br>Multivariable Cox proportional hazards model – hazard ratio of depression on the development of TB, also by depression severity	Incidence of pulmonary TB estimated as number of pulmonary TB events identified during follow-up divided by total follow-up person-years for each group<br>Kaplan-Meier model for cumulative incidence of pulmonary TB in depression vs. control groups<br>Initially all variables included in a univariable model, and those found to be statistically significant included in multivariable Cox proportional hazards regression model to	Incidence of TB disease was compared by Poisson distribution, and the cumulative incidence of TB was compared by the Kaplan-Meier method (log-rank test)<br>Risk factors with p values <0.1 in univariate analysis (from chi-squared, independent t-tests, Mann-Whitney U tests as appropriate) entered into the multivariate analysis, and multivariable Cox proportional hazard regression was performed using backward elimination to analyse independent risk factors	Comparison of observed and expected number of schizophrenics with TB using relative risks – expected number calculated according to annual TB incidence of general population	Pearson's chi-square test<br>Association estimated with ORs and 95% CIs using a conditional logistic regression model with backward stepwise procedures, conditional analysis for individual matching, unconditional for frequency matching<br>Variables that were associated with the outcome in the univariable analysis with p<0.25 (diabetes, alcohol abuse, ethnicity, number of household goods, level of education, history of contact and crowding) and those that contributed to the

For peer review only

PART 1	Oh et al 2017	Cheng et al 2017	Kuo et al 2013	Ohta et al 1988	de Araújo et al 2014
		estimate hazard ratio and 95% CI for risk of pulmonary TB associated with depression	for TB diseases (P<0.05 considered significant)		model's goodness-of-fit or were *a priori* confounders (drug use) and the matching variables (age and sex) were included in the model
Reanalysis	Not required	Not required	Not required	Not possible	Not required
**Applicability**					
Have important populations been excluded from the study?	No: Exposure cohort derived from a nationwide population-based sample cohort	No: Exposure cohort derived from a nationwide population-based database	No: Nationwide-population based database (99% of Taiwanese population covered by national health insurance program)	No: Includes all those diagnosed with schizophrenia in Nagasaki	Yes: Excludes those who do not attend referral hospitals or community clinics e.g. those who are too depressed to seek treatment, and controls aren't representative of general population
Does the study directly address the review question?	Yes: Depression as a risk factor for TB	Yes: Depression as a risk factor for TB	Yes: Schizophrenia as a risk factor for TB	Yes: Association between schizophrenia and TB	Yes: Common mental disorders as a risk factor for TB, but hard to establish direction of association as case-control
**Other Information**					
Key conclusions of study authors	Patients with depression are at a higher risk for TB, and a dose-response relationship exists between depression and TB risk	Patients with depression are at a significantly higher risk of pulmonary TB than patients without depression	After adjusting for underlying diseases, schizophrenics had a higher incidence of newly-diagnosed TB	The incidence rate of TB was significantly higher than that of the general population for both male and female patients with schizophrenia	There appears to be appositive and independent association between common mental disorders and TB, but can't establish direction of causation
Correspondence with study authors	Email correspondence with Dr Oh to clarify why there are fewer person-years in the depression group than the controls: because the exposure cohort were followed up from the beginning of depression diagnosis (between 2003 and 2013) whereas the controls were followed up from the start of 2003	None	None	None	None

For peer review only - http://bmjopen.bmj.com/site/about/guidelines.xhtml

PART 2	Koyanagi et al 2017	Hernández Sarmiento et al 2013	Lasebikan & Ige 2015	Srivastava et al 1983	de Castro-Silva et al 2019
**General Information**					
Report title	Depression comorbid with tuberculosis and its impact on health status: cross-sectional analysis of community-based data from 48 low- and middle-income countries	Tuberculosis Among Homeless Population from Medellín, Columbia: Associated Mental Disorders and Socio-Demographic Characteristics	Prevalence of psychosis in tuberculosis patients and their nontuberculosis family contacts in a multidrug treatment-resistant treatment center in Nigeria	Psychological Aspects of Pulmonary Tuberculosis<br>Some information about the study taken from Gupta, L.N., Bhatia, B.L., Godara, R.C. et al. (1981). Life events, physical illness and psychiatric morbidity. Ind. J. Psychiat. 23, 338-342.	Prevalence of depression among patients with presumptive pulmonary tuberculosis in Rio de Janeiro, Brazil
Study funding source	ISCIII, ERDF-FEDER, National Institute of Health Research Collaboration for Leadership in Applied Health Research & Care Funding scheme	Not stated	Pan African Mental Health Initiative, Nigeria	Not stated	National Science and Technology Institute for Tuberculosis, the Graduate Program in Clinical Medicine of the Faculty of Medicine, Universidade Federal do Rio de Janerio, and the National Institute of Mental Health (USA)
Possible conflicts of interest	None declared	None declared	None declared	Not stated	None declared
**Study Characteristics**					
Type of study	Cross-sectional (population-based)	Cross-sectional (population-based)	Cross-sectional (comparing point prevalence in cases and controls)	Cross-sectional (comparing point prevalence in cases and controls)	Cross-sectional (comparing point prevalence in cases and controls)
Exposure	Depression	Mental disorders	Psychological distress/psychotic condition	Psychological state	Depression
Outcome	TB	Pulmonary TB	MDR-TB	Pulmonary TB	Pulmonary TB
**Population and Setting**					
Population description	General population (community-based, data nationally representative for all countries except 6)	Homeless people in Medellín, Columbia	Catchment area of clinic in Ibadan, Nigeria	Population of NW districts of Rajasthan and adjoining districts of Punjab and Haryana states	Patients with presumptive pulmonary TB in Rio de Janerio, Brazil
Setting	48 LMICs	Local health facility (Centro Día Uno) in Medellín	MDR-TB treatment center under the chest unit of the Department of Medicine, University College Hospital, Ibadan	Hospital for TB and chest diseases, Bikaner	Municipal Health Center of Duque de Caxias, Rio de Janerio
Inclusion criteria	Eligible participants in the WHS were those with a valid home address and aged ≥ 18 years; one individual was randomly chosen from the household with the use of Kish tables	Homeless adults (age >18 years) who attended the facility	Consenting patients with MDR-TB (pulmonary) served as cases, and the consenting accompanying family members or caregivers served as controls	Patients with pulmonary TB and controls	Patients aged 18 and over with presumptive pulmonary TB (cough lasting 3 weeks or longer)
Exclusion criteria	Countries without full data or those that are not LMICs were deleted, leaving 48 countries	None stated	Participants who were not literate in the languages of instruction (English and Yoruba), nonconsenting patients and	Patients who had a previous admission to a TB hospital<br>Patients who were more than 50 years of age	Individuals who had received anti-TB therapy for over 7 days, or who had taken a fluoroquinolone for more than 7

For peer review only - http://bmjopen.bmj.com/site/about/guidelines.xhtml

PART 2	Koyanagi et al 2017	Hernández Sarmiento et al 2013	Lasebikan & Ige 2015	Srivastava et al 1983	de Castro-Silva et al 2019
			nonconsenting family members or caregivers<br>Family members or caregivers with a past or current history of TB or any psychiatric disorder<br>All participants with any affective disorder, affective psychosis or delirium	Patients whose general condition was considered to be poor by the treating physician<br>Patients who were taking anti-TB drugs at the time of assessment or during the preceding one month<br>Patients whose duration of illness was more than two years<br>Patients who were suffering from high grade fever at the time of assessment<br>Patients who were having concomitant other physical illness<br>Patients who gave a history of previous psychotic illness or drug abuse	days in the preceding 30 days, as well as pregnant or lactating women and individuals for whom no final diagnosis of presence or absence of TB was established, were excluded
Method/s of recruitment	World Health Survey (WHS) – cross-sectional survey carried out in 70 countries from 2002 to 2004, involving single-stage random sampling (10 countries) or multi-stage random cluster sampling (60 countries), face-to-face interviews with trained interviewers	Asked to participate – non-probabilistic sampling	Both the cases and controls were consecutively recruited into the study	1-2 patients out of approximately 5 admissions, who met these screening criteria, were included each day in the study	Patients who presented to the Municipal Health Center with a complaint of cough lasting 3 weeks or longer were invited by a staff nurse to participate in the study
**Methods**					
Aim of study	To assess the association between TB and depression (and whether the co-occurrence of TB and depression confers a more pronounced decrement in health status and function compared to TB alone)	To assess TB incidence (actually prevalence?), its transmission patterns and association with socio-demographic factors and mental disorders in Colombian homeless people	To determine the prevalence of psychosis in TB patients in comparison to non-TB controls (and its correlation with disease pattern)	To discuss the psychological symptoms encountered in TB	To estimate the prevalence of current major depressive episode (MDE) among patients with presumptive pulmonary TB and compare it between patients with pulmonary TB and without pulmonary TB
Design	Cross-sectional survey	Cross-sectional survey (although authors claim that it is prospective)	Cross-sectional study (part of an ongoing prospective study)	Cross-sectional survey	Cross-sectional survey
Start date	2002	July 2006	January 2010	Not stated	July 2015
End date	2004	December 2007	November 2014	Not stated (but took place over 3 months)	December 2016
Duration of participation	N/A	N/A	N/A	N/A	N/A
**Participants**					
Total no.	242,952	426	227 (115 patients, 112 controls)	120 (60 patients, 60 controls)	260 (99 TB patients, 161 controls)

For peer review only

PART 2	Koyanagi et al 2017	Hernández Sarmiento et al 2013	Lasebikan & Ige 2015	Srivastava et al 1983	de Castro-Silva et al 2019
Baseline imbalances	Not stated	Not stated	Significant, see below	Not stated	The diagnosis of PTB was associated with a lower mean age, BMI < 18.5kg/m^2, cough duration > 8 weeks, and a lower frequency of hypertension
Withdrawals and exclusions	Overall response rate of WHS was 98.5%, and sampling weights generated to adjust for non-response	Not stated	Data were not complete for 3 controls so they were excluded (not counted as part of the 227 above)	Not stated	Of 3,251 presumptive pulmonary TB patients seen at the center during the study period, 341 (10.5%) were recruited<br>Of these, 81 (24%) were later excluded because they did not return with the results of sputum cultures<br>Among the 260 remaining subjects, 259 (99.6%) were screened for depression using the PHQ-9<br>Of those who screened positive, 97 (61%) were subsequently evaluated with the MINI-Plus
Age	Mean 38.4 (SD 16.1 years)	Mean 38.9 (SD 10.35 years)	Median age TB patients was 35 years but 42 years for controls	9 aged 15-24, 30 aged 25-34, 20 aged 35-40, and 11 aged 44+<br>(But this adds up to 70 rather than 60)	TB cases: mean 40.67 (SD 15.7 years)<br>Controls: mean 46.9 (SD 16.0 years)
Sex	Female predominant (50.8%)	Predominantly male (82.6%)	61.7% TB patients female but 82.1% controls female	40 male, 20 female	Male predominant (62.6% cases, 60.9% controls)
Severity of illness	6.9% depressive episode, 2.9% brief depressive episode, 2.6% subsyndromal depression	TB prevalence was 7.9%: of 426 enrolled, 183 had respiratory symptoms for PTB and 34 were diagnosed with PTB	36.5% mild TB, 52.2% moderate, 11.3% severe	Not stated	Cough duration > 8 weeks in 24.1% cases and 10.4% controls
Co-morbidities	3% diabetes	Not stated	Not stated	Not stated	24.2% cases had 1 or more comorbidities, vs. 19.9% controls
Treatment received	Not stated	Not stated	MDR-TB cases admitted to a 6-month intensive phase treatment followed by 12 months of ambulatory phase<br>Respondents who had diagnosable psychotic disorders were commenced on treatment	Not stated	All eligible patients were screened for PTB and treated at the facility as needed<br>All individuals with depression were referred for outpatient mental health treatment
Other relevant sociodemographics	E.g. mean household size 5.7 (SD 3.0), 56.5% rural, 26.5% no formal education	50.5% had been living on the streets 10 years or less, 54.5% had finished primary school	50.4% TB patients and 69.6% controls had no formal education	50 married, 8 unmarried, 2 widowed	Ethnicity – TB cases: 16.3% white, 36.3% black, 5.0% Asian, 40.0% mixed race, 2.5%

For peer review only - http://bmjopen.bmj.com/site/about/guidelines.xhtml

PART 2	Koyanagi et al 2017	Hernández Sarmiento et al 2013	Lasebikan & Ige 2015	Srivastava et al 1983	de Castro-Silva et al 2019
		education, 66.0% single, 55% had social relationships with other homeless people, main income sources commercial sale of goods (63%), sale of recycled goods (40%) and begging on the streets (37%)	34.8% TB patients and 53.6% controls married<br>34.8% TB patients and 49.1% controls employed<br>53.0% TB patients and 55.4% controls Islam (vs. Christianity)<br>66.1% TB patients and 66.1% controls Yoruba, 13.0% TB patients and 13.4% Hausa, 12.2% TB patients and 11.6% controls Igbo, 8.7% TB patients and 8.9% controls minority tribes	40 lower income, 16 middle income, 4 upper income<br>51 rural, 9 urban<br>51 Hindus, 9 Muslims	indigenous; controls: 20.0% white, 30.0% black, 9.2% Asian, 40.0% mixed race, 0.8% indigenous<br>Marital status – TB cases: 51.5% married; controls: 57.1% married<br>Education – TB cases: 21.0% education of at least 8 years; controls: 30.5%
Subgroups measured/reported	Region (Africa, Americas, Asia, Europe) and country income level (LICs vs. MICs)	None	Age, gender, education, marital status, employment status, religion, ethnicity, duration of illness, disease classification, disease extent, disease category	None	Sex, age, ethnicity, marital status, educational attainment, BMI, cough duration. family income, comorbidities, diabetes, drug use, hypertension, HIV status, smoking, alcoholism, homeless, contact with resident TB, incarcerated, contact with TB, living in shelter
**Exposure**					
No. in each group	205,752 (87.7%) no depression, 5238 (2.6%) subsyndromal depression, 6674 (2.9%) brief depressive episode, 13,965 (6.9%) depressive episode	77.2% drug dependent, 29.6% previous history of major depression, 62.0% suicide risk, 54.7% antisocial personality disorder, 48.8% alcohol dependence, 47.4% bipolar affective disorder, 15.7% dysthymia (persistent mild depression), 22.1% post-traumatic stress	41 with psychoses (38 in cases, 3 in controls), 186 without psychoses	60 TB patients and 60 controls	99 TB patients and 161 controls
Measurement	The severity of depressive symptoms was established based on the individual questions of the World Mental Health Survey version of the Composite International Diagnostic Interview, which assessed the duration and persistence of depressive symptoms in the past 12 months<br>Algorithms used to establish four mutually exclusive groups:	Mini-International Neuropsychiatric Interview (MINI), a simple, structured diagnostic interview that takes about 18 minutes and diagnoses mental disorders (including depression, suicide risk, PTSD, antisocial personality, dysthymia, bipolar affective disorder, drug and alcohol dependence)	All respondents were assessed for the presence of psychological distress and psychosis in the first 7 days of admission<br>Screened each patient and relative for psychiatric morbidity using General Health Questionnaire (GHQ-12), self-administered<br>All those who screened positive and a further 10% of those who screened negative were further	Interviewed with Present State Examination (PSE) for the assessment of psychiatric/psychological state – consists of a structured interview schedule that focuses on symptoms which have occurred during the preceding one month of interview	The Patient Health Questionnaire (PHQ-9) was used to screen all participants for symptoms of depression over the past 2 weeks, with a cut-off of greater than or equal to 10 points for moderate/severe depression (validated in Brazil)<br>The Mini International Neuropsychiatric Interview (MINI-Plus) major depressive disorder module was used to

For peer review only - http://bmjopen.bmj.com/site/about/guidelines.xhtml

PART 2	Koyanagi et al 2017	Hernández Sarmiento et al 2013	Lasebikan & Ige 2015	Srivastava et al 1983	de Castro-Silva et al 2019
	depressive episode, brief depressive episode, subsyndromal depression, and no depressive disorder		assessed using the Psychosis Screening Questionnaire<br>Psychosis module of the Structured Clinical Interview for DSM-IV Axis I Disorder was used to obtain a 12-month diagnosis of any nonaffective psychosis<br>Participants were periodically reviewed to detect new cases of psychosis while on admission in the MDR treatment facility, but this does not seem to be included in this study		confirm diagnosis of current major depressive episode in those diagnosed with depressive symptoms in the PHQ-9<br>These tools were administered by a nurse and 2 medical students
**Outcome**					
Time points measured/reported	2002-2004	July 2006-Dec 2007	Jan 2010-Nov 2014	Not stated	July 2015-Dec 2016
Outcome definition	Past 12-month symptoms of active TB, i.e. those who had both:<br>- a cough that lasted for 3 weeks or longer<br>- blood in phlegm (or coughed up blood)<br>These symptoms are likely to have a sensitivity of 65-70% and specificity of 55-75% in detecting TB	Medical screening examination to identify patients with respiratory symptoms compatible with pulmonary TB (cough and expectoration for 3 weeks or longer); from each subject who fulfilled the criteria, a sputum sample was taken for microscopic smear examination using conventional biochemical tests	Patients attending the MDR-TB treatment center<br>National Tuberculosis Program and WHO guidelines to assess the severity of TB, based on bacillary load, extent of disease and anatomical site that carries a significant acute threat to life and/or a risk of subsequent severe handicap	Pulmonary TB, no further details given	Pulmonary TB was diagnosed following the recommendations of the Brazilian national TB program for evaluation of patients with respiratory symptoms; patients with a positive sputum smear for acid-fast bacilli and/or a positive rapid molecular test for *M.tb* (Xpert MTB/RIF) were considered to be active TB cases; patients with negative smears and who ultimately received another diagnosis constituted the non-TB group
Person measuring/ reporting	Trained interviewers	Trained professionals (physicians, nurses and social workers)	Not stated	Not stated	Not stated
**Results**					
Comparison	Prevalence of depression in those with and without TB	With and without psychiatric disorders	Prevalence of psychoses in those with and without MDR-TB	Psychological symptoms in TB patients compared with controls	Depression and major depressive episode (MDE) in TB patients compared with controls
Outcome	Authors treat depression as outcome	Pulmonary TB diagnosis	Authors treat psychoses as outcome	Authors treat psychological symptoms as outcome	Authors treat depression and MDE as outcomes
Time point	2002-2004	July 2006-Dec 2007	Jan 2010-Nov 2014	Not stated	July 2015-Dec 2016
Results	TB cases: 23.7% of 3347<br>Controls: 6.8% of 196,417	Raw data not reported (see below for ORs)	MRD-TB cases: 38 per 115<br>Controls: 3 per 112	TB cases: 25 per 60<br>Controls: 8 per 60	In the screening test for depression, the proportion of TB and non-TB patients with a PHQ-9 score greater than or equal to 10

For peer review only - http://bmjopen.bmj.com/site/about/guidelines.xhtml

PART 2	Koyanagi et al 2017	Hernández Sarmiento et al 2013	Lasebikan & Ige 2015	Srivastava et al 1983	de Castro-Silva et al 2019
					was 60.2 vs. 62.1% (OR = 0.92, 95%CI 0.55-1.54, p = 0.79); among the patients with a PHQ-9 score greater than or equal to 10 who were subsequently assessed with the MINI-Plus, current MDE was identified in 59.5% of TB and 50.9% of non-TB patients, with no statistically significant difference (OR = 1.42, 95%CI 0.63-3.19, p = 0.42)
No. missing participants and reasons	17.7% data missing for TB, also over 10% missing data for BMI and diabetes, so missing values imputed for regression analyses	Not stated	Data were not complete for 3 controls so they were excluded	Not stated	Of 341 initially recruited, 81 (24%) were excluded because they did not return with the results of sputum cultures; of 260 remaining, 1 did not complete PHQ-9; of 159 who screened positive on PHQ-9, 62 were not evaluated with MINI-PLUS
Any other results reported	All types of depression were more frequent among those with TB, especially for depressive episode: prevalence of depressive episode was 23.7% (95% CI 20.5-27.1) with TB and 6.8% (95% CI 6.5-7.1) without TB (P<0.001) Multivariable multinomial logistic regression showed that TB is associated with a 1.98 (95% CI 1.47-2.67, P<0.0001), 1.75 (95% CI 1.26-2.42, P=0.0008) and 3.68 (95% CI 3.01-4.50, P<0.0001) times higher odds for subsyndromal depression, brief depressive episode, and depressive episode, respectively	Those with dysthymia had 2.66 times the odds (1.19-5.19, p=0.013) of pulmonary TB diagnosis and for those with previous history of major depression the OR was 2.22 (1.07-4.6, p=0.028)<br>In the multivariable model the adjusted OR for dysthymia was 2.54 (1.10-5.86, p=0.028)<br>Results for other mental disorders not reported: although an association between several mental disorders including major depression and having pulmonary TB was found by bivariate analysis, dysthymia was the only psychiatric disorder found to be associated with risk of having TB in the regression model analysis<br>13.4% reported having been previously diagnosed with TB, so perhaps issue of reverse causality<br>Incidence of TB in homeless population overall was 7.9% (34/426)	81.7% of TB patients scored positively on GHQ vs. 51.7% controls (P<0.001)<br>8.7% TB patients met DSM-IV criteria for schizophrenia vs. 0.9% controls<br>9.6% TB patients met DSM-IV criteria for other nonaffective psychosis vs. 1.8% controls<br>14.8% TB patients had anti-TB medication-induced psychotic disorder<br>33.0% TB patients had any psychosis vs. 2.7% controls	86.6% patients (52/60) had one or other psychiatric symptoms which could be rated as positive, compared with 70% controls (42/60)<br>41.6% patients (25/60) had symptoms in all six PSE subgroups, suggesting a diagnosable psychiatric illness, compared with 13.3% controls (8/60) (P<0.001)<br>Worries, tension headache, free floating anxiety, depressed mood, sleep and appetite disturbances, subjective anergia, retardation and irritability are the PSE symptoms revealed by significantly more TB patients compared with controls<br>None of the patients had psychotic symptoms (due to exclusion criteria?)<br>Other PSE symptoms that did not reach statistical significance were not reported	In both groups, the most frequently reported symptoms were depressed mood, sleep disturbance, fatigue, and appetite change. Individuals with PTB were more likely than those without PTB to endorse reduced appetite (87 vs. 73%, p = 0.009)<br><br>Loss to follow-up was higher among individuals with MDE (8.7%) compared to those without MDE (4.3%); however, this difference was not statistically significant. There were also no statistically significant associations of alcohol, drug abuse, or lower education with current MDE

For peer review only - http://bmjopen.bmj.com/site/about/guidelines.xhtml

PART 2	Koyanagi et al 2017	Hernández Sarmiento et al 2013	Lasebikan & Ige 2015	Srivastava et al 1983	de Castro-Silva et al 2019
				~50% patients reported being sad and anxious because of illness	
Subgroup analysis	TB was associated with a depressive episode across regions and country income levels although the estimates for Europe did not reach statistical significance, possibly due to lack of statistical power	None	Proportion of TB patients with positive GHQ and psychosis increased with increasing age, a higher proportion had a duration of illness ≥ 4 years, had extrapulmonary TB, had moderate or severe disease, and had category 2 TB	None	Women were two and a half times more likely to present with current MDE than men were (OR = 2.52, 95%CI, 1.10-5.76; p = 0.04); being female remained independently associated with MDE in logistic regression (OR = 2.72, 95%CI 1.39-5.30; p = 0.003)
Statistical methods used	Multivariable multinomial logistic regression analyses using the overall sample, and multivariable binary logistic regression while stratifying by region or country income level<br>All regression analyses adjusted for age, sex, education, wealth, household size, setting, current smoking, alcohol consumption, BMI, diabetes, and country<br>The sample weighting and complex study design were taken into account in all analyses	Bivariate analysis for the independent variables with their odds ratio and 95% CI for presence or absence of pulmonary TB<br>Multivariate analysis using an exploratory logistic regression with forward modelling was performed to determine the association between independent variables and pulmonary TB, using Hosmer-Lemeshow test to adjust the logistic regression model<br>Variables included in logistic regression model: income from drugs trade, social relationships with other homeless, receive food from other homeless, receive clothes from other homeless, live with homeless friends, stable relationships in couples, previous history of major depression, dysthymia	Chi-square test or Fisher's exact test for categorical variables<br>Mann-Whitney U test for nonparametric data<br>All chi-square p values were Bonferroni corrected where necessary	Chi-squared tests	Absolute and relative frequencies were calculated for categorical variables. Continuous variables are presented as mean and standard deviation. Associations of categorical variables with TB and MDE were evaluated using the chi-square test (or Fisher's exact test, when indicated); ORs were derived and 95% CIs calculated. Student's t-test was used to compare continuous variables. Logistic regression was used to control for variables independently associated with MDE. Statistical significance was accepted when p is greater than or equal to 0.05 (two-tailed)
Reanalysis	Not possible	Not required	Removing the 17 patients with medication-induced psychotic disorders, prevalence of psychosis in MDR-TB patients = 18.3% (21/115)	Not possible	Not required

For peer review only

For peer review only - http://bmjopen.bmj.com/site/about/guidelines.xhtml

PART 2	Koyanagi et al 2017	Hernández Sarmiento et al 2013	Lasebikan & Ige 2015	Srivastava et al 1983	de Castro-Silva et al 2019
**Applicability**					
Have important populations been excluded from the study?	No: Community-based survey (although excludes those without a valid home address, e.g. the institutionalised and the homeless who are at a high risk of TB)	Possibly: Only those homeless people attending one local health facility included	Yes: Only MDR-TB, and only one treatment centre<br>Are caregivers really representative controls?<br>Exclusion of participants with any affective disorder, affective psychosis or delirium could have distorted the prevalence<br>Also the exclusion of family members/caregivers with any psychiatric disorder	Yes: Extensive exclusion criteria with no justification	Possibly: Using patients of other respiratory illnesses as controls may not be representative
Does the study directly address the review question?	No: Treating depression as outcome and TB as exposure<br>Direction of causality cannot be established due to cross-sectional design	Yes: But direction of causality cannot be established due to cross-sectional design	No: Treating psychosis as outcome and MDR-TB as exposure<br>Direction of causality cannot be established due to cross-sectional design	No: Treating psychological state as outcome and TB as exposure<br>Direction of causality cannot be established due to cross-sectional design	Yes: But direction of causality cannot be established due to cross-sectional design
**Other Information**					
Key conclusions of study authors	TB is associated with the entire depression spectrum in the overall sample, and the association is comparable across regions and country income levels	Homeless people constitute a particular population with a high TB prevalence which co-exists with a high rate of mental disorders	Prevalence of psychosis among TB patients in this study is 33.0% (18.3% excluding medication-induced psychotic disorders compared to 2.7% among primary care givers	Significantly more patients as compared to control subjects are worried, anxious, depressed, irritable and have sleep and appetite disturbances	The prevalence of MDE (detected by the PHQ-9) among individuals with pre-PTB was high, and there was little difference between individuals with confirmed PTB and those with other respiratory illnesses (60.2 vs. 62.1%, respectively)
Correspondence with study authors	None	None	None	None	None

For peer review only

PRISMA 2009 Checklist

Section/topic	#	Checklist item	Reported on page #
**TITLE**			
Title	1	Identify the report as a systematic review, meta-analysis, or both.	1
**ABSTRACT**			
Structured summary	2	Provide a structured summary including, as applicable: background; objectives; data sources; study eligibility criteria, participants, and interventions; study appraisal and synthesis methods; results; limitations; conclusions and implications of key findings; systematic review registration number.	2-3
**INTRODUCTION**			
Rationale	3	Describe the rationale for the review in the context of what is already known.	4-5
Objectives	4	Provide an explicit statement of questions being addressed with reference to participants, interventions, comparisons, outcomes, and study design (PICOS).	5-6 and Table 1
**METHODS**			
Protocol and registration	5	Indicate if a review protocol exists, if and where it can be accessed (e.g., Web address), and, if available, provide registration information including registration number.	5
Eligibility criteria	6	Specify study characteristics (e.g., PICOS, length of follow-up) and report characteristics (e.g., years considered, language, publication status) used as criteria for eligibility, giving rationale.	5-6
Information sources	7	Describe all information sources (e.g., databases with dates of coverage, contact with study authors to identify additional studies) in the search and date last searched.	5
Search	8	Present full electronic search strategy for at least one database, including any limits used, such that it could be repeated.	Supp Box 1
Study selection	9	State the process for selecting studies (i.e., screening, eligibility, included in systematic review, and, if applicable, included in the meta-analysis).	5-6
Data collection process	10	Describe method of data extraction from reports (e.g., piloted forms, independently, in duplicate) and any processes for obtaining and confirming data from investigators.	7
Data items	11	List and define all variables for which data were sought (e.g., PICOS, funding sources) and any assumptions and simplifications made.	7
Risk of bias in individual studies	12	Describe methods used for assessing risk of bias of individual studies (including specification of whether this was done at the study or outcome level), and how this information is to be used in any data synthesis.	7
Summary measures	13	State the principal summary measures (e.g., risk ratio, difference in means).	7
Synthesis of results	14	Describe the methods of handling data and combining results of studies, if done, including measures of consistency (e.g., I^2) for each meta-analysis.	N/A

For peer review only - http://bmjopen.bmj.com/site/about/guidelines.xhtml

PRISMA 2009 Checklist

Section/topic	#	Checklist item	Reported on page #
Risk of bias across studies	15	Specify any assessment of risk of bias that may affect the cumulative evidence (e.g., publication bias, selective reporting within studies).	N/A
Additional analyses	16	Describe methods of additional analyses (e.g., sensitivity or subgroup analyses, meta-regression), if done, indicating which were pre-specified.	N/A
**RESULTS**			
Study selection	17	Give numbers of studies screened, assessed for eligibility, and included in the review, with reasons for exclusions at each stage, ideally with a flow diagram.	8 and Figure 1
Study characteristics	18	For each study, present characteristics for which data were extracted (e.g., study size, PICOS, follow-up period) and provide the citations.	Tables 2 and 3
Risk of bias within studies	19	Present data on risk of bias of each study and, if available, any outcome level assessment (see item 12).	Table 2
Results of individual studies	20	For all outcomes considered (benefits or harms), present, for each study: (a) simple summary data for each intervention group (b) effect estimates and confidence intervals, ideally with a forest plot.	Table 3
Synthesis of results	21	Present results of each meta-analysis done, including confidence intervals and measures of consistency.	N/A
Risk of bias across studies	22	Present results of any assessment of risk of bias across studies (see Item 15).	N/A
Additional analysis	23	Give results of additional analyses, if done (e.g., sensitivity or subgroup analyses, meta-regression [see Item 16]).	N/A
**DISCUSSION**			
Summary of evidence	24	Summarize the main findings including the strength of evidence for each main outcome; consider their relevance to key groups (e.g., healthcare providers, users, and policy makers).	12-15
Limitations	25	Discuss limitations at study and outcome level (e.g., risk of bias), and at review-level (e.g., incomplete retrieval of identified research, reporting bias).	13-14
Conclusions	26	Provide a general interpretation of the results in the context of other evidence, and implications for future research.	12-15
**FUNDING**			
Funding	27	Describe sources of funding for the systematic review and other support (e.g., supply of data); role of funders for the systematic review.	16

From: Moher D, Liberati A, Tetzlaff J, Altman DG, The PRISMA Group (2009). Preferred Reporting Items for Systematic Reviews and Meta-Analyses: The PRISMA Statement. PLoS Med 6(7): e1000097. doi:10.1371/journal.pmed1000097

For more information, visit: **www.prisma-statement.org**.

For peer review only - http://bmjopen.bmj.com/site/about/guidelines.xhtml
